# Time spent at or near V̇O₂max during high-intensity interval training - a systematic review and meta-analysis

**DOI:** 10.1186/s13102-026-01766-x

**Published:** 2026-06-03

**Authors:** Patrick Schoenmakers, Kelly Murray, Jamie White, Guilherme Matta, Arthur Henrique Bossi

**Affiliations:** 1 School of Sport, Rehabilitation, and Exercise Sciences, University of Essex, Colchester, CO4 3SQ United Kingdom; 2 Juiz de Fora, Minas Gerais Brazil; 3 School of Applied Sciences, Edinburgh Napier University, Edinburgh, EH11 4BN United Kingdom; 4 Mountain Bike Centre of Scotland, Glentress, Peebles EH45 8NB United Kingdom; 5 University of Regina, Faculty of Kinesiology and Health Studies, Regina, SK S4S 0A2 Canada

**Keywords:** HIIT, Exercise prescription, tV̇O_2_max, Endurance Training

## Abstract

**Background:**

High-intensity interval training (HIIT) is widely recognised as an effective strategy to improve cardiorespiratory fitness and endurance performance. Time spent at or near maximal oxygen uptake (tV̇O_2_max) has emerged as a key marker of the adaptive potential of HIIT, yet HIIT configurations that maximise tV̇O₂max remain unclear.

**Objective:**

This systematic review and meta-analysis examined acute tV̇O₂max responses across various HIIT protocols, aiming to identify configurations with the greatest potential to stimulate training adaptations.

**Methods:**

PubMed and Scopus were systematically searched to September 2025 for studies reporting tV̇O₂max during HIIT, defined as time spent at or above ≥ 80% V̇O₂max. Associations between protocol characteristics and tV̇O₂max were evaluated using ANCOVAs and linear mixed-effects models, while random-effects meta-analyses compared standardised mean differences (SMDs) across HIIT configurations, recovery modalities (active vs passive), and exercise intensity distributions in work intervals.

**Results:**

The literature search yielded 86 unique articles, in which tV̇O_2_max was determined for a total of 239 HIIT protocols. Protocols with long work intervals (≥ 2 min) elicited significantly greater tV̇O₂max than short (≤ 30 s) or moderate (> 30 s to < 2 min) work intervals, and linear mixed-effects models confirmed a positive relationship between cumulative HIIT duration and time ≥ 90% and ≥ 95% V̇O₂max when longer work intervals were used. Protocols incorporating variable-intensity work intervals further increased tV̇O₂max compared to even-paced formats (SMD = 0.80, *p* < .01). In contrast, recovery modality (active vs passive) showed no effect on tV̇O₂max.

**Conclusions:**

tV̇O₂max exhibits considerable variability, and its dose–response relationship with long-term adaptations requires further investigation. Nevertheless, HIIT protocols with longer and variable-intensity work intervals maximise tV̇O₂max, providing researchers, practitioners, and athletes with a firmer foundation for understanding how specific protocols shape the physiological demands and adaptive potential of a workout.

**Supplementary Information:**

The online version contains supplementary material available at 10.1186/s13102-026-01766-x.

## Introduction

High-intensity interval training (HIIT) is by no means a new phenomenon, but instead a training concept long employed by athletes to improve their cardiorespiratory fitness, metabolic functioning, and performance [1–4]. Pioneering work in the 1960 s on the acute physiological responses to HIIT laid the scientific foundations for the programming of HIIT sessions comprising both long and short work intervals [5, 6], with the first intervention studies emerging in the 1970 s [7, 8]. Research interest renewed from the mid-1990s [9], and HIIT is now considered a time-efficient and effective strategy to improve an individuals’ maximum oxygen uptake (V̇O_2_max), where meta-analyses have shown the magnitude of adaptations appear moderated by baseline fitness and HIIT configurations [10–13]. These meta-analyses have further shown that HIIT interventions incorporating long work intervals (≥ 2 min; LI-HIIT) elicit larger improvements in V̇O_2_max than interventions implementing repeated short (≤ 30 s; SI-HIIT) or moderate duration (> 30 s to < 2 min; MI-HIIT) work intervals [10–13]. HIIT has also been shown to enhance performance in recreational and trained athletes [13–16], with negligible differences between SI-HIIT, MI-HIIT and LI-HIIT on 30-s Wingate performance [13, 16], but greater improvements in time trial performance (i.e., 2-km rowing, 3-km running, and 40-km cycling) following LI-HIIT compared to SI-HIIT [13, 15].

V̇O_2_max is a key physiological determinant and strong predictor of endurance performance [17], and HIIT has been shown to elicit greater improvements in V̇O_2_max compared to (continuous) moderate-intensity training in interventions of similar duration [18, 19]. However, given the high demands of HIIT, only a limited proportion (~10–20%) of total training is, or can be, performed in this manner [20], as evident from training intensity distributions in recreational and trained endurance athletes [21, 22]. Accordingly, careful programming of these sessions is essential to fully realise their physiological and performance benefits.

In HIIT, repeated periods of vigorous exercise (work intervals) are alternated with recovery periods (recovery intervals), and a complex interplay between the number of repetitions and the exercise intensity and duration of both work and recovery intervals determine the physiological demand of a session [2, 3, 23]. The manipulation of these variables allows for virtually limitless customisation of HIIT protocols, enabling precise tailoring to specific training goals and individual needs. As demanding work intervals are required to drive training adaptations, multiple studies have sought to optimise the intensity and duration of work intervals (e.g. [24–26]). However, if recovery intervals do not provide sufficient respite relative to the demands of the work intervals, premature fatigue and incomplete HIIT sessions may occur [27]. Studies have thus increasingly focused on understanding the acute responses to manipulations in recovery intensities and durations as well (e.g. [28, 29]). In addition to these factors, the exercise intensity distribution within work intervals has recently been suggested to influence time spent at higher fractions of V̇O_2_max [30]. For example, in 14 well-trained cyclists, Bossi et al. [31] demonstrated that, despite being matched for mean power output and duration, variable-intensity cycling HIIT, incorporating three 30-s efforts within 5-min work intervals, elicited ~43% longer time ≥ 90% V̇O₂max compared with constant-intensity intervals. Yet, in the absence of comprehensive investigations, it remains challenging to draw definitive conclusions about the most effective HIIT configurations.

The intermittent profile of HIIT allows athletes to maximise 1) time at high work rates and 2) time at or near their V̇O_2_max (tV̇O_2_max - [3]). Typically, tV̇O_2_max, or time in the *‘red zone’*, refers to the accumulated duration in which athletes reach metabolic demands equivalent to, or above 90% V̇O_2_max. It can also be described more broadly, as the total time spent at metabolic rates that lie within the severe intensity domain, that is, above the second lactate (LT2) or ventilatory thresholds (VT2). At these exercise intensities, the oxygen delivery and utilisation systems are maximally stressed, and by doing so, HIIT can enhance the metabolic and physiological overload of a training session compared with continuous training [32–35]. This stimulus has been shown to promote central and peripheral adaptations associated with improvements in V̇O_2_max, including expansion of red blood cell and stroke volume, mitochondrial content, and skeletal muscle capillarisation [18, 36]. Whilst the dose-response relationship between tV̇O_2_max per HIIT session and subsequent improvements in physiological function and performance remains unclear, it is generally accepted that both the mean V̇O_2_ and the time sustained at high fractions of V̇O_2_max are key indicators to characterise the adaptive potential of HIIT protocols [3, 32, 37–39]. Although not yet conclusively demonstrated through empirical research, Buchheit and Laursen [3] indicated that 5–7 min of exercise in the *‘red zone’* per HIIT session may be sufficient to induce adaptations for team-sport athletes, whereas trained endurance athletes may require more than 10 min at tV̇O_2_max. Given the recognition of tV̇O_2_max as a valuable indicator of adaptive stimulus, identifying HIIT protocols that maximise tV̇O_2_max is essential to inform future research as well as training program implementation.

The present systematic review and meta-analysis aims to synthesise the available evidence from HIIT studies that have reported tV̇O_2_max. In the light of the growing variety of HIIT protocols found in the literature, spanning different exercise modalities, configurations, and athletic populations, our review seeks to update and expand upon the foundational review of Midgley and McNaughton [32], which examined tV̇O_2_max during intermittent running protocols. Furthermore, we aim to identify which HIIT configurations are most likely to meet the minimal tV̇O_2_max per session as proposed by Buchheit and Laursen [3]. This review, therefore, endeavours to inform both future research and evidence-based practice, offering refined guidance for the design of HIIT protocols that optimise physiological and performance adaptations.

## Methods

This systematic review was performed according to the latest Preferred Reporting Items for Systematic Reviews and Meta-Analyses statement [40].

### Literature search

A literature search was performed in the PubMed and Scopus databases on the 23^rd^ of September 2025. The search was conducted using the Boolean operators ‘AND’ and ‘OR’ and combinations of the following keywords: (‘tV̇O_2_max’, OR ‘t90V̇O_2_max ‘, OR ‘time near’ OR ‘time above’ OR ‘time at’) AND (‘V̇O_2_max’ OR ‘V̇O_2_peak’ OR ‘oxygen uptake’ OR ‘oxygen consumption’) AND ‘interval training’.

**Fig. 1:**
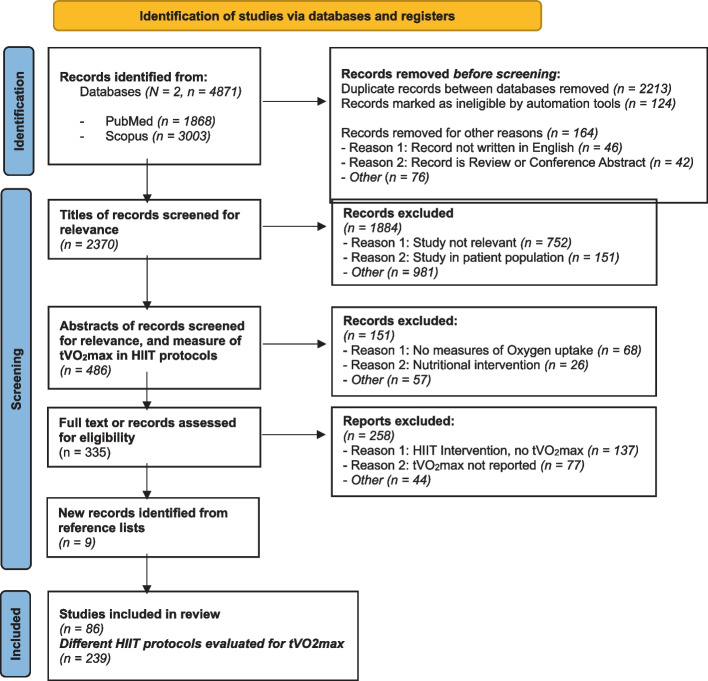
PRISMA flow diagram of systematic search process

After duplicates were removed, titles of relevant articles were screened before an examination of abstracts, which was then followed by full text reviews (see Fig. 1). To identify studies that were potentially overlooked by the database search, reference lists of included studies were screened. Only peer-reviewed journal articles, written in English, were included in this systematic review.

### Eligibility criteria and outcome measures

Studies were included if they reported tV̇O_2_max during HIIT sessions and were conducted in apparently healthy human participants. No restrictions were placed on training status and age of participants, nor the exercise modality. The primary outcome of this systematic review was tV̇O₂max. As the demarcation between the heavy and severe exercise intensity domains varies between individuals and is not fixed at 90% V̇O₂max, studies reporting time at or above 80% V̇O₂max were included.

No minimum HIIT session duration, number of intervals or work interval intensity was imposed, as the aim of the review was to capture the full range of HIIT protocols reported across the literature. Studies incorporating additional interventions (e.g. nutritional supplementation or environmental conditions such as hypoxia) were included where a control or baseline HIIT condition could be extracted independently.

Studies were excluded if they did not report tV̇O₂max, measured tV̇O₂max during only continuous (non-interval) exercise, involved clinical populations, or were not original peer-reviewed research articles written in English.

### Data extraction

Data were extracted into two dedicated spreadsheets. Firstly, descriptive data for each included study was collected: author, year of publication, exercise modality and participant characteristics (sample size, sex, age, height, body mass, V̇O_2_max, and peak exercise intensity; see Table 1). Additionally, we collated information of every HIIT protocol, including the number of work-recovery cycles (repetitions), work interval duration and intensity, recovery interval duration and intensity, cumulative HIIT protocol duration (combining the duration of both the work and recovery intervals), and the isolated total work interval duration (see Table 2). Data extraction was undertaken by PS, after which JW and KM checked the data independently. When data in any of the variables were unclear or incomplete, authors were contacted for clarification. When study authors did not respond to requests for clarification, protocol characteristics reported in the original publications were used to calculate total training- or isolated work interval durations (see Supplementary Table S1). In line with the Cochrane Handbook for Systematic Reviews of Interventions [120], sample means and standard deviations (SD) of several studies were calculated from standard errors, confidence intervals (CI), medians, and interquartile ranges using established formulae. Freely available online software was used to obtain data from studies where data were only published in figures (WebPlotDigitizer, Version 4.8).

**Table 1:** Participant characteristics, and measures of tV̇O_2_max reported in included studies

	**N**	**Sex** **(M:F)**	**Age** **(years)**	**Height** **(cm)**	**Body Mass** **(kg)**	$\dot{\mathrm{V}}{\mathrm{O}_{2}}\mathrm{max}$** (L·min**^−1^** or ml·kg·min**^−1^**)**	**Peak exercise intensity**	**Variables reported**
**Cycling**								
Zuniga et al. [41] ^A^	12	7:5	26.8 ± 4	174 ± 8	74.2 ± 9	47.6 ± 5.5	314 ± 22 W	t ≥ 95% V̇O_2_max
Nicolò et al. [42]	13	13:0	19 ± 2	174 ± 5	65 ± 5	63.1 ± 4.2	330 ± 35 W	t ≥ 90% V̇O_2_max, t ≥ 95% V̇O_2_max
Lisbôa et al. [43]	8	8:0	22.1 ± 2.9	174 ± 4.6	72.6 ± 5.3	47.6 ± 8.8	256 ± 23 W	t ≥ 90% V̇O_2_max, t ≥ 95% V̇O_2_max
Zadow et al. [44] ^B^	15	15:0	39 ± 8	181 ± 4.9	79.4 ± 8.2	59.8 ± 6.5	436 ± 27 W	t ≥ 85% V̇O_2_max
Zafeiridis et al. [45]	12	12:0	24 ± 3.2	179 ± 5	77.3 ± 9.6	49.3 ± 5.1	261 ± 29 W	t ≥ 80% V̇O_2_max
Rønnestad & Hansen [46]	13	13:0	29 ± 2	179 ± 6	73.4 ± 5.1	66.7 ± 6.1	375 ± 41 W	t ≥ 90% V̇O_2_max
Turnes et al. [39] [LOW]	11	10:1	22 ± 2	175 ± 6	76 ± 6	47.0 ± 5.4	265 ± 45 W	t ≥ V̇O_2_max – 1SD (≈ t ≥ 90% V̇O_2_max)
[UP]	10	9:1	23 ± 3	174 ± 7	78 ± 8	48.5 ± 5.4	269 ± 37 W	t ≥ V̇O_2_max – 1SD (≈ t ≥ 90% V̇O_2_max)
Combes et al. [47]	12	12:0	24 ± 5	180 ± 6	76 ± 10	43 ± 6	282 ± 31 W	t ≥ 90% V̇O_2_max
Rønnestad et al. [48]	10	10:0	24 ± 7	180 ± 5	73.2 ± 6.2	78.6 ± 6.2	471 ± 29 W	t ≥ 90% V̇O_2_max
Shi et al. [49] ^C^	13	13:0	26.2 ± 6.2	173 ± 7.3	60.8 ± 3.8	61.3 ± 9.1	NR	t ≥ 80% V̇O_2_max, t ≥ 85% V̇O_2_max, t ≥ 90% V̇O_2_max, t ≥ 95% V̇O_2_max, t ≥ V̇O_2_max
Boynton et al. [50]	11	11:0	34 ± 9.8	181 ± 5	75.8 ± 8	69.6 ± 6.7	NR	t ≥ 90% V̇O_2_max
Lisbôa et al. [51]	11	11:0	28 ± 5	175 ± 5	74 ± 7	63 ± 4	334 ± 37 W	t ≥ 95% V̇O_2_max
Almquist et al. [52] ^B^	8	8:0	25.3 ± 6.1	181 ± 4.5	74.8 ± 3.8	73.9 ± 7	6.2 ± 0.5 W·kg^−1^	t ≥ 90% V̇O_2_max
Bossi et al. [31]	14	14:0	24 ± 6	184 ± 5	75 ± 5.2	69.2 ± 6.6	430 ± 35 W	t ≥ 90% V̇O_2_max
Vaccari et al. [53]	12	12:0	41 ± 11	NR	76 ± 10	4.32 ± 0.47	356 ± 40 W	t ≥ 90% V̇O_2_max
Dall’Agnol et al. [54]	11	11:0	34.4 ± 6.2	177 ± 4.4	74.7 ± 8.1	55.7 ± 7.4	316 ± 32 W	t ≥ 90% V̇O_2_max, t ≥ 95% V̇O_2_max
Beltrami et al. [55]	15	14:1	29 ± 5	180 ± 6	74 ± 6	62 ± 6	410 ± 37 W	t ≥ 90% V̇O_2_max
Fennell & Hopker [56]	14	14:0	33 ± 13	177 ± 5.9	70.6 ± 8.1	62 ± 9	370 ± 56 W	t ≥ 80% V̇O_2_max, t ≥ 90% V̇O_2_max, t ≥ 95% V̇O_2_max
Fennell & Hopker [29]	16	16:0	32 ± 13	178 ± 5.2	72.4 ± 9.1	60 ± 7	373 ± 57 W	t ≥ 80% V̇O_2_max, t ≥ 90% V̇O_2_max, t ≥ 95% V̇O_2_max
Ksoll et al. [57]	24	24:0	24.3 ± 3.6	181 ± 5.1	75.9 ± 7.6	54.1 ± 5.3	360 ± 45 W	t ≥ 80% V̇O_2_max
McKee et al. [58] ^B^	16	13:3	41 ± 8	177 ± 5	74.6 ± 8	55.1 ± 5	345 ± 52 W	t ≥ 90% V̇O_2_max
Sousa et al. [59]	10	10:0	34.9 ± 8.4	175 ± 6.9	73.2 ± 9.5	51.9 ± 7.8	NR	t ≥ 90% V̇O_2_max
Balci et al. [60] ^B^	8	8:0	22.1 ± 3.1	175 ± 5.2	66.2 ± 8.5	64 ± 5.6	5.5 ± 0.3 W·kg^−1^	t ≥ 90% V̇O_2_max, t ≥ 95% V̇O_2_max
Bogdanis et al. [61] ^B^	11	11:0	28 ± 6	177± 7	70.4 ± 10.6	47.1± 6.6	242 ± 50 W	t ≥ 80% V̇O_2_max, t ≥ 85% V̇O_2_max, t ≥ 90% V̇O_2_max, t ≥ 95% V̇O_2_max
Duc et al. [62]	12	12:0	27 ± 9	182 ± 4	72.7 ± 5.3	72.5 ± 8	375 ± 58 W	t ≥ 90% V̇O_2_max
Järvinen et al. [63]	15	10:5	25.5 ± 3.3	178 ± 10.8	81.4 ± 10.5	38.9 ± 7.1	NR	t ≥ 90% V̇O_2_max
Wommer et al. [64]	10	10:0	27.6 ± 5	179 ± 5.8	80.2 ± 14.1	48.4 ± 8.1	327 ± 39 W	t ≥ 90% V̇O_2_max
Bossi et al. [65]	18	16:2	38 ± 11	177 ± 7	71.6 ± 7.9	54.3 ± 8.9	5.01 ± 0.8 W·kg^−1^	t ≥ 90% V̇O_2_max, t ≥ 95% V̇O_2_max
Bossi et al. [66] ^B^	13	13:0	25 ± 6	184 ± 5	75 ± 5	69.7 ± 7.1	431 ± 38 W	t ≥ 90% V̇O_2_max
Miller et al. [67]	8	7:1	26.8 ± 5.7	173 ± 7.7	75.4 ± 16.1	56.9 ± 6.6	NR	t ≥ 90% V̇O_2_max
Norouzi et al. [68]	10	10:0	22.8 ± 4.3	180 ± 7.5	72.9 ± 9.5	64.8 ± 5.1	382 ± 57.1 W	t ≥ 95% V̇O_2_max
Twist et al. [69]	11	11:0	20 ± 0.8	181 ± 12	82.3 ± 12	42.8 ± 5	251 ± 23 W	t ≥ 90% V̇O_2_max
Bossi et al. [70]	22	18:4	36 ± 12	178 ± 10	75.2 ± 14	51.6 ± 5.3	352 ± 55 W	t ≥ 90% V̇O_2_max,t ≥ 95% V̇O_2_max
Odden et al. [38]	22	19:3	22.6 ± 6	180 ± 8	71.9 ± 9.3	67.1 ± 6.4	5.8 ± 0.6 W·kg^−1^	t ≥ 90% V̇O_2_max
Urianstad et al. [71] [♀]	11	0:11	23.8 ± 5	172 ± 8.1	66.6 ± 9.5	62.5 ± 6.4	5.9 ± 1 W·kg^−1^	t ≥ 90% V̇O_2_max
[♂]	8	8:0	21 ± 1.4	179 ± 4.8	67.3 ± 3.6	81 ± 5.2	6.8 ± 0.4 W·kg^−1^	t ≥ 90% V̇O_2_max
Appelhans et al. [72]	12	12:0	24 ± 2	178 ± 5	73 ± 10	68 ± 6.8	358 ± 51 W	t ≥ 90% V̇O_2_max
Boynton et al. [73] [HIIT_13^⸰^]	10	7:3	33 ± 6	177 ± 10	71.5 ± 13.5	56.9 ± 11.2	352 ± 59 W	t ≥ 90% V̇O_2_max
[HIIT_35^⸰^]	10	7:3	36 ± 12	175 ± 8	70.1 ± 11.4	57.3 ± 9.5	346 ± 63 W	t ≥ 90% V̇O_2_max
Çabuk et al. [74]	12	12:0	20 ± 1	176 ± 5	65.9 ± 6	54 ± 6.2	294 ± 16 W	t ≥ 80% V̇O_2_max, t ≥ RCP
Raimundo et al. [75]	15	15:0	32 ± 5	176 ± 6	83 ± 13	3.05 ± 0.52	~ 306 W	t ≥ 90% V̇O_2_max, t ≥ 95% V̇O_2_max
Twist et al. [76]	16	16:0	20.9 ± 0.9	180 ± 7	75.7 ± 6.2	50.6 ± 6.2	301 ± 47 W	t ≥ 90% V̇O_2_max
**Jumping (and Running)**								
Ducrocq et al. [77]	13	9:4	21.2 ± 1.9	173 ± 9	67.4 ± 9.4	55 ± 7	14.4 ± 1.8 km·h^−1^	t ≥ 90% V̇O_2_max
**Kayaking**								
Paquette et al. [78]	13	8:5	22 ± 3	NR	71.5 ± 8.3	51.8 ± 7.7	122 ± 36 W	t ≥ 90% V̇O_2_max, t ≥ 95% V̇O_2_max
**Rowing**								
Patterson et al. [79]	8	0:8	20 ± 1.6	NR	71 ± 8.8	3.46 ± 0.64	NR	t ≥ 95% V̇O_2_max
Faelli et al. [80] ^C^	10	10:0	15.7 ± 0.7	180 ± 5.3	68.8 ± 8.2	60.1 ± 6.0	297 ± 39.4 W	t ≥ 90% V̇O_2_max
**Running**								
Billat et al. [34]	8	8:0	34 ± 6	175 ± 5	69 ± 4	59.8 ± 5.4	18.5 ± 1.2 km·h^−1^	t ≥ V̇O_2_max – 2.1, (≈ t ≥ 95% V̇O_2_max)
Demarie et al. [33] ^A^	15	12:3	44.6 ± 7	173 ± 7	67.6 ± 5	56.3 ± 4.4	16.6 ± 1.1 km·h^−1^	t ≥ V̇O_2_max
Billat et al. [81]	7	7:0	51 ± 6	175 ± 5	71 ± 4	52.1 ± 6	15.9 ± 1.8 km·h^−1^	t ≥ V̇O_2_max
Dupont et al. [82]	9	9:0	25 ± 2.8	179 ± 6	73.8 ± 7.9	54.1 ± 6.8	16.7 ± 1.3 km·h^−1^	t ≥ 90% V̇O_2_max, t ≥ 95% V̇O_2_max
Millet et al. [83]	8	UNS	22.6 ± 5	175 ± 3.9	65.9 ± 6.3	71.2 ± 4.2	19.9 ± 0.9 km·h^−1^	t ≥ 90% V̇O_2_max, t ≥ 95% V̇O_2_max
Millet et al. [84]	7	UNS	21.9 ± 2.9	174 ± 3.7	64.7 ± 5.8	71.1 ± 4.9	19.8 ± 0.9 km·h^−1^	t ≥ 90% V̇O_2_max
Dupont & Berthoin [85]	12	12:0	23.6 ± 3.7	177 ± 4.6	71.6 ± 8.3	57.8 ± 7.1	17.2 ± 1.8 km·h^−1^	t ≥ 90% V̇O_2_max, t ≥ 95% V̇O_2_max
Tardieu-Berger et al. [86] ^C^	11	11:0	16.6 ± 1.3	177 ± 6.6	63 ± 7.3	62.6 ± 3.6	17.7 ± 1.0 km·h^−1^	t ≥ 90% V̇O_2_max, t ≥ 95% V̇O_2_max
Midgley et al. [87]	22	16:6	38 ± 7.1	174 ± 7	70.1 ± 7.8	3.87 ± 0.47	16.2 ± 2 km·h^−1^	t ≥ 90% V̇O_2_max, t ≥ 95% V̇O_2_max
Midgley et al. [88]	9	9:0	38.2 ± 8.8	174 ± 7	74.5 ± 10.8	4.09 ± 0.54	16.3 ± 1.2 km·h^−1^	t ≥ 95% V̇O_2_max, t ≥ V̇O_2_max
Rozenek et al. [89]	12	12:0	20.4 ± 1.2	169 ± 4.8	70.7 ± 5.2	56.9 ± 4.3	16.3 ± 1.1 km·h^−1^	t ≥ 90% V̇O_2_max
Thevenet et al. [37], and Thevenet et al. [90]	8	8:0	15.9 ± 1.4	171 ± 4	57.7 ± 4.8	57.4 ± 6.1	17.9 ± 0.4 km·h^−1^	t ≥ 90% V̇O_2_max, t ≥ 95% V̇O_2_max
Thevenet et al. [91]	9	9:0	16.6 ± 1	158 ± 7	63.4 ± 7.8	64.9 ± 4.2	17.7 ± 0.9 km·h^−1^	t ≥ 90% V̇O_2_max, t ≥ 95% V̇O_2_max
O’Brien et al. [92]	17	14:3	21.9 ± 3.9	NR	74 ± 11.3	57.4 ± 8.7	NR	t ≥ 90% V̇O_2_max
Wakefield & Glaister [93] ^B^	7	7:0	22 ± 5	182 ± 5.6	86.4 ± 11.4	51.5 ± 5.1	NR	t ≥ 95% V̇O_2_max
Zafeiridis et al. [94]	9	9:0	14 ± 0.6	173 ± 6	60.5 ± 7.2	55.5 ± 4.6	15 ± 0.5 km·h^−1^	t ≥ 80% V̇O_2_max, t ≥ 85% V̇O_2_max, t ≥ 90% V̇O_2_max, t ≥ 95% V̇O_2_max
Zafeiridis et al. [95]^B^ [YA]	10	10:0	13.2 ± 0.3	159 ± 8	47.8 ± 7.1	57.9 ± 3.5	14.5 ± 1.4 km·h^−1^	t ≥ 80% V̇O_2_max, t ≥ 90% V̇O_2_max, t ≥ 95% V̇O_2_max
[MEN]	10	10:0	21 ± 1.6	175 ± 4	70.9 ± 4.9	58 ± 3.7	15.6 ± 1.2 km·h^−1^	t ≥ 80% V̇O_2_max, t ≥ 90% V̇O_2_max, t ≥ 95% V̇O_2_max
Buchheit et al. [96]	8	8:0	18.6 ± 5.3	173 ± 11.3	58.2 ± 12.1	62.4 ± 5.2	19.5 ± 0.8 km·h^−1^	t ≥ 90% V̇O_2_max
Abderrahman et al. [97] [G1 - PAS]	9	9:0	20.4 ± 0.6	181 ± 3.5	74.1 ± 8.8	58.7 ± 4.2	15.5 ± 1 km·h^−1^	t ≥ 90% V̇O_2_max, t ≥ 95% V̇O_2_max
[G2 – ACT]	9	9:0	20.9 ± 0.8	179 ± 3.3	76.8 ± 8.3	59.4 ± 7.5	16.1 ± 1.2 km·h^−1^	t ≥ 90% V̇O_2_max, t ≥ 95% V̇O_2_max
De Aguiar et al. [98]	7	7:0	22.4 ± 3.1	175 ± 5	77.2 ± 5.7	50.3 ± 3.5	14.2 ± 1.2 km·h^−1^	t ≥ 95% V̇O_2_max
Panissa et al. [99] [Moderate]	7	7:0	23 ± 5	181 ± 6	86.9 ± 6.2	45.3 ± 4	17.8 ± 1.9 km·h^−1^	t ≥ 90% V̇O_2_max
[High]	7	7:0	28 ± 6	180 ± 6	75.1 ± 7.7	60.2 ± 6.2	21.1 ± 1.6 km·h^−1^	t ≥ 90% V̇O_2_max
Panasci et al. [100]	15	15:0	22 ± 1	176 ± 6	66 ± 7	53 ± 0.6	18.6 ± 1 km·h^−1^	t ≥ 90% V̇O_2_max
Smilios et al. [101]	11	11:0	22.1 ± 1	178 ± 8	76.3 ± 8.4	52 ± 4	16 ± 1.5 km·h^−1^	t ≥ 80% V̇O_2_max, t ≥ 90% V̇O_2_max, t ≥ 95% V̇O_2_max
Baquet et al. [102]	11	5:6	9.6 ± 0.8	138 ± 7	37.7 ± 11.2	44.5 ± 6.2	10.4 ± 1 km·h^−1^	t ≥ 80% V̇O_2_max, t ≥ 90% V̇O_2_max
Schoenmakers & Reed [103]	12	12:0	34 ± 11	180 ± 6	74 ± 6	53 ± 7	NR	t ≥ 90% V̇O_2_max, t ≥ 95% V̇O_2_max
Ducrocq et al. [77]	13	9:4	21.2 ± 1.9	173 ± 9	67.4 ± 9.4	55 ± 7	14.4 ± 1.8 km·h^−1^	t ≥ 90% V̇O_2_max
Myrkos et al. [104]	12	12:0	22.1 ± 1	175 ± 6.7	70.5 ± 6.2	59.1 ± 4.9	16.7 ± 0.8 km·h^−1^	t ≥ 80% V̇O_2_max, t ≥ 90% V̇O_2_max, t ≥ 95% V̇O_2_max
Beltrami et al. [55]	15	12:3	30 ± 4	177 ± 10	68 ± 10	58 ± 4	17.1 ± 1.5 km·h^−1^	t ≥ 90% V̇O_2_max
José Arantes et al. [105] ^B,C^	11	11:0	25.5 ± 4.1	174 ± 9	64.9 ± 8.2	55.9 ± 1.4	18.2 ± 0.3 km·h^−1^	t ≥ 90% V̇O_2_max
Germano et al. [106] ^B^	8	8:0	28 ± 3.7	174 ± 3.7	75 ± 10.5	49.5 ± 5.9	14.9 ± 1.6 km·h^−1^	t ≥ 80% V̇O_2_max, t ≥ 85% V̇O_2_max, t ≥ 90% V̇O_2_max, t ≥ 95% V̇O_2_max
Sánchez-Otero et al. [107]	11	11:0	36.6 ± 6.9	175 ± 7.2	71.3 ± 10.3	59.3 ± 5.3	18.7 ± 1.1 km·h^−1^	t ≥ 90% V̇O_2_max
Bok et al.[108] ^C^	17	17:0	23.6 ± 1.1	180 ± 5.9	78.2 ± 8.1	54.1 ± 3	17.4 ± 0.9 km·h^−1^	t ≥ 90% V̇O_2_max
Held et al. [109] ^A^	17	9:8	25.8 ± 5	175 ± 7	63.2 ± 4.5	63.3 ± 4.5	NR	t ≥ 90% V̇O_2_max
Twist et al. [69]	11	11:0	20 ± 0.8	181 ± 12	82.3 ± 12	48.1 ± 6.5	13.2 ± 1.4 km·h^−1^	t ≥ 90% V̇O_2_max
Varela-Sanz et al. [110]	8	8:0	36.7 ± 7.1	177 ± 5.9	73.8 ± 8.4	58.2 ± 3.4	18.4 ± 0.1 km·h^−1^	t ≥ 90% V̇O_2_max
Vaccari et al. [111]	9	9:0	35 ± 11	NR	79 ± 8	52 ± 5	15.2 ± 1.6 km·h^−1^	t ≥ 90% V̇O_2_max
Fleckenstein et al. [112] ^A^	12	7:5	22.3 ± 2.3	NR	NR	59.1 ± 2.4	NR	t ≥ 90% V̇O_2_max, t ≥ 95% V̇O_2_max
**Swimming**								
Bentley et al.[113]	8	5:3	18.4 ± 1.8	179 ± 6.8	66.9 ± 6.5	55.7 ± 5.8	1.42 ± 0.06 m·s^−1^	t ≥ 90% V̇O_2_max
Libicz et al. [114] ^B^	10	10:0	22.8 ± 4.1	181 ± 7.9	72.3 ± 6.6	53 ± 6.7	1.22 ± 0.06 m·s^−1^	t ≥ 95% V̇O_2_max
Almeida et al. [115]	12	7:5	15.3 ± 1.4	169 ± 10	58.9 ± 9.8	59.2 ± 4.2	1.27 ± 0.09 m·s^−1^	t ≥ VT_2_, t ≥ 90% V̇O_2_max, t ≥ 95% V̇O_2_max
Almeida et al. [116]	22	13:9	16.1 ± 1.7	173 ± 10.2	65.5 ± 10.6	55.2 ± 5.6	1.26 ± 0.09 m·s^−1^	t ≥ VT_2_, t ≥ 90% V̇O_2_max
**Cross Country Skiing**								
Rønnestad et al. [117]	11	11:0	23.5 ± 3.5	184 ± 6	77.9 ± 7.2	70.6 ± 5.7	12.6 ± 0.9 km·h^−1^	t ≥ 90% V̇O_2_max
Rønnestad et al. [118]	10	10:0	25 ± 3	186 ± 4	81.1 ± 5	69.6 ± 3.5	16 ± 0.6 km·h^−1^	t ≥ 90% V̇O_2_max
Rønnestad et al. [119]	12	12:0	21.2 ± 2.9	182 ± 5	75.1 ± 4.1	69.6 ± 4.3	18.4 ± 1 km·h^−1^	t ≥ 90% V̇O_2_max

Abbreviations*: NR* Not reported, *RCP* Respiratory compensation point, *SD* Standard deviation, *t* Time, *UNS* Unspecified, *V̇O*_*2*_*max* Maximum oxygen uptake (at times V̇O_2_peak), *VT*_*2*_ Second ventilatory threshold

^A^ – Demographics calculated from available group data

^B^ – Data extracted from figure

^C^ – Data calculated from Standard Error to SD

All data are presented as mean ± SD

**Table 2:** HIIT protocol characteristics of included studies, and measures of tV̇O_2_max reported

Study [group identifier]	High-intensity interval training Protocol					Total Training Duration (s)	Work Interval Duration (s)	$\dot{\mathrm{V}}{\mathrm{O}_{2}}\mathrm{max}$ ≥ 5 min	$\dot{\mathrm{V}}{\mathrm{O}_{2}}\mathrm{max}$≥ 10 min
	Repetitions	Work Duration	Work Intensity	Recovery Duration	Recovery Intensity				
**Cycling**									
Zuniga et al. [41] ^A^	TTE	F, 30 s	90% MAP	F, 30 s	50% MAP	1530 ± 318	765 ± 159	✓ ^(+)^	
	TTE	F, 3 min	90% MAP	F, 3 min	50% MAP	1272 ± 408	636 ± 204		
	TTE	F, 30 s	MAP	F, 30 s	50% MAP	1146 ± 360	573 ± 180	✓ ^(+)^	
	TTE	F, 3 min	MAP	F, 3 min	50% MAP	834 ± 288	417 ± 144		
Nicolò et al. [42]	TTE	F, 30 s	135% MAP	F, 30 s	PAS	2280 ± 780	1140 ± 390		
	TTE	F, 40 s	135% MAP	F, 20 s	PAS	600 ± 180	402 ± 121	✓	
Lisbôa et al. [43]	TTE	I, 60% T_low_, 82.5 ± 8.8 s	CPO, I_high_	I, 60% T_low_, 82.5 ± 8.8 s	30% MAP	450 ± 66	225 ± 33		
	TTE	I, T_low_, 137.5 ± 14.6 s	DPO, I_high_	I, T_low_, 137.5 ± 14.6 s	30% MAP	742 ± 118	371 ± 89		
Zadow et al. [44] ^B^	3	F, 3 min	*‘All out’*	F, 3 min	50% PO_AT_	1080	540	✓ ^(-)^	
	3	F, 3 min	CPO *‘All out’*	F, 3 min	50% PO_AT_	1080	540	✓ ^(-)^	
	3	F, 3 min	A-CPO, *‘All out’*	F, 3 min	50% PO_AT_	1080	540	✓ ^(-)^	
Zafeiridis et al. [45]	TTE	F, 30 s	110% MAP	F, 30 s	PAS	1740 ± 150	870 ± 150		
	TTE	F, 2 min	95% MAP	F, 2 min	PAS	2160 ± 120	1080 ± 60	✓ ^(-)^	
Rønnestad & Hansen [46]	TTE	F, 30 s	MAP	F, 15 s	50% MAP	1385 ± 547	928 ± 365	✓	✓
	TTE	I, 50% T_max_, 170 ± 54 s	MAP	I, 25% T_max_, 85 ± 54 s	50% MAP	827 ± 285	554 ± 191	✓	
	TTE	I, 80% T_max_, 272 ± 86 s	MAP	I, 25% T_max_, 136 ± 43 s	50% MAP	642 ± 191	430 ± 128	✓	
Turnes et al. [39] [LOW]	4	F, 5 min	105% CP	F, 1 min	PAS	1440	1200		
	7	F, 5 min	105% CP	F, 1 min	PAS	2520	2100		
[UP]	2 * 4	F, 80 s	I_high_	F, 160 s (/10 min between sets)	PAS	1920 (/2520)	640		
	2 * 7	F, 80 s	I_high_	F, 160 s (/10 min between sets)	PAS	3360 (/3960)	1120		
Combes et al. [47]	30	F, 1 min	70% MAP	F, 1 min	PAS	3600	1800	✓	
Rønnestad et al. [48]	6	F, 5 min	Isoeffort Max	F, 2.5 min	50% PO_Interval_	2700	1800	✓	
	6	F, 5 min	Isoeffort Max (+ vibration)	F, 2.5 min	50% PO_Interval_	2700	1800	✓	✓
Shi et al. [49]	40	F, 6 s	Max	F, 15 s	PAS	840	240	✓	
	40	F, 6 s	Max	F, 30 s	PAS	1440	240		
	40	F, 6 s	Max	F, 1 min	PAS	2640	240		
	8	F, 30 s	Max	F, 4 min	PAS	2160	240		
Boynton et al. [50] [5⁰], [13⁰], [22⁰], and [35⁰]	4	F, 5 min	Isoeffort Max	F, 5 min	PAS, then ’volition’, then 50% MAP	2700	1200	1 x ✓	
Lisbôa et al. [51]	TTE	I, 60% T_low_, 74 ± 16 s	CPO, P_high_	I, 60% T_low_, 74 ± 16 s	30% MAP	548 ± 32	274 ± 16		
	TTE	I, T_low_, 124 ± 27 s	DPO, P_high > 105% CP_	I, T_low_, 124 ± 27 s	30% MAP	1265 ± 446	632 ± 223	✓ ^(+)^	
Almquist et al. [52] ^B^	3 * 13	F, 30 s	MAX	F, 15 s (/NR between sets)	50% PO_Interval_	1755 (/NR)	1170	✓	✓
	4	F, 5 min	Isoeffort Max	F, 2.5 min	50% PO_Interval_	1800	1200	✓	
Bossi et al. [31]	6	F, 5 min	CPO, 84% MAP	F, 2.5 min	30% MAP	2700	1800		
	6	F, 5 min	VPO, 84% MAP	F, 2.5 min	30% MAP	2700	1800	✓	
Vaccari et al. [53]	TTE	F, 30 s	117% CP	F, 20 s	83% CP	714 ± 265	478 ± 178		
	TTE	F, 3 min	117% CP	F, 2 min	83% CP	664 ± 282	445 ± 189		
	TTE	F, 3-, 2-, 1 min, 40-, 30 s	117% CP	F, 2 min, 80-, 40-, 30-, 20 s	83% CP	798 ± 185	535 ± 124	✓	
Dall’Agnol et al. [54]	4	F, 4 min	Isoeffort Max	F, 1 min	~90 W	1200	960		
	4	F, 4 min	Isoeffort Max	F, 2 min	~90 W	1440	960	✓	
	4	F, 8 min	Isoeffort Max	F, 2 min	~90 W	2400	1920	✓	
	4	F, 8 min	Isoeffort Max	F, 4 min	~90 W	2880	1920	✓	✓
Beltrami et al. [55]	4	F, 4 min	CPO, 85% MAP	F, 3 min	ACT	1680	960	✓	
	4	F, 4 min	DPO, 85% MAP	F, 3 min	ACT	1680	960	✓	
Fennell & Hopker [56]	6	F, 4 min	Isoeffort Max	F, 2 min	PAS	2160	1440	✓	✓
	6	F, 4 min	Isoeffort Max	F, 2 min	80% PO_LT_	2160	1440	✓	✓
	6	F, 4 min	Isoeffort Max	F, 2 min	110% PO_LT_	2160	1440	✓	✓
	3	F, 8 min	Isoeffort Max	F, 4 min	PAS	2160	1440	✓	✓
	3	F, 8 min	Isoeffort Max	F, 4 min	80% PO_LT_	2160	1440	✓	✓
	3	F, 8 min	Isoeffort Max	F, 4 min	110% PO_LT_	2160	1440	✓	✓
Fennell & Hopker [29]	6	F, 4 min	Isoeffort Max	F, 2 min	PAS	2160	1440	✓	✓
	6	F, 4 min	Isoeffort Max	I, mVO_2_ Recovery, 205 ± 79 s	PAS	2670 ± 438	1440	✓	✓
	3	F, 8 min	Isoeffort Max	F, 4 min	PAS	2160	1440	✓	✓
	3	F, 8 min	Isoeffort Max	I, mVO_2_ Recovery, 200 ± 81 s	PAS	2040 ± 243	1440	✓	✓
Ksoll et al. [57]	30	F, 30 s	80% MAP	F, 30 s	85% GET	1800	900		
	5	F, 3 min	80% MAP	F, 3 min	85% GET	1800	900	✓ ^(-)^	
McKee et al. [58] ^B^ [Intervals commenced at 1) Beginning, 2) Middle, or 3) End of the session]	3	F, 3 min	90% MAP	F, 3 min	40% PO_VT_	1080	540		
Sousa et al. [59]	2 * 6	F, 10 s	MAX	F, 20 s (/3 min between sets)	ACT	360 (/540)	120	In %’s	
	6	F, 1 min	90% Δ (PO_VT –_ MAP)	F, 1 min	50% MAP	720	360	In %’s	
Balci et al. [60] ^B^	16	F, 45 s	110% MAP	F, 45 s	35% MAP	1440	720	✓	
	3	F, 3 min	95% MAP	F, 3 min	35% MAP	1440	720	✓	
	6	F, 30 s	MAX	F, 3.5 min	35% MAP	1440	180		
Bogdanis et al. [61] ^B^	48	F, 10 s	MAP	F, 15 s	15% MAP	1200	480		
	16	F, 30 s	MAP	F, 45 s	15% MAP	1200	480		
	8	F, 1 min	MAP	F, 1.5 min	15% MAP	1200	480		
Duc et al. [62]	6	F, 5 min	VPO, 90% MAP	F, 2.5 min	30 - 40% MAP	2700	1800	✓	
	6	F, 5 min	VPO, 90% MAP (+ vibration)	F, 2.5 min	30 - 40% MAP	2700	1800	✓	✓
Järvinen et al. [63]	2 * 10	F, 30 s	MAP	F, 30 s (/4 min between sets)	50% MAP	1200 (/1440)	600	✓	✓
Wommer et al. [64]	8	F, 1 min	CPO, MAP	F, 1 min	80% PO_LT_	960	480		
	8	F, 1 min	DPO, MAP	F, 1 min	80% PO_LT_	960	480	✓	
	8	F, 1 min	IPO, MAP	F, 1 min	80% PO_LT_	960	480	✓	
Bossi et al. [65]	TTE	F, 4 min	70% Δ	F, 2 min	20% PO_Interval_	1835 ± 947	1262 ± 623	✓	
	TTE	F, 4 min	85% MAP	F, 2 min	20% PO_Interval_	1423 ± 840	990 ± 549	✓	
	TTE	F, 4 min	120% PO_20k TT_	F, 2 min	20% PO_Interval_	1007 ± 803	715 ± 524	✓	
	TTE	F, 4 min	80% W'	F, 2 min	20% PO_Interval_	1087 ± 848	767 ± 554	✓	
Bossi et al. [66] ^B^	6	F, 5 min	VPO, 84% MAP (+ vibration)	F, 2.5 min	30% MAP	2700	1800	✓	
Miller et al. [67]	3	F, 4 min	CPO, 60% W'	F, 3 min	ACT, 50 W	1440	720	In %’s	
	3	F, 4 min	DPO, 60% W'	F, 3 min	ACT, 50 W	1440	720	In %’s	
Norouzi et al. [68]	16 - 18	F, 1 min	105% MAP	F, 1 min	30% MAP	1992 ± 151	996 ± 76		
	4 - 7	I, 50% T_max_, 89 ± 19 s	I_high_	I, T_max,_ 178 ± 38 s	30% MAP	1433 ± 215	539 ± 84		
	4 - 6	F, 3 min	95% MAP	F, 3 min	30% MAP	1681 ± 346	841 ± 173		
Twist et al. [69]	3 * 12	F, 15 s	120% MAP	F, 15 s (/5 min between sets)	PAS	1080 (/1680)	540		
Bossi et al. [70]	TTE	F, 4 min	70% Δ (W_GET_ + 70% W_GET_ – MAP)	F, 2 min	20% PO_Interval_	1099 ± 503	777 ± 332	✓	
	TTE	F, 4 min	70% Δ (W_GET_ + 70% W_GET_ – MAP)	F, 2 min	20% PO_Interval_	1262 ± 502	886 ± 337	✓	
	TTE	F, 4 min	70% Δ (W_GET_ + 70% W_GET_ – MAP)	F, 2 min	20% PO_Interval_	1240 ± 784	869 ± 516	✓	
	TTE	F, 4 min	70% Δ (W_GET_ + 70% W_GET_ – MAP)	F, 2 min	20% PO_Interval_	1274 ± 639	891 ± 425	✓	
Odden et al. [38]	5	F, 8 min	CPO, PO_40kmTT_	F, 3 min	35% PO_40kmTT_	3300	2400	✓	✓
	5	F, 8 min	VPO, PO_40kmTT_	F, 3 min	90% PO_40kmTT_	3300	2400	✓	
	5 * 11	F, 30 s	118% PO_40kmTT_	F, 15 s (/3 min between sets)	60% PO_40kmTT_	3300	2400	✓	
Urianstad et al. [71] [♀ and ♂]	6	F, 8 min	CPO, PO_40kmTT_	F, 3 min	35% PO_40kmTT_	3960	2880	2 x ✓	2 x ✓
	6	F, 8 min	VPO, PO_40kmTT_	F, 3 min	35% PO_40kmTT_	3960	2880	2 x ✓	2 x ✓
	6 * 11	F, 30 s	118% PO_40kmTT_	F, 15 s (/3 min between sets)	60% PO_40kmTT_	3960	2880	2 x ✓	2 x ✓
Appelhans et al. [72]	6	F, 5 min	84% MAP	F, 2.5 min	60% MAP ^B^	2505	1755		
	6	F, 5 min	Isoeffort Max	F, 2.5 min	60% MAP ^B^	2505	1755	✓	✓
	3 * 13	F, 30 s	MAP	F, 15 s (/6:15 between sets)	50% MAP	1755 (/2505)	1170	✓	
	3 * 13	F, 30 s	Isoeffort Max	F, 15 s (/6:15 between sets)	50% MAP	1755 (/2505)	1170	✓	✓
Boynton et al. [73] [HIIT_13 and HIIT_35]	5	F, 4 min	Isoeffort Max	F, 5 min	PAS, then ’volition’, then 50% MAP	2700	1200	2 x ✓	1 x ✓
Çabuk et al. [74]	4 * 3	F, 10 s	MAX	F, 10 s (/3:40 between sets)	Unloaded cycling	720	120		
	4 * 2	F, 15 s	MAX	F, 15 s (/3:45 between sets)	Unloaded cycling	720	120		
	4	F, 30 s	MAX	F, 4 min	Unloaded cycling	720	120		
Raimundo et al. [75]	10	F, 1 min	85% MAP	F, 30 s	PAS	900	600		
	5	F, 2 min	85% MAP	F, 1 min	PAS	900	600		
Twist et al. [76]	2 * 12	F, 15 s	120% MAP	F, 15 s (/5 min between sets)	PAS	720 (/1020)	360		
	2 * 6	F, 30 s	120% MAP	F, 30 s (/5 min between sets)	PAS	720 (/1020)	360		
**Jumping**									
Ducrocq et al. [77]	22	F, 15 s	7 Drop jumps	F, 15 s	PAS	660	330		
	22	F, 15 s	9 Drop jumps	F, 15 s	PAS	660	330		
**Kayaking**									
Paquette et al. [78]	2 * 20	F, 15 s	110% MAP	F, 15 s (/5 min between sets)	30% MAP	1200 (/1500)	600	✓	
	2 * 10	F, 30 s	110% MAP	F, 30 s (/5 min between sets)	30% MAP	1200 (/1500)	600	✓	
	6	F, 30 s	Max	F, 3.5 min	*Choice*	1440	180		
	6	F, 1 min	130% MAP	F, 3 min	*Choice*	1440	360		
**Rowing**									
Patterson et al. [79]	TTE	F, 30 s	MAP	F, 15 s	PAS	1857 ± 204	1242 ± 150		
	TTE	F, 1 min	MAP	F, 30 s	ACT	2604 ± 729	1734 ± 540		
	TTE	F, 1 min	MAP	F, 1 min	ACT	4992 ± 1632	2496 ± 816		
	TTE	F, 2 min	MAP	F, 1 min	ACT	1752 ± 718	1152 ± 474		
	TTE	F, 2 min	MAP	F, 2 min	ACT	3108 ± 1260	1554 ± 630		
	TTE	F, 3 min	MAP	F, 1.5 min	ACT	1596 ± 495	1056 ± 324		
	TTE	F, 3 min	MAP	F, 3 min	ACT	2640 ± 1020	1320 ± 510	✓ ^(+)^	
Faelli et al. [80] ^C^	25	F, 30 s	MAP	F, 30 s	20% MAP	1500	750	✓	
	4	F, 4 min	90% MAP	F, 3 min	30% MAP	1680	960	✓	✓
**Running**									
Billat et al. [34]	TTE	F, 30 s	vVO_2_max	F, 30 s	50% vVO_2_max	1140 ± 360	570 ± 180	✓ ^(+)^	
Demarie et al. [33] ^A^	TTE	I, 50% T_max_, 312 ± 43 s	50% Δ (V_LT_ + vVO_2max_)	I, 25% T_max_, 156 ± 21 s	25% Δ (V_LT_ + vVO_2max_)	1766 ± 465	1178 ± 310	✓ ^(+)^	✓ ^(+)^
Billat et al. [81]	TTE	F, 15 s	90% vVO_2_max	F, 15 s	80% vVO_2_max	1260 ± 360	630 ± 180	✓ ^(+)^	✓ ^(+)^
	TTE	F, 15 s	vVO_2_max	F, 15 s	70% vVO_2_max	1080 ± 300	540 ± 150	✓ ^(+)^	✓ ^(+)^
	TTE	F, 15 s	110% vVO_2_max	F, 15 s	60% vVO_2_max	540 ± 180	270 ± 90	✓ ^(+)^	
Dupont et al.[82]	TTE	F, 15 s	110% MAV	F, 15 s	PAS	1396 ± 710	698 ± 355	✓	
	TTE	F, 15 s	120% MAV	F, 15 s	PAS	694 ± 468	347 ± 234	✓	
	TTE	F, 15 s	130% MAV	F, 15 s	PAS	334 ± 140	167 ± 70		
	TTE	F, 15 s	140% MAV	F, 15 s	PAS	200 ± 100	100 ± 50		
Millet et al. [83]	3 * 7.8	F, 30 s	vVO_2_max	F, 30 s (/5 min between sets)	50% vVO_2_max	1414 ± 294 (/2014)	707 ± 147		
	3 * 3.9	F, 1 min	vVO_2_max	F, 30 s (/5 min between sets)	50% vVO_2_max	1058 ± 196 (/1658 ± 294)	707 ± 147	✓	
	3 * 2	I, 50% T_max_, 117.8 ± 25 s	vVO_2_max	I, 50% T_max_, 117.8 ± 25 s (/5 min between sets)	50% vVO_2_max	1414 ± 294 (/2014 ± 294)	707 ± 147	✓	
Millet et al. [84]	3 * 8	F, 30 s	vVO_2_max	F, 30 s (/5 min between sets)	50% vVO_2_max	1451 ± 237 (/2051 ± 237)	731 ± 119		
	3 * 8	F, 30 s	105% vVO_2_max	F, 30 s (/5 min between sets)	50% vVO_2_max	1451 ± 237 (/2051 ± 237)	731 ± 119	✓	
Dupont & Berthoin [85]	TTE	F, 15 s	120% MAV	F, 15 s	50% MAV	445 ± 79	228 ± 40		
	TTE	F, 15 s	120% MAV	F, 15 s	PAS	745 ± 171	378 ± 86	✓	
Tardieu-Berger et al. [86] ^C^	TTE	F, 30 s	110% MAV	F, 30 s	50% MAV	622 ± 186	311 ± 93	✓	
	TTE, n * 6	F, 30 s	110% MAV	F, 30 s (/4 min between sets)	50% MAV	960 ± 338 (/1440 ± 338)	480 ± 169		
Midgley et al. [87]	TTE	F, 30 s	105% vVO_2_max	F, 30 s	60% vVO_2_max	831 ± 238	426 ± 119	✓	
	TTE	F, 30 s	105% vVO_2_max	F, 30 s	60% vVO_2_max	898 ± 333	462 ± 168	✓	
Midgley et al. [88]	TTE	F, 30 s	105% vVO_2_max	F, 30 s	60% vVO_2_max	1110 ± 468	555 ± 234	✓ ^(+)^	
Rozenek et al. [89]	TTE	F, 15 s	vVO_2_max	F, 15 s	50% vVO_2_max	1059 ± 35	530 ± 17		
	TTE	F, 30 s	vVO_2_max	F, 15 s	50% vVO_2_max	771 ± 36	531 ± 18		
	TTE	F, 1 min	vVO_2_max	F, 15 s	50% vVO_2_max	621 ± 30	516 ± 15	✓	
Thevenet et al. [37], and Thevenet et al. [90]	TTE	F, 30 s	105% MAV	F, 30 s	50% MAV	1072 ± 388	536 ± 194	✓	✓
	TTE	F, 30 s	105% MAV	F, 30 s	67% MAV	705 ± 320	357 ± 178	✓	
	TTE	F, 30 s	105% MAV	F, 30 s	84% MAV	394 ± 81	197 ± 40	✓	
	TTE	F, 30 s	105% MAV	F, 30 s	PAS	2145 ± 829	1073 ± 415	✓	
Thevenet et al. [91]	TTE	F, 30 s	MAV	F, 30 s	50% MAV	1440 ± 169	720 ± 84	✓	
	TTE	F, 30 s	110% MAV	F, 30 s	50% MAV	653 ± 184	326 ± 92		
O’Brien et al. [92]	10	F, 1 min	MAV	F, 1 min	50% MAV	1200	600		
	5	F, 2 min	MAV	F, 2 min	50% MAV	1200	600		
Wakefield & Glaister [93] ^B^	TTE	F, 20 s	105% vVO_2_max	F, 20 s	PAS	3294 ± 426	1640 ± 220		
	TTE	F, 25 s	105% vVO_2_max	F, 20 s	PAS	3246 ± 300	1825 ± 175		
	TTE	F, 30 s	105% vVO_2_max	F, 20 s	PAS	2622 ± 624	1590 ± 360		
	TTE	F, 20 s	115% vVO_2_max	F, 20 s	PAS	2346 ± 684	1180 ± 340		
	TTE	F, 25 s	115% vVO_2_max	F, 20 s	PAS	1740 ± 390	975 ± 225		
	TTE	F, 30 s	115% vVO_2_max	F, 20 s	PAS	1188 ± 324	720 ± 180		
Zafeiridis et al. [94]	TTE	F, 30 s	110% MAV	F, 30 s	50% MAV	930 ± 462	480 ± 231		
	TTE	F, 3 min	95% MAV	F, 3 min	35% MAV	1700 ± 504	936 ± 270	✓	
Zafeiridis et al. [95] ^B^ [YA – SI]	TTE	F, 30 s	110% MAV	F, 30 s	50% MAV	708 ± 154	381 ± 65	✓	
[YA – LI]	TTE	F, 3 min	95% MAV	F, 3 min	35% MAV	1579 ± 554	882 ± 274	✓	✓
[Men – SI]	TTE	F, 30 s	110% MAV	F, 30 s	50% MAV	1087 ± 215	519 ± 155	✓	
[Men – LI]	TTE	F, 3 min	95% MAV	F, 3 min	35% MAV	1590 ± 349	882 ± 179	✓	
Buchheit et al. [96]	5	F, 3 min	90% vVO_2_max	F, 1.5 min	NR, *Rest*	1350	900	✓	
Abderrahman et al. [97] [G1 – PAS, Pre]	TTE	F, 30 s	105% MAV	F, 30 s	PAS	1883 ± 788	942 ± 394	✓	
[G1 – PAS, Post]	TTE	F, 30 s	105% MAV	F, 30 s	PAS	3015 ± 1095	1508 ± 548	✓	
[G2 – ACT, Pre]	TTE	F, 30 s	105% MAV	F, 30 s	50% MAV	923 ± 188	462 ± 194	✓	
[G2 – ACT, Post]	TTE	F, 30 s	105% MAV	F, 30 s	50% MAV	1163 ± 118	582 ± 59	✓	✓
De Aguiar et al. [98]	TTE	F, 30 s	CV, 105% ICV	F, 15 s	PAS	2159 ± 689	1439 ± 518		
	TTE	F, 30 s	CV, 125% ICV	F, 15 s	PAS	426 ± 101	284 ± 76		
	TTE	F, 30 s	DV, 125–105% ICV	F, 15 s	PAS	1576 ± 203	1051 ± 153		
Panissa et al. [99] [Moderate]	~13	F, 1 min	MAV	F, 1 min	PAS	1618 ± 174	809 ± 87	In %’s	
[High]	~11	F, 1 min	MAV	F, 1 min	PAS	1365 ± 104	682 ± 52	In %’s	
Panasci et al. [100]	15	F, 30 s	MAV (track)	F, 30 s	PAS	900	450	In %’s	
	15	F, 30 s	MAV (treadmill)	F, 30 s	PAS	900	450	In %’s	
	15	F, 30 s	115% MAV	F, 30 s	PAS	900	450		
Smilios et al. [101]	4	F, 4 min	90% MAV	F, 2 min	35% MAV	1440	960	✓	
	4	F, 4 min	90% MAV	F, 3 min	35% MAV	1680	960	✓	
	4	F, 4 min	90% MAV	F, 4 min	35% MAV	1920	960	✓	
Baquet et al. [102]	TTE	F, 15 s	120% MAV	F, 15 s	50% MAV	402 ± 184	201 ± 92		
	TTE	F, 15 s	120% MAV	F, 15 s	PAS	1244 ± 430	622 ± 215		
Schoenmakers & Reed [103]	6	F, 4 min	Isoeffort Max	F, 1 min	*Free to select either walking/standing*	1800	1440	✓	✓
	6	F, 4 min	Isoeffort Max	F, 2 min	*Free to select either walking/standing*	2160	1440	✓	✓
	6	F, 4 min	Isoeffort Max	F, 3 min	*Free to select either walking/standing*	2520	1440	✓	✓
	6	F, 4 min	Isoeffort Max	I, Perceived Readiness, 100 ± 34 s	*Free to select either walking/standing*	2040 ± 204	1440	✓	✓
Ducrocq et al. [77]	22	F, 15 s	120% MAV	F, 15 s	PAS	660	330		
Myrkos et al. [104]	TTE	F, 1 min	MAV	F, 30 s	PAS	1065 ± 332	713 ± 222	✓	
	TTE	F, 1 min	MAV	F, 1 min	PAS	1823 ± 482	912 ± 241	✓	
	TTE	F, 2 min	MAV	F, 1 min	PAS	844 ± 119	565 ± 80	✓	
	TTE	F, 2 min	MAV	F, 2 min	PAS	1197 ± 311	599 ± 156	✓	
Beltrami et al. [55]	4	F, 4 min	CV, 85% MAV	F, 3 min	ACT	1680	960	✓	✓
	4	F, 4 min	DV, 85% MAV	F, 3 min	ACT	1680	960	✓	✓
José Arantes et al. [105] ^B, C^	TTE	F, 30 s	110% MAV	F, 30 s	PAS	1463 ± 286	737 ± 139		
	TTE	F, 40 s	110% MAV	F, 20 s	PAS	416 ± 100	281 ± 66		
	TTE	F, 40 s	110% MAV	F, 40 s	PAS	1043 ± 230	526 ± 115		
Germano et al. [106] ^B^	5	I, T_max_, 120 ± 19 s	vVO_2_max	F, 2 min	V_VT_	1200 ± 174	600 ± 87	✓	
	5	I, T_max_, 155 ± 20 s	vVO_2_max	F, 2 min	PAS	1375 ± 176	775 ± 99	✓	
	5	I, T_max_, 106 ± 24 s	vVO_2_max	F, 8 min	V_VT_	2930 ± 663	530 ± 120	✓	✓
	5	I, T_max_, 188 ± 44 s	vVO_2_max	F, 8 min	PAS	3338 ± 772	938 ± 217	✓	✓
Sánchez-Otero et al. [107]	4	F, 2 min	MAV	F, 2 min	80% V_LT_	960	480		
	4	F, 2 min	MAV	F, 2 min	PAS	960	480		
Bok et al. [108] ^B^	20	F, 15 s	110% vVO_2_max	F, 15 S	PAS	600	300		
	20	F, 15 s	Δ15% ASR	F, 15 S	PAS	600	300		
	20	F, 15 s	Δ25% ASR	F, 15 S	PAS	600	300		
Held et al. [109] ^A^	4	F, 5 min	Isoeffort Max	F, 90 s	PAS	1560	1200	✓	
	4	F, 5 min	Isoeffort Max (8% incline)	F, 90 s	PAS	1560	1200	✓	
Twist et al. [69]	3 * 12	F, 15 s	120% vVO_2_max	F, 15 s (/5 min between sets)	PAS	1080 (/1680)	540		
Varela-Sanz et al. [110]	TTE	F, 2 min	MAV	F, 2 min	80% V_VT_	2670 ± 916	1395 ± 458	✓	✓
	TTE	F, 2 min	MAV	F, 2 min	PAS	3210 ± 764	1665 ± 382	✓	✓
Vaccari et al. [111]	TTE	F, 30 s	CV_6_, ~ vVO_2_max	F, 20 s	66% CV	678 ± 116	407 ± 70		
	TTE	F, 3 min	CV_6_, ~ vVO_2_max	F, 2 min	66% CV	673 ± 115	404 ± 69	✓	
	TTE	F, 3-, 2-, 1 min, 40-, 30 s	CV_6_, ~ vVO_2_max	F, 2 min, 80-, 40-, 30-, 20 s	66% CV	998 ± 129	599 ± 77	✓	
Fleckenstein et al. [112] ^A^	24	F, 30 s	vVO_2_max	F, 30 s	55% vVO_2_max	1440	720		
	4	F, 3 min	95% vVO_2_max	F, 3 min	50% vVO_2_max	1440	720	✓	
**Swimming**									
Bentley et al. [113]	16	F, 100 m, 75 ± 3 s	Δ25% _(Velocity difference between_ V_VT and vVO2max)_	F, NR, ‘15 s’	NR, likely PAS	1434 ± 64	1194 ± 54	✓	
	4	F, 400 m, 303 ± 11 s	Δ25% _(Velocity difference between_ V_VT and vVO2max)_	F, NR, ’75 s’	NR, likely PAS	1512 ± 55	1212 ± 44	✓	
Libicz et al. [114] ^B^	16	F, 50 m, 40 ± 2 s	vVO_2_max	F, 15 s	NR, likely PAS	880 ± 47	640 ± 36		
	8	F, 100 m, 81 ± 4 s	vVO_2_max	F, 30 s	NR, likely PAS	890 ± 52	650 ± 37		
Almeida et al. [115]	8	F, 100 m, 78 ± 5 s	MAV	F, 15 s	NR, likely PAS	750 ± 60	630 ± 45		
	4	F, 200 m, 157 ± 4 s	MAV	F, 30 s	NR, likely PAS	750 ± 60	630 ± 45		
Almeida et al. [116]	TTE	F, 100 m, 79 ± 6 s	MAV	F, 15 s	PAS	1206 ± 591	1014 ± 497	✓	
**Cross Country Skiing**									
Rønnestad et al. [117]	5	F, 5 min	CV, 90% MAV	F, 3 min	ACT	2400	1500	✓	✓
	5	F, 5 min	DV, 90% MAV	F, 3 min	ACT	2400	1500	✓	✓
Rønnestad et al. [118]	5	F, 5 min	CV, 90% MAV (8% incline)	F, 3 min	V_LT + (20% of difference_ V_LT – MAV)_	2400	1500	✓	✓
	5	F, 5 min	DV, 90% MAV (8% incline)	F, 3 min	V_LT + (20% of difference_ V_LT – MAV)_	2400	1500	✓	✓
	5	F, 5 min	VV, 90% MAV (8% incline)	F, 3 min	V_LT + (20% of difference_ V_LT – MAV)_	2400	1500	✓	✓
Rønnestad et al. [119] [First – Second - Last]	6	F, 5 min	VV, 90% MAV (7% incline)	F, 3 min	PAS, then ACT	2880	1800	3 x ✓	3 x ✓

*Abbreviations*: *A-CPO* Athlete controlled constant power output, *ACT* Active recovery, *ASR* Anaerobic speed reserve, *CP* Critical power, *CPO* Constant power output, *CV* Critical velocity, *DPO* Decreasing power output, *DV* Decreasing velocity, *F* Fixed duration, *GET* Gas exchange threshold, *I* Individualised duration, *ICV* Intensity at critical velocity, *IPO* Increasing power output,* I*_*high*_ and *P*_*high*_ Highest intensity at which V̇O_2_max is still achieved, *MAP* Maximal aerobic power, *MAV* Maximal aerobic velocity, *Max* Maximal effort, *mVO*_*2*_ Muscle O_2_ recovery benchmark, *NR* Not reported, *PAS* Passive recovery, *PO*_*AT*_ Power output at aerobic threshold, *PO*_*Interval*_ Power output of work interval, *PO*_*LT*_ Power output at lactate threshold, *PO*_*VT*_ Power output at ventilatory threshold, *PO*_*20/40kmTT*_ Power output from time trial performance, *T*_*low*_ and *T*_*max*_ Time to exhaustion at maximal exercise intensities, *TTE* Time to exhaustion, *V*_*LT*_ Velocity at lactate threshold, *V*_*VT*_ Velocity at ventilatory threshold, V̇*O₂max* Maximum oxygen uptake, *VPO* Variable power output, *VV* Variable velocity, *v*V̇*O₂max* – Velocity at V̇O₂max OR Lowest velocity that elicits V̇O₂max

^A^ – Demographics calculated from available group data

^B^ – Data extracted from figure

^C^ – Data calculated from Standard Error to SD

Note: All data are presented as mean ± SD,

✓ = time ≥ 90% V̇O_2_max exceeds 5 min, or 10 min

✓^(+)^ = time ≥ 95% or ≥ V̇O_2_max exceeds 5 min, or 10 min (time ≥ 90% V̇O_2_max not reported)

✓ ^(-)^ = time ≥ 80% or ≥ 85% V̇O_2_max exceeds 5 min, or 10 min (time ≥ 90% V̇O_2_max not reported)

### Study quality and risk of bias assessment

The quality of included studies was assessed using the Physiotherapy Evidence Database scale (PEDro scale), of which items 5, 6, and 7 were omitted due to the inherent difficulty of blinding researchers, assessors, and participants in exercise interventions. Items 2 and 10, concerning between-group randomisation and statistical comparison, respectively, were interpreted in the context of within-group study designs. These modifications reduced the maximum attainable PEDro score to 7. Using this adjusted scale, ratings of 6 and 7 were classified as “excellent quality”, 5 as “good quality”, 4 as “moderate quality”, and 0 to 3 as “poor quality” [121].

Bias in the included studies was evaluated using the Cochrane Risk of Bias Tool for Randomized Crossover Trials (RoB-2). The risk of bias was rated as *‘low’, ‘some concerns’*, or *‘high’* across domains such as randomisation processes, carryover effects, and the selection and reporting of tV̇O_2_max [122]. Graphical summaries of the RoB-2 results were created using robvis software [123].

### Statistical analysis

Results are presented as mean ± SD. Time ≥ 90% and ≥ 95% V̇O_2_max were prioritised for quantitative analyses as the most frequently reported variables, while time ≥ 80%, ≥ 85% and 100% V̇O_2_max were included for descriptive comparison. Analysis of covariance (ANCOVA), with cumulative HIIT protocol duration as a covariate and sample size as a weighting factor, were used to compare time ≥ 90% and ≥ 95% V̇O_2_max across SI-HIIT, MI-HIIT and LI-HIIT protocols [10]. Tukey's post hoc testing was performed to identify pairwise differences between HIIT categories. Time to exhaustion during exhaustive HIIT protocols was compared between studies incorporating active versus passive recovery intervals, using repeated measures analysis of variance (ANOVA). These statistical analyses were run in JASP (Version 0.17.2.1), with significance levels set at *p* ≤.05.

A linear mixed-effects modelling approach was employed to examine associations between cumulative HIIT protocol duration and time ≥ 90% and ≥ 95% V̇O_2_max. For protocols involving exercise to exhaustion, cumulative HIIT protocol duration was averaged across individual study participants. Similarly, time ≥ 90% and ≥ 95% V̇O_2_max were averaged across participants within each trial. Random intercepts were specified to account for clustering of observations within studies, and observations were weighted by sample size to reflect the precision of the group-level estimates. For between-participant designs, each intervention arm was treated as a separate study [38, 39, 71, 73, 124]. Model building proceeded sequentially. First, a base model with cumulative HIIT protocol duration as a fixed effect was fitted. The potential contribution of exercise modality (i.e. running, cycling, kayaking, rowing, swimming, and cross-country skiing) was then tested by comparing the base model to a model including modality. Next, the effect of work interval duration was evaluated by comparing a model including cumulative HIIT protocol duration and work interval duration as a categorical variable. Finally, a potential interaction between these variables was assessed by comparing an additive model to one including the interaction term. Model comparisons were conducted using likelihood ratio tests. The adjusted intraclass correlation coefficient (ICC) and the marginal and conditional coefficients of determination (R^2^) were calculated. These analyses were performed in R (Version 4.4.1), with significance levels set at *p* ≤.05.

Meta-analyses were conducted to calculate standardised mean differences (SMD) alongside two-sided 95% CIs for tV̇O_2_max across different HIIT protocols. *Within-study* comparisons assessed tV̇O_2_max between 1) SI-HIIT, MI-HIIT, and LI-HIIT protocols, 2) passive versus active recovery intervals, and 3) even-paced versus variable-intensity work intervals. When reported, time ≥ 90% V̇O₂max was used as the outcome measure. If this metric was not available, the closest alternative threshold (e.g., time ≥ 85% or ≥ 95% V̇O₂max) was used (see Table 2). An inverse variance random-effects model was used to account for differences in sample sizes and heterogeneity among studies [125, 126]. In studies with more than one of the above comparisons, the sample size was proportionately divided to facilitate comparison across interventions. As tV̇O_2_max may be affected by work interval duration [10], and exercise intensity distribution [30], these factors were examined in subcategory analyses using Chi^2^ tests. Effect sizes were interpreted according to Hopkins et al. [127]: SMD/Hedges’ *g* < 0.2 = trivial, 0.2–0.59 = small, 0.6–1.19 = moderate, 1.2–1.99 = large. Heterogeneity was assessed using the *I*^2^ statistic, with values of <25%, 25%−75%, and >75% representing low, moderate, and high levels of heterogeneity, respectively. All analyses were carried out using RevMan (Version 5.3), with significance levels set at *p* ≤.05.

## Results

### Study selection and characteristics

A total of 86 studies were included in the present investigation (see Fig. 1 and Table 1), and tV̇O_2_max was determined for a total of 239 HIIT protocols (see Table 2). In the included studies, tV̇O_2_max was most commonly expressed as time ≥ 90% V̇O_2_max [31, 38, 39, 46–48, 50, 52, 53, 55, 58, 62–64, 66, 69, 71–73, 76, 77, 80, 84, 89, 90, 92, 96, 105, 107–111, 113, 117–119], but also as time ≥ 80% V̇O_2_max [45, 57, 74], time ≥ 85% V̇O_2_max [44], time ≥ 95% V̇O_2_max [34, 41, 51, 68, 79, 93, 98, 114], time ≥ 100% V̇O_2_max [33, 81], or by a combination of variables [29, 37, 42, 43, 49, 54, 56, 60, 61, 65, 70, 75, 78, 82, 83, 85–88, 91, 94, 95, 97, 102–104, 106, 112, 115, 116]. In two studies, partially duplicated data were identified and excluded from further analysis [66, 90].

Twenty-two studies reported tV̇O_2_max in absolute values and worked out tV̇O_2_max as a fraction of either the cumulative HIIT protocol duration [37, 46, 65, 76, 82, 90, 91, 105] or total work interval duration [34, 43, 50, 54, 64, 68, 69, 74, 82, 98, 114, 116]. In two studies, it was unclear how the reported fraction was calculated [51, 97], and four studies only reported tV̇O_2_max as a fraction with no further explanation [59, 67, 99, 100].

Indexed for tV̇O_2_max, 133 HIIT protocols accumulated tV̇O_2_max ≥ 5 min, of which 48 protocols achieved tV̇O_2_max ≥ 10 min (see Table 2 and Supplementary Table S2 for absolute tV̇O_2_max reported in all included studies).

### Study quality and risk of bias assessment

**Fig. 2:**
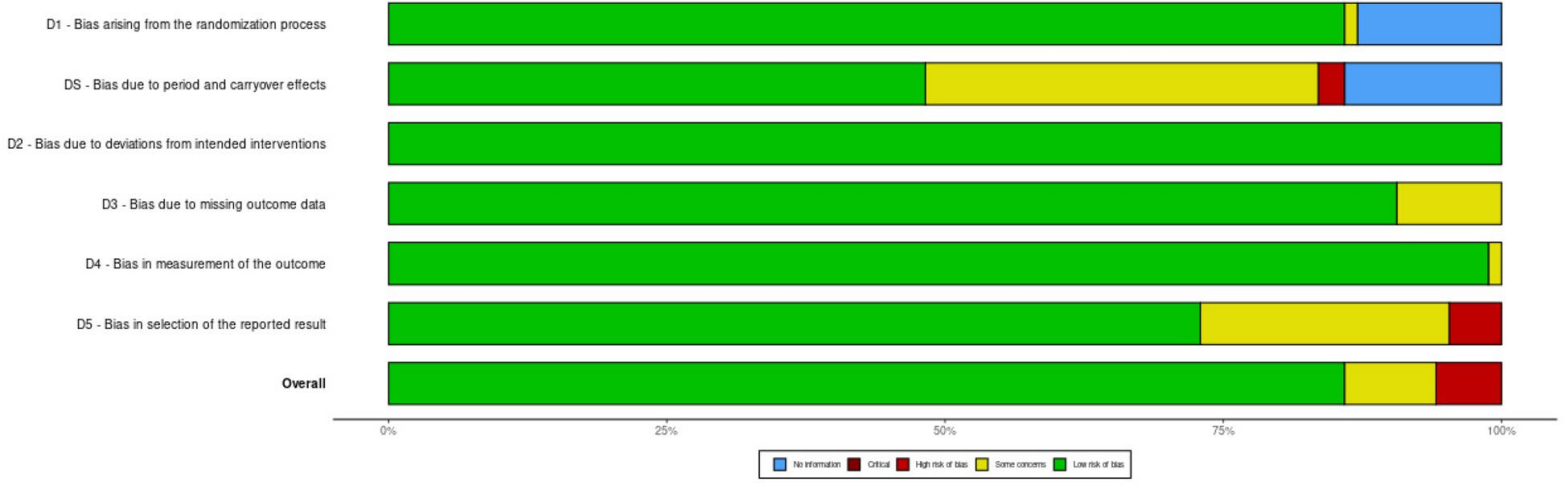
Risk of bias summary plot of Rob-2 assessments

Study quality, as assessed by the PEDro scale, had a rating of 5.6 ± 0.7, indicating included studies were, on average, of good to excellent quality. Risk of bias was generally low across the included studies (see Fig. 2). Across all studies, objective physiological measures were obtained using reputable cardiopulmonary exercise testing devices [128]. Some concern on bias arose from studies incorporating multiple HIIT sessions, as training adaptations may have increased V̇O_2_max, subsequently influencing tV̇O_2_max. Individual results of the PEDro scale and RoB-2 can be found in Supplementary Table S3.

### Time ≥ 90% V̇O_2_max

Time ≥ 90% V̇O_2_max was reported for 183 HIIT protocols and was different between SI-HIIT, MI-HIIT and LI-HIIT protocols (see Table 3; [F(2,179) = 8.038, *p* <.001]), after adjusting for the different cumulative HIIT protocol durations across these subcategories [F(2,180) = 14.21, *p* <.001]. Post hoc tests indicated greater time ≥ 90% V̇O_2_max in LI-HIIT compared to both SI-HIIT and MI-HIIT (*p* <.01 and *p* <.001 respectively), with differences of moderate magnitude (Hedges’ *g* = 0.87 and 1.06 respectively). Total work interval duration differed between subcategories [F(2,180) = 30.54, *p* <.001], with longer durations in LI-HIIT compared to SI-HIIT and MI-HIIT (*p* <.001).

**Table 3:** Time ≥ 90% V̇O_2_max between HIIT protocols

	SI-HIIT (*n*=62)	MI-HIIT (*n*=35)	LI-HIIT (*n*=86)
Time ≥ 90% V̇O_2_max (s)	336 ± 273	322 ± 174	555 ± 234*
Total work interval duration (s)	659 ± 586	694 ± 355	1296 ± 566*
Cumulative HIIT protocol duration (s)	1390 ± 838	1431 ± 887	2058 ± 799*

tV̇O_2_max of HIIT jumping protocols [77], protocols with variable work interval durations [53, 111], and studies with tV̇O_2_max reported as fraction with no further explanation were excluded [59, 67, 99, 100]

^*^p <.01, LI-HIIT vs SI-HIIT and MI-HIIT

**Fig. 3:**
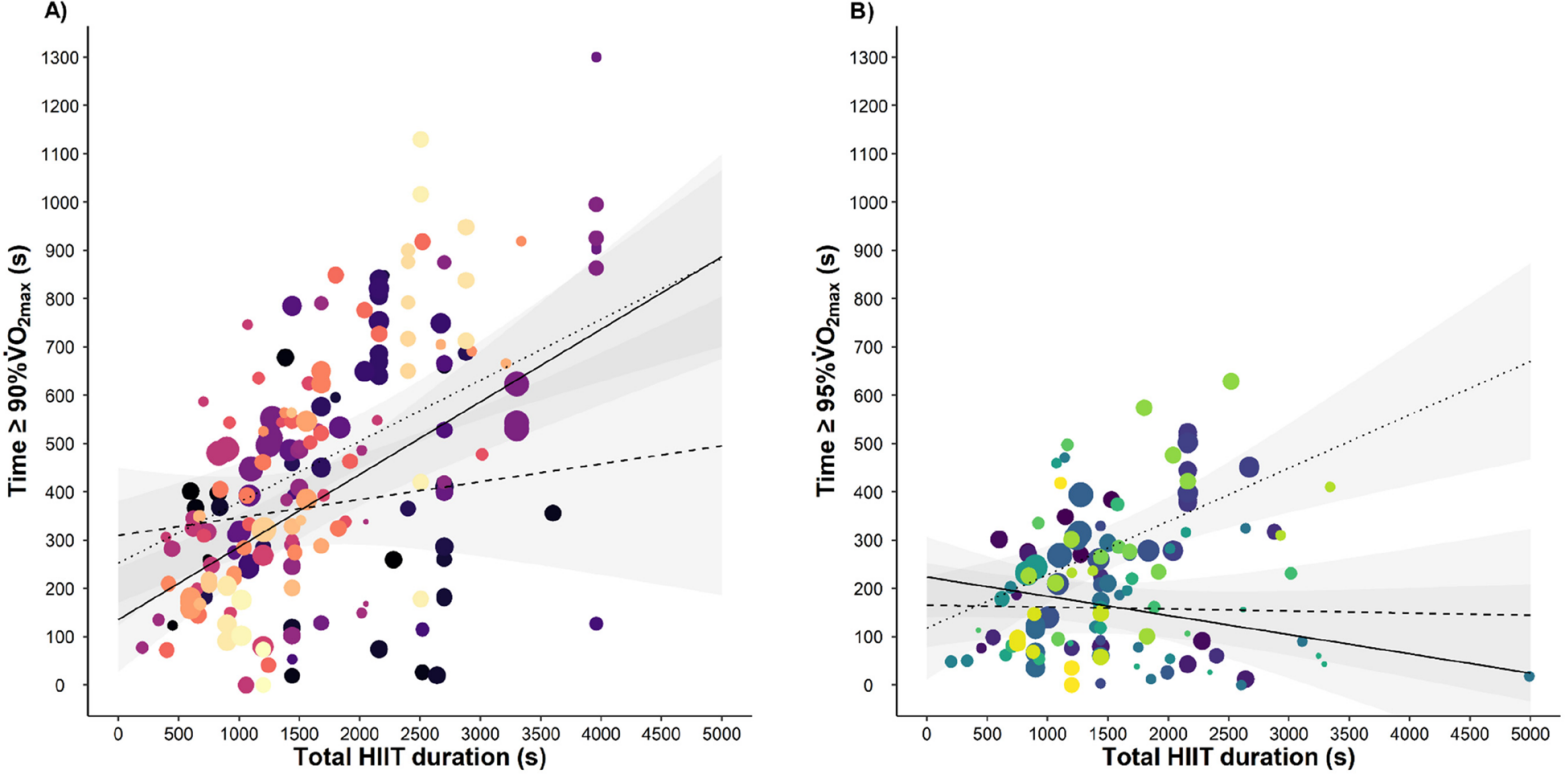
Relationship between cumulative HIIT duration and time ≥ 90%V̇O_2max_ (panel **A**) and time ≥ 95%V̇O_2max_ (panel **B**). Each point represents a HIIT protocol, coloured by study and sized by sample size. The lines show model predictions based on work interval length (solid for SI-HIIT, dashed for MI-HIIT, and dotted for LI-HIIT protocols). Shaded areas represent 95% confidence intervals around fixed-effect predictions

In the linear mixed-effects models, adding exercise modality did not improve model fit compared with the base model, containing cumulative HIIT protocol duration alone (χ^2^(5) = 8.80, *p* = 0.117). Including work interval duration did not further improve model fit compared with the base model (χ^2^(2) = 5.31, *p* = 0.070). However, including the interaction between work interval length and cumulative HIIT protocol duration improved fit relative to the base model (χ^2^(4) = 10.39, *p* = 0.034). The interaction model was therefore retained (see Fig. 3, panel A). Time ≥ 90% V̇O_2_max was associated with cumulative HIIT protocol duration among SI-HIIT, increasing by approximately 0.15 s for each additional second of HIIT (*p* <.001). The slope for MI-HIIT was flatter compared with SI-HIIT, with a difference of −0.112 s per second of HIIT (*p* = 0.027). The slope for LI-HIIT was not different from the slope for SI-HIIT (*p* = 0.483). Model-predicted equations are as follows:

- SI-HIIT: Time ≥ 90%V̇O_2_max = 135.4 + 0.15 × Cumulative HIIT duration
- MI-HIIT: Time ≥ 90%V̇O_2_max = 309.6 + 0.04 × Cumulative HIIT duration
- LI-HIIT: Time ≥ 90%V̇O_2_max = 254.7 + 0.13 × Cumulative HIIT duration

The marginal R^2^ for the final model was 0.064, indicating that cumulative HIIT protocol duration and work interval length accounted for approximately 6.4% of the variance in time ≥ 90% V̇O_2_max across all HIIT protocols. The conditional R^2^ was 0.162, meaning that the combination of fixed (i.e. cumulative HIIT duration and work interval length) and random effects (i.e. study-level variability) accounted for 16.2% of the total variance. The adjusted ICC was 0.104, indicating that approximately 10.4% of the total variance in time ≥ 90% V̇O_2_max was attributable to clustering within studies. This suggests a modest but non-negligible degree of dependence among observations originating from the same study, which justifies the inclusion of a random intercept for study in the model.

### Time ≥ 95% V̇O_2_max

One hundred and seventeen HIIT protocols evaluated time ≥ 95% V̇O_2_max (see Table 4). Time ≥ 95% V̇O_2_max was significantly different between SI-HIIT, MI-HIIT and LI-HIIT protocols [F(2,113) = 22.459, *p* <.001], after adjusting for non-significant differences in cumulative HIIT protocol durations [F(2,114) = 1.174, *p* = 0.185]. Post hoc tests revealed time ≥ 95% V̇O_2_max was higher in LI-HIIT compared to both SI-HIIT and MI-HIIT, with differences of moderate to large magnitude (Hedges’ *g* = 1.15 and 1.52 respectively). Total work interval duration was different between the subcategories [F(2,114) = 11.31, *p* <.001], with longer total work interval durations in LI-HIIT compared to both SI-HIIT and MI-HIIT (*p* <.001).

**Table 4:** Differences in time ≥ 95% V̇O_2_max between HIIT protocols

	SI-HIIT (*n*=46)	MI-HIIT (*n*=27)	LI-HIIT (*n* =44)
Time ≥ 95% V̇O_2_max (s)	162 ± 140	137 ± 94	319 ± 132*
Total work interval duration (s)	673 ± 434	762 ± 483	1084 ± 371*
Cumulative HIIT protocol duration (s)	1476 ± 736	1497 ± 985	1749 ± 584

^*^p <.001, LI-HIIT vs SI-HIIT and MI-HIIT

In the linear mixed-effects models, including work interval length improved model fit compared with the model with cumulative HIIT protocol duration alone (χ^2^(2) = 21.07, *p* <.001), whereas adding exercise modality did not (χ^2^(4) = 4.72, *p* = 0.317). Including the interaction between work interval length and cumulative HIIT protocol duration further improved fit relative to the additive model (χ^2^(2) = 16.13, *p* <.001). The interaction model was therefore retained (see Fig. 3, panel B). Time ≥ 95% V̇O_2_max was not associated with cumulative HIIT protocol duration among SI-HIIT protocols (*p* = 0.117). The nearly zero slope for MI-HIIT was not different from the slope for SI-HIIT (*p* = 0.309). The slope for LI-HIIT increased by approximately 0.15 s per second of HIIT compared with SI-HIIT (*p* <.001). Model-predicted equations are as follows:

- SI-HIIT: Time ≥ 95%V̇O_2_max = 223.5 − 0.040 × Cumulative HIIT duration
- MI-HIIT: Time ≥ 95%V̇O_2_max = 165.4 − 0.004 × Cumulative HIIT duration
- LI-HIIT: Time ≥ 95%V̇O_2_max = 126.4 + 0.107 × Cumulative HIIT duration

The marginal R^2^ for the final model was 0.072, indicating that cumulative HIIT protocol duration and work interval length accounted for approximately 7.2% of the variance in time ≥ 95% V̇O_2_max across all HIIT protocols. The conditional R^2^ was 0.138, meaning that the combination of fixed (i.e. cumulative HIIT duration and work interval length) and random effects (i.e. study-level variability) accounted for 13.8% of the total variance. The adjusted ICC was 0.071, indicating that approximately 7.1% of the total variance in time ≥ 95% V̇O_2_max was attributable to clustering within studies. Again, this suggests a modest but non-negligible degree of dependence among observations originating from the same study, which justifies the inclusion of a random intercept for study in the model.

### Meta-analysis results

#### Work interval duration

**Fig. 4:**
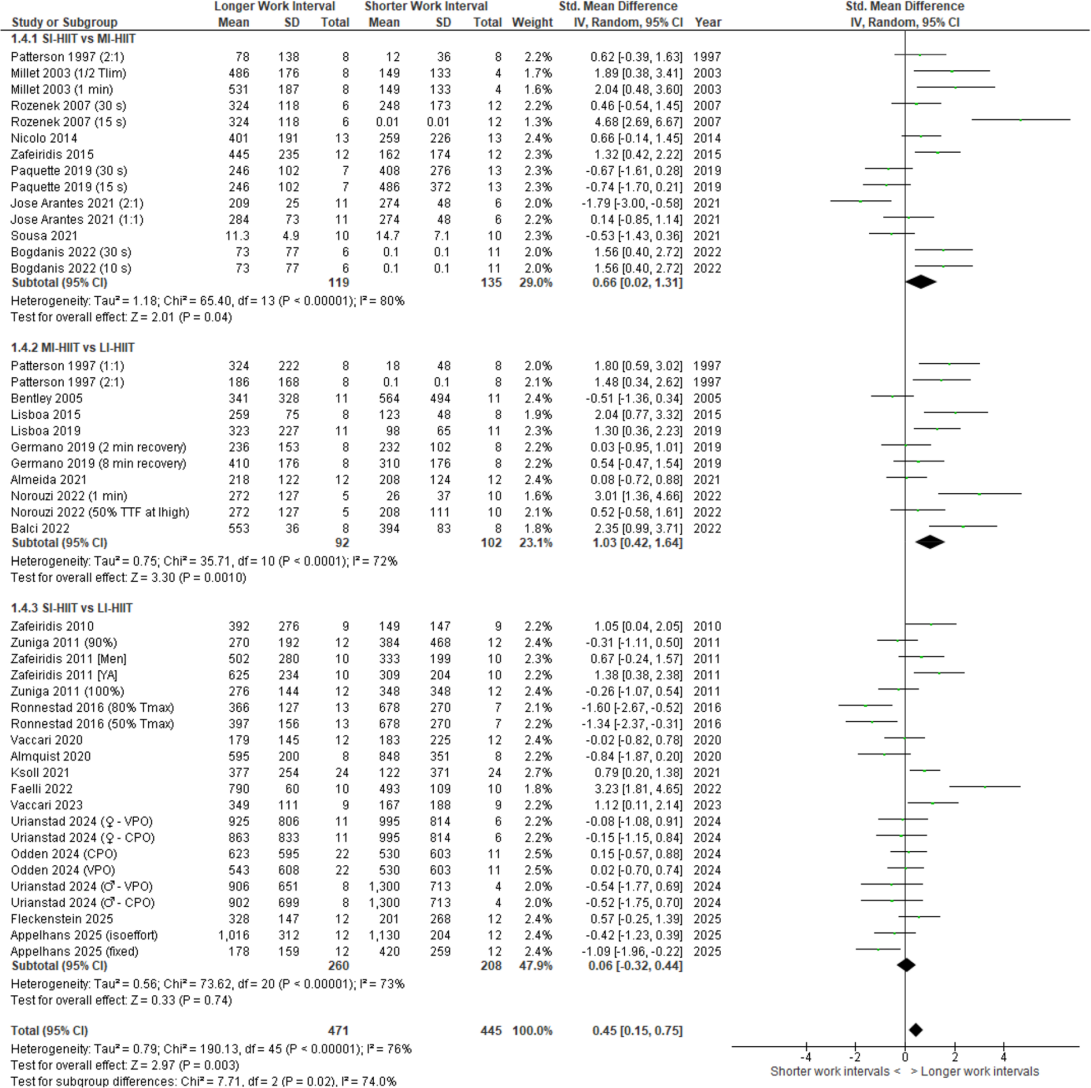
Forest plot of tV̇O_2_max across SI-, MI-, and LI-HIIT protocols

The meta-analysed effect for tV̇O_2_max across the included studies, which compared SI-HIIT, MI-HIIT, and LI-HIIT protocols, indicated small differences in tV̇O_2_max (SMD = 0.45 [95% CI: 0.15–0.75], Z = 2.97, *p* <.001). Heterogeneity among studies was high (I^2^ = 76%, *p* <.01). Subcategory analysis revealed differences (χ^2^(2) = 7.71, *p* <.02; I^2^ = 74%), with LI-HIIT eliciting greater tV̇O_2_max than MI-HIIT (Z = 3.30, *p* <.001*)*, and MI-HIIT compared to SI-HIIT (Z = 2.01, *p* = 0.04, see Fig. 4).

#### Active vs passive recovery intervals

**Fig. 5:**
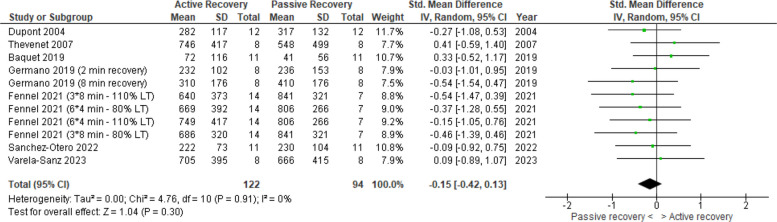
Forest plot of tV̇O_2_max across HIIT protocols, comparing active to passive recovery intervals

No differences in tV̇O_2_max were observed when comparing active to passive recovery intervals (see Fig. 5, SMD = −0.15 [95% CI: −0.42 to 0.13], Z = 1.04, *p* =.30). In exhaustive HIIT protocols [37, 85, 102, 106, 110], the number of completed work-recovery cycles, and with that, time spent at high exercise intensities was notably greater when passive recovery intervals were incorporated (909 ± 443 s vs. 582 ± 433 s; F(5) = 27.433, *p* <.01, Hedges’ *g* = 0.67).

### Exercise intensity distribution in work intervals

**Fig. 6:**
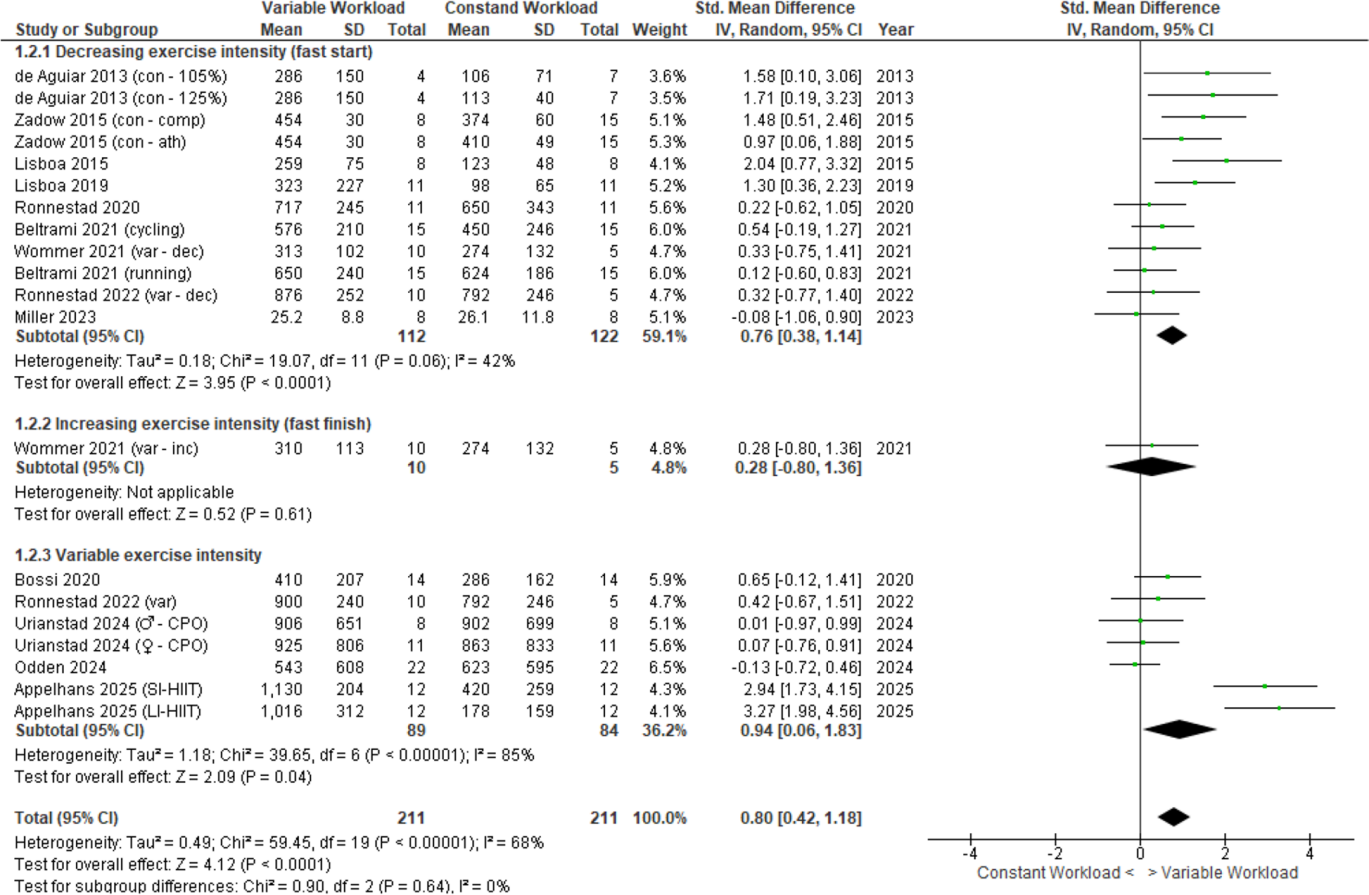
Forest plot of tV̇O_2_max across HIIT protocols, comparing variable-intensity work intervals to even-paced formats

The pooled mean estimate showed a higher tV̇O_2_max when HIIT protocols used variable-intensity compared with even-paced work intervals (SMD = 0.80 [95% CI: 0.40–1.18], Z = 4.12, *p* <.01). The overall effect was of a moderate magnitude, with substantial heterogeneity among studies (I^2^ = 68%, *p* <.01). Subcategory analyses suggested larger effects for variable- and decreasing-intensity work intervals (see Fig. 6); however, the test for subcategory differences was not significant (χ^2^(2) = 0.90, *p* =.64; I^2^ = 0%). Excluding results presented as unspecified fractions [67], the mean difference indicated an additional increase in tV̇O_2_max of 165 s [95% CI: 97–223 s] for protocols with variable-intensity work intervals.

## Discussion

The present review summarises how much time individuals spent at, or near V̇O_2_max across a wide variety of HIIT protocols and exercise modalities. Of the 239 HIIT protocols included, 133 elicited at least 5 min at tV̇O_2_max, and 48 protocols exceeded 10 min. Our results show that time ≥ 90% and ≥ 95% V̇O_2_max are higher during HIIT protocols that incorporate long (≥ 2 min), compared with short (≤ 30 s) and moderate duration work intervals (> 30 s to 2 min). Cumulative HIIT protocol duration and work interval length accounted for approximately 6.4% and 7.2% of the variance in time ≥ 90% V̇O_2_max and time ≥ 95% V̇O_2_max, respectively. In addition, variable-intensity work intervals were found to increase tV̇O_2_max compared with even-paced work intervals, while the use of active or passive recovery between work intervals had no effect on tV̇O_2_max. Together, these findings highlight that both the structure and cumulative duration of HIIT sessions exert an influence on tV̇O_2_max, providing researchers, practitioners, and athletes with a firmer foundation for understanding how specific protocols shape the physiological demands and adaptive potential of a workout.

Midgley & McNaughton were the first to collate tV̇O_2_max from 12 HIIT running protocols [32], and recommended that both work and recovery intervals should last between 15 to 30 s to maximise tV̇O_2_max, with work intervals performed at near maximal running speeds. By expanding the scope of our review to include cycling, jumping, kayaking, rowing, swimming, and cross-country skiing, we provide updated insights into tV̇O_2_max. Midgley & McNaughton’s recommendations were largely based on HIIT protocols performed to exhaustion [32], an approach considered uncommon and impractical for high-performance athletes [129]. In comparison, ~60% of the included protocols in our review incorporated a preset and fixed number of repetitions, with work interval durations ranging from 6 to 480 s (see Table 2). Our results indicate that HIIT protocols with longer work intervals yield greater time ≥ 90% and ≥ 95% V̇O₂max compared to protocols with shorter work intervals. To elicit high V̇O₂ during HIIT, work intervals must be long enough for these levels to be reached and maintained [3], which contrasts earlier recommendations [32]. Although SI-HIIT and, to a lesser extent, MI-HIIT are generally considered to primarily target neuromuscular adaptations [13, 23], these protocols can still produce high V̇O₂ levels when work intensities are high and recovery intervals brief, as V̇O_₂_ oscillates minimally around V̇O₂max after an initial rise (see e.g. [52, 81, 87]). However, in line with the categorisation of HIIT protocols [3, 10], our findings reinforce that incorporating longer work intervals elicit greater durations at high oxygen uptake. Our linear mixed-effects models further confirmed a positive relationship between cumulative HIIT duration and time ≥ 90% and ≥ 95% V̇O₂max when longer work intervals are used.

To increase tV̇O_2_max during HIIT sessions, Midgley & Naughton [32] also suggested that a 10 to 15-min warm-up should be immediately followed by the first work interval to initiate these from an elevated V̇O_2_, alongside active recovery intervals. In practice, active recovery is reported to be psychologically demanding [3, 129] and has been shown to decrease exercise time at high intensities during exhaustive HIIT protocols [37, 85, 102, 106, 110]. Our results further indicate that active recovery does not increase tV̇O_2_max. In running-based LI-HIIT [101, 103] and in square wave transitions in cycling [130], starting work intervals from a lower metabolic rate prolonged time to reach a V̇O₂ plateau, but this delay was offset by a greater V̇O₂ amplitude, resulting in comparable tV̇O₂max values relative to protocols starting from a higher metabolic baseline [101, 103]. Collectively, these findings suggest that passive recovery may be equally effective for facilitating tV̇O_2_max attainment, while also helping individuals to sustain high intensities for longer. Surprisingly, few studies employed a ‘*priming’* warm-up, with most opting for low intensity protocols (see Supplementary Table S4). Priming, a brief bout of moderate- to heavy-intensity exercise before the main session, is thought to accelerate V̇O₂ kinetics by elevating mitochondrial calcium and activating mitochondrial enzymes [131]. Without priming, initial work intervals may act as the priming stimulus, as shown by McKee et al. [58], who reported significantly greater time spent ≥ 90% V̇O_2_max during the second (87.8 ± 28.5 s) and third (98.6 ± 26.2 s) work intervals compared to the first (59.7 ± 28.9 s). Together, these findings suggest a complementary strategy: a priming warm-up accelerates early V̇O_2_ kinetics, while starting subsequent intervals from a lower metabolic rate maximises tV̇O_2_max. Passive or low intensity recovery during HIIT sessions following priming, may therefore combine the benefits of both approaches.

More recently, the exercise intensity distribution within work intervals was proposed to influence time spent at higher fractions of V̇O_2_max [30]. Supporting this hypothesis, our results evidence that an increase in tV̇O_2_max of 165 s [95% CI: 97–223 s] can be achieved when work intervals are performed with decreasing [43, 44, 51, 55, 64, 67, 98, 117, 118], increasing [64], or variable exercise intensities [31, 38, 71, 72, 118] compared to even-paced work intervals. Higher exercise intensities at the onset of exercise bouts initiate a quick rise in V̇O₂ [132, 133], and may increase the oxygen cost of hyperpnoea [31]. Analysing electromyographic (EMG) activity of the vastus lateralis in cycling HIIT, Lisbôa et al. [43] reported that an initial high exercise intensity may accelerate the recruitment and activation of high-threshold type II fibres, and the effect on the O_2_ cost and tV̇O_2_max becomes more evident during the later stages of HIIT sessions. In addition to manipulating work intensities within HIIT sessions, cycling-based HIIT performed on a vibration platform appeared to increase EMG activity and yield a small additional increase in tV̇O_2_max [48, 62, 66]. However, this likely depends on factors such as total vibration exposure, exercise intensity, and the specific vibration protocol used, with long-term adaptations still unclear.

Quantifying tV̇O_2_max remains a popular approach for describing the adaptive potential of HIIT protocols, yet few studies have assessed tV̇O_2_max alongside long-term adaptations or performance improvements [38, 39, 73, 97, 119]. Several studies have failed to demonstrate a clear dose–response relationship. For example, despite meaningful differences in tV̇O_2_max during a seven-week intervention comparing active and passive recovery, improvements in V̇O_2_max did not differ between groups [97]. Similarly, despite markedly lower tV̇O_2_max, Turnes et al. [39] reported greater V̇O_2_max improvements in cyclists who performed short intervals (+6.3%) compared with those who completed long intervals near their critical power (+3.3%). A comparable pattern was observed in participants who trained in cooler conditions, spending more time ≥ 90% V̇O_2_max than those in the heat, while 20-km time trial improvements remained similar. [73]. In contrast, Rønnestad et al [119] reported a moderate (non-significant) correlation between time ≥ 90% V̇O_2_max and subsequent V̇O_2_max improvements (r = 0.54). More compellingly, Odden et al. [38] continuously monitored V̇O₂ during all HIIT sessions of a nine-week intervention and found that higher fractions of V̇O_2_max predicted greater improvements in peak power output (adjusted r^2^ = 0.44, *p* <.01) and V̇O_2_max (adjusted r^2^ = 0.54, *p* <.05). The above mixed findings highlight the difficulty of drawing firm conclusions from a small number of studies, with heterogeneous methodologies and participant groups, and in some cases, conclusions based on only a single assessment of tV̇O_2_max. These studies also challenge the suggestion that tV̇O_2_max per HIIT session must exceed 5 min, or even 10 min, to elicit adaptations [3], as improvements in V̇O_2_max have been observed with in tV̇O_2_max < 5 min [39], between 5 and 10 min [73, 97], and ≥ 10 min tV̇O_2_max [38, 73, 119]. Given that increases in V̇O_2_max are closely linked to central adaptations, including cardiac output, stroke volume and the maximal arteriovenous O_2_ difference, protocols eliciting sustained high fractions of maximal heart rate have been associated with substantial improvements in V̇O_2_max, even with limited tV̇O_2_max [36, 60]. The magnitude of change in V̇O_2_max is likely further influenced by participant training status, and the total duration of the training interventions [13, 134].

### Limitations and Future directions

This review identified several HIIT configurations that may maximise tV̇O_2_max, yet important questions remain regarding their physiological and performance relevance. The rigorous methodological approach used by Odden et al. [38] provides a valuable framework for linking acute responses to long-term adaptations. Across studies, substantial variation existed in HIIT protocols (see Table 2) and in both intra- and inter-individual tV̇O₂max physiological responses (see Supplementary Table S2). Reflecting this, tV̇O₂max showed poor-to-moderate reliability in HIIT performed to exhaustion [70, 87], as well as in predefined sessions [75]. Across studies, ICCs ranged between 0.57 and 0.90 [70, 75, 87]; however, ICC values in isolation can be misleading, as they are influenced by between-subject variability and session-to-session changes [135], as reflected by the large coefficients of variation reported for time ≥ 90% and ≥ 95% V̇O₂max. Between studies, variability in tV̇O₂max was further influenced by methodological differences, including VO_₂_ data smoothing and time-averaging techniques, and different thresholds used to define tV̇O₂max (see Supplementary Table S4). Short averaging intervals can inflate V̇O₂max due to noise from irregular ventilatory patterns, artificially shifting tV̇O₂max threshold for subsequent HIIT sessions beyond achievable levels. Notably, using short averaging intervals to determine V̇O_2_max while averaging VO_₂_ data over longer periods to assess tV̇O_2_max in HIIT sessions can make tV̇O_2_max appear shorter, whereas the inverse combination can produce the opposite effect [88, 136]. It remains unclear whether intra- and inter-individual variability represents ‘biological noise’ or indeed an inconsistent training response to HIIT [70, 87, 119]. Indexing tV̇O₂max to same-day V̇O₂max reduced inter-individual variation in exhaustive continuous runs [85] and cycling HIIT sessions [75], although substantial variability persisted. This highlights that inter-individual variability can only be meaningfully interpreted when intra-individual variability is accounted for [70], and single measurements of tV̇O₂max should be interpreted with caution. To improve reliability and ensure robust comparisons, future studies should acquire gas exchange data across identical HIIT sessions and report tV̇O₂max as the mean of repeated trials [31, 63]. Additionally, ensemble averaging methods, recently applied in the field of oxygen kinetics and muscle deoxygenation research [137, 138], may offer further improvements in precision and reduce measurement noise, supporting more robust assessments of HIIT protocols and participant responses.

It is important to note that categorising HIIT protocols solely by work interval duration can lead to misclassification, potentially influencing our findings. For example, protocols with repeated short ‘on’/‘off’ transitions were originally described as SI-HIIT [52, 139], but have since been reclassified as LI-HIIT when the series are treated as one single work interval (e.g. in [38]; 11 repetitions of 30 s ‘on’/15 s ‘off’ is considered one 7 min 45 s work interval). In the present study, we adhered to the original categorisation as set out by Wen et al. [10], indexing protocols with the series format as SI-HIIT, consistent with their work interval duration [38, 52, 59, 63, 69, 71, 72, 76, 78, 83, 84, 86]. Many of these studies contributed to the meta-analytic estimates comparing tV̇O_2_max between SI-HIIT, MI-HIIT and LI-HIIT, not only influencing the effect sizes, but also obscuring the true comparisons between protocols of different work interval durations (Fig. 4). Even within categories, discrepancies exist; for example, 3-min and 8-min intervals differ substantially in in physiological demands and exercise intensities yet were treated equivalently. Maintaining prior classifications ensured comparability with previous literature, despite some loss of nuance, but existing discrepancies in HIIT terminology highlight the limitations of duration-based classifications and the need for clearer, standardised criteria. Future research should therefore treat work interval duration as a continuous variable, allowing for more precise evaluation. In young swimmers [115, 116], and more recently in cyclists [74], time spent ≥ VT2 has been proposed as a novel, physiologically grounded alternative method to define time in the *‘red zone’.* Adopting this individualised approach resulted in notable higher tV̇O_2_max compared to 80%, 90% and 95% V̇O_2_max [74, 115, 116]. Future studies are warranted to validate time spent ≥ VT2 as an alternative to fixed V̇O₂max fractions for quantifying the physiological stimulus of HIIT. This could be achieved using the methodological blueprint of Odden et al. [38], with continuous V̇O₂ monitoring during HIIT sessions and subsequent analysis of the data against multiple tV̇O₂max thresholds, including both time ≥ VT2 and traditional fixed fractions. Such an approach would provide clear insight into which physiological metrics best capture the adaptive stimulus of HIIT and help identify more robust predictors of training responsiveness.

### Practical applications

The present review provides a synthesis of responses across a wide range of HIIT protocols, which can serve as a practical reference for selecting and comparing exercise formats in applied settings. Our findings are primarily derived from laboratory-based assessments of V̇O₂, and the monitoring of tV̇O₂max during HIIT sessions may not transfer as readily to applied settings compared with the widespread use of heart rate monitoring. However, heart rate and V̇O₂ responses may not demonstrate a sufficiently strong or consistent relationship to serve as interchangeable markers of exercise intensity during intermittent exercise [103, 140].

The regression equations provide a practical means of estimating tV̇O₂max from commonly reported training variables, facilitating comparison and prescription of HIIT protocols. Theoretically, the application of the regression equations to standardised 30-min HIIT sessions suggests that exercise structure may meaningfully influence predicted tV̇O₂max, despite equivalent total session duration. As illustrated in Table 5, model-derived estimates indicate higher predicted tV̇O₂max for LI-HIIT compared with SI- and MI-HIIT formats. Our findings further suggest that variable work intensities within intervals and passive recovery intervals are likely effective strategies for maximising tV̇O₂max during HIIT sessions.

**Table 5:** Predicted tV̇O₂max for standardised HIIT sessions based on regression equations

**HIIT Category**	**HIIT Protocol**	Cumulative duration (s)	Predicted time ≥ 90% VO_2_max (s)
SI-HIIT	6 × 30 s Wingate sprint, 4:30 min passive rest	1800	152
MI-HIIT	15 × 1 min isoeffort, 1 min passive rest	1800	158
LI-HIIT	5 × 4 min isoeffort, 2 min passive rest	1800	319

## Conclusion

This systematic review demonstrates that HIIT protocols incorporating work intervals longer than 2 min and greater total session durations elicit greater tV̇O_2_max compared to shorter protocols. While strategies such as fast starts, variable intensities, and priming warm-ups can further enhance oxygen uptake kinetics, recovery mode (active vs. passive) appears to exert little influence on tV̇O_2_max. Although tV̇O₂max is widely used as an indicator of internal training load, the present synthesis highlights substantial intra- and inter-individual variability, as well as methodological diversity in how VO₂ responses are quantified. Rather than detracting from its utility, this variability underscores the importance of interpreting tV̇O₂max within the context of protocol design, participant characteristics and complementary physiological markers. Importantly, evidence from intervention studies indicates that improvements in V̇O₂max are not strictly dependent on accumulating fixed durations (e.g. ≥ 5 or ≥ 10 min in *‘the red zone’*)*,* reinforcing that multiple configurations of HIIT can drive adaptations. Future research should adopt standardised reporting methods and consider individualised thresholds such as VT2 to improve the precision and applicability of training load quantification.

## Supplementary Information

Supplementary Material 1.

Supplementary Material 2.

Supplementary Material 3.

Supplementary Material 4.

Supplementary Material 5.

## Data Availability

All extracted data from the included studies are reported in table 1 and 2 (and supplementary material). Data are available upon request from the corresponding author.

## References

[1] Billat LV. Interval training for performance: a scientific and empirical practice, Part 1. Sports Med. 2001;31:75–90. 10.2165/00007256-200131020-00001. 11227980 10.2165/00007256-200131020-00001

[2] Laursen PB. Training for intense exercise performance: high-intensity or high-volume training? Scand J Med Sci Sports. 2010;20(Suppl 2):1–10. 10.1111/j.1600-0838.2010.01184.x. 20840557 10.1111/j.1600-0838.2010.01184.x

[3] Buchheit M, Laursen PB. High-intensity interval training, solutions to the programming puzzle: Part I: cardiopulmonary emphasis. Sports Med. 2013;43:313–38. 10.1007/s40279-013-0029-x. 23539308 10.1007/s40279-013-0029-x

[4] Daniels J, Scardina N. Interval training and performance. Sports Med An Intern J Appl Med Sci Sport Exerc. 1984;(4):327–34. 10.2165/00007256-198401040-00006. 10.2165/00007256-198401040-00006 6390607

[5] Åstrand I, Åstrand P-O, Christensen EH, Hedman R. Intermittent muscular work. Acta Physiol Scand. 1960;48:448–53. 10.1111/j.1748-1716.1960.tb01879.x. 13794890 10.1111/j.1748-1716.1960.tb01879.x

[6] Christensen EH, Hedman R, Saltin B. Intermittent and continuous running (A further contribution to the physiology of intermittent work.). Acta Physiol Scand. 1960;50:269–86. 10.1111/j.1748-1716.1960.tb00181.x. 14448704 10.1111/j.1748-1716.1960.tb00181.x

[7] Fox EL, Bartels RL, Billings CE, Mathews DK, Bason R, Webb WM. Intensity and distance of interval training programs and changes in aerobic power. Med Sci Sports. 1973;5(1):18–22. 4721844

[8] Knuttgen, H.G., Nordesjo, L.-O., Ollander, B., Saltin B. Physical conditioning through interval training with young male adults. Med. Sci. Sports. 1973: 220–6. 10.1249/00005768-197300540-00002. 4774198

[9] Ekkekakis P, Swinton P, Tiller NB. Extraordinary claims in the literature on High-Intensity Interval Training (HIIT): I. Bonafide scientific revolution or a looming crisis of replication and credibility? Sports Med. 2023;(10):1865–90. 10.1007/s40279-023-01880-7. 10.1007/s40279-023-01880-7 37561389

[10] Wen D, Utesch T, Wu J, Robertson S, Liu J, Hu G, et al. Effects of different protocols of high intensity interval training for VO2max improvements in adults: a meta-analysis of randomised controlled trials. J Sci Med Sport. 2019;22:941–7. 10.1016/J.JSAMS.2019.01.013. 30733142 10.1016/j.jsams.2019.01.013

[11] Rosenblat MA, Granata C, Thomas SG. Effect of interval training on the factors influencing maximal oxygen consumption: a systematic review and meta-analysis. Sports Med. 2022;(6):1329–52. 10.1007/s40279-021-01624-5. 10.1007/s40279-021-01624-5 35041180

[12] Yang Q, Wang J, Guan D. Comparison of different interval training methods on athletes’ oxygen uptake: a systematic review with pairwise and network meta-analysis. BMC Sports Sci Med Rehabil. BioMed Central Ltd; 2025;17 10.1186/s13102-025-01191-6. 10.1186/s13102-025-01191-6 PMC12218014 40605061

[13] Wiesinger HP, Stöggl TL, Haller N, Blumkaitis J, Strepp T, Kilzer F, et al. Meta-analyses of the effects of high-intensity interval training in elite athletes—part I: mean effects on various performance measures. Front. Physiol. Frontiers Media SA; 2024. 10.3389/fphys.2024.1486526. 10.3389/fphys.2024.1486526 PMC11739151 39830026

[14] García-Pinillos F, Soto-Hermoso VM, Latorre-Román PA. How does high-intensity intermittent training affect recreational endurance runners? Acute and chronic adaptations: A systematic review. J Sport Health Sci. 2017;6(1):54–67. 10.1016/j.jshs.2016.08.010. 10.1016/j.jshs.2016.08.010 PMC6188912 30356547

[15] Rosenblat MA, Perrotta AS, Thomas SG. Effect of high-intensity interval training versus sprint interval training on time-trial performance: a systematic review and meta-analysis. Sports Med. 2020;(6):1145–61. 10.1007/s40279-020-01264-1. 10.1007/s40279-020-01264-1 32034701

[16] Norte B, Steele J, Wright J. Physiological and performance adaptations to interval training in endurance-trained cyclists: an exploratory systematic review and meta-analysis. Intern J Strength Condition 2024;4. 10.47206/ijsc.v4i1.271.

[17] Joyner MJ, Coyle EF. Endurance exercise performance: the physiology of champions. J Physiol. 2008;(1):35–44. 10.1113/jphysiol.2007.143834. 10.1113/jphysiol.2007.143834 PMC2375555 17901124

[18] Mølmen KS, Almquist NW, Skattebo Ø. Effects of exercise training on mitochondrial and capillary growth in human skeletal muscle: a systematic review and meta-regression. Sports Med. 2025;(1):115–44. 10.1007/s40279-024-02120-2. 10.1007/s40279-024-02120-2 PMC11787188 39390310

[19] Bi Z, Yin M, Xu K, Marcotte-Chénard A, Zhong Y, Gu Z, et al. One size does not fit all: a meta-analysis of 115 trials comparing high-intensity interval and moderate-to-vigorous-intensity continuous training across diverse participants, protocols, and outcomes. Scand J Med Sci Sports. John Wiley & Sons, Ltd; 2026;36(3):e70243. 10.1111/sms.70243. 10.1111/sms.70243 41804294

[20] Seiler S, Haugen O, Kuffel E. Autonomic recovery after exercise in trained athletes: intensity and duration effects. Med Sci Sports Exerc. 2007;39:1366–73. 10.1249/mss.0b013e318060f17d. 10.1249/mss.0b013e318060f17d 17762370

[21] Stöggl TL, Sperlich B. The training intensity distribution among well-trained and elite endurance athletes. Front Physiol. 2015;6. 10.3389/fphys.2015.00295. 10.3389/fphys.2015.00295 PMC4621419 26578968

[22] Bonato M, Villacaro D, Faelli EL, Panascì M, Marmondi F, La Torre A, et al. Training intensity distribution, load management, and performance in recreational long-distance runners. Int J Sports Physiol Perform. Champaign IL, USA: Human Kinetics; 2026;1–9. 10.1123/ijspp.2025-0348. 10.1123/ijspp.2025-0348 41765005

[23] Tschakert G, Hofmann P. High-intensity intermittent exercise: methodological and physiological aspects. Int J Sports Physiol Perform. 2013;8:600–10. 23799827 10.1123/ijspp.8.6.600

[24] Laursen PB, Shing CM, Peake JM, Coombes JS, Jenkins DG. Interval training program optimization in highly trained endurance cyclists. Med Sci Sports Exerc. 2002;34:1801–7. 10.1249/01.MSS.0000036691.95035.7D. 12439086 10.1097/00005768-200211000-00017

[25] Smith T, Coombes J, Geraghty D. Optimising high-intensity treadmill training using the running speed at maximal O(2) uptake and the time for which this can be maintained. Eur J Appl Physiol. 2003;89:337–43. 10.1007/s00421-003-0806-6. 12736843 10.1007/s00421-003-0806-6

[26] Denadai BS, Ortiz MJ, Greco CC, de Mello MT. Interval training at 95% and 100% of the velocity at VO2max: effects on aerobic physiological indexes and running performance. Appl Physiol, Nutr Metab. 2006;31:737–43. 10.1139/h06-080. 17213889 10.1139/h06-080

[27] Schoenmakers PPJM, Hettinga FJ, Reed KE. The moderating role of recovery durations in high-intensity interval-training protocols. Int J Sports Physiol Perform. 2019;14(6):859–67. 10.1123/ijspp.2018-0876. 10.1123/ijspp.2018-0876 31146621

[28] Seiler S, Hetlelid KJ. The impact of rest duration on work intensity and RPE during interval training. Med Sci Sports Exerc. 2005;37:1601–7. http://www.ncbi.nlm.nih.gov/pubmed/16177614. 10.1249/01.mss.0000177560.18014.d8 16177614

[29] Fennell CRJ, Hopker JG. The acute physiological and perceptual effects of individualizing the recovery interval duration based upon the resolution of muscle oxygen consumption during cycling exercise. Int J Sports Physiol Perform. Human Kinetics Publishers Inc.; 2021;16:1580–8. 10.1123/ijspp.2020-0295. 10.1123/ijspp.2020-0295 33848976

[30] Mølmen KS, Rønnestad BR. A narrative review exploring advances in interval training for endurance athletes. Appl Physiol Nutr Metab. 2024;49:1008–13. 10.1139/apnm-2023-0603. Canadian Science Publishing. 38564798 10.1139/apnm-2023-0603

[31] Bossi AH, Mesquida C, Passfield L, Rønnestad BR, Hopker JG. Optimizing interval training through power-output variation within the work intervals. Int J Sports Physiol Perform. 2020;15:982–9. 10.1123/ijspp.2019-0260. Human Kinetics Publishers Inc. 32244222 10.1123/ijspp.2019-0260

[32] Midgley AW, Mc Naughton LR. Time at or near VO2max during continuous and intermittent running. A review with special reference to considerations for the optimisation of training protocols to elicit the longest time at or near VO2max. J Sports Med Phys Fitness. 2006;46:1–14. http://www.ncbi.nlm.nih.gov/pubmed/16596093. 16596093

[33] Demarie S, Koralsztein JP, Billat V. Time limit and time at VO2max’ during a continuous and an intermittent run. J Sports Med Phys Fitness. 2000;40:96–102. http://www.ncbi.nlm.nih.gov/pubmed/11034428. 11034428

[34] Billat VL, Slawinski J, Bocquet V, Demarle A, Lafitte L, Chassaing P, et al. Intermittent runs at the velocity associated with maximal oxygen uptake enables subjects to remain at maximal oxygen uptake for a longer time than intense but submaximal runs. Eur J Appl Physiol. 2000;81:188–96. 10.1007/s004210050029. 10638376 10.1007/s004210050029

[35] Midgley AW, McNaughton LR, Wilkinson M. Is there an optimal training intensity for enhancing the maximal oxygen uptake of distance runners? Empirical research findings, current opinions, physiological rationale and practical recommendations. Sports Med. 2006;36:117–32. 10.2165/00007256-200636020-00003. 16464121 10.2165/00007256-200636020-00003

[36] Helgerud J, Høydal K, Wang E, Karlsen T, Berg P, Bjerkaas M, et al. Aerobic high-intensity intervals improve VO2max more than moderate training. Med Sci Sports Exerc. 2007;39:665–71. 10.1249/mss.0b013e3180304570. 17414804 10.1249/mss.0b013e3180304570

[37] Thevenet D, Tardieu-Berger M, Berthoin S, Prioux J. Influence of recovery mode (passive vs. active) on time spent at maximal oxygen uptake during an intermittent session in young and endurance-trained athletes. Eur J Appl Physiol. 2007;99:133–42. 10.1007/s00421-006-0327-1. 17115178 10.1007/s00421-006-0327-1

[38] Odden I, Nymoen L, Urianstad T, Kristoffersen M, Hammarström D, Hansen J, et al. The higher the fraction of maximal oxygen uptake is during interval training, the greater is the cycling performance gain. Eur J Sport Sci. 2024;24(11):1583–96. 10.1002/EJSC.12202. John Wiley & Sons, Ltd. 39385317 10.1002/ejsc.12202 PMC11534653

[39] Turnes T, de Aguiar RA, Cruz RSdeO, Caputo F. Interval training in the boundaries of severe domain: effects on aerobic parameters. Eur J Appl Physiol. 2016;116:161–9. 10.1007/s00421-015-3263-0. Springer Verlag. 26373721 10.1007/s00421-015-3263-0

[40] Page MJ, McKenzie JE, Bossuyt PM, Boutron I, Hoffmann TC, Mulrow CD, et al. The PRISMA 2020 statement: An updated guideline for reporting systematic reviews. The BMJ. 2021. 10.1136/bmj.n71. 10.1136/bmj.n71 PMC8005924 33782057

[41] Zuniga JM, Berg K, Noble J, Harder J, Chaffin ME, Hanumanthu VS. Physiological responses during interval training with different intensities and duration of exercise. J Strength Cond Res. 2011;25(5):1279–84. 10.1519/JSC.0b013e3181d681b6. 21522072 10.1519/JSC.0b013e3181d681b6

[42] Nicolò A, Bazzucchi I, Lenti M, Haxhi J, Di Palumbo AS, Sacchetti M. Neuromuscular and metabolic responses to high-intensity intermittent cycling protocols with different work-to-rest ratios. Int J Sports Physiol Perform. 2014;9:151–60. 10.1123/IJSPP.2012-0289. 23628754 10.1123/ijspp.2012-0289

[43] Lisbôa FD, Salvador AF, Raimundo JAG, Pereira KL, De Aguiar RA, Caputo F. Decreasing power output increases aerobic contribution during low-volume severe-intensity intermittent exercise. J Strength Cond Res. 2015;29(9):2434–40. 10.1519/JSC.0000000000000914. 10.1519/JSC.0000000000000914 26308828

[44] Zadow EK, Gordon N, Abbiss CR, Peiffer JJ. Pacing, the missing piece of the puzzle to high-intensity interval training. Int J Sports Med. 2015;36:215–9. 10.1055/s-0034-1389973. 25415386 10.1055/s-0034-1389973

[45] Zafeiridis A, Kounoupis A, Dipla K, Kyparos A, Nikolaidis MG, Smilios I, et al. Oxygen delivery and muscle deoxygenation during continuous, long- and short-interval exercise. Int J Sports Med. 2015;36:872–80. 10.1055/s-0035-1554634. 26140688 10.1055/s-0035-1554634

[46] Rønnestad BR, Hansen J. Optimizing interval training at power output associated with peak oxygen uptake in well-trained cyclists. J Strength Cond Res. 2016;30(4):999–1006. 10.1519/JSC.0b013e3182a73e8a. 10.1519/JSC.0b013e3182a73e8a 23942167

[47] Combes A, Dekerle J, Bougault V, Daussin FN. Physiological comparison of intensity-controlled, isocaloric intermittent and continuous exercise†. Eur J Sport Sci. 2018;18:1368–75. 10.1080/17461391.2018.1491627. 29975588 10.1080/17461391.2018.1491627

[48] Rønnestad BR, Moen M, Gunnerød S, Øfsteng S. Adding vibration to high-intensity intervals increase time at high oxygen uptake in well-trained cyclists. Scand J Med Sci Sports. 2018;28:2473–80. 10.1111/sms.13277. 30113750 10.1111/sms.13277

[49] Shi Q, Tong TK, Sun S, Kong Z, Chan CK, Liu W, et al. Influence of recovery duration during 6-s sprint interval exercise on time spent at high rates of oxygen uptake. J Exerc Sci Fit. 2018;16:16–20. 10.1016/j.jesf.2018.01.001. 30662487 10.1016/j.jesf.2018.01.001 PMC6323236

[50] Boynton JR, Danner F, Menaspà P, Peiffer JJ, Abbiss CR. Effect of environmental temperature on high-intensity intervals in well-trained cyclists. Int J Sports Physiol Perform. 2019;14(10):1401–7. 10.1123/ijspp.2018-0689. 10.1123/ijspp.2018-0689 30958046

[51] Lisbôa FD, Raimundo JAG, Salvador AF, Pereira KL, Turnes T, Diefenthaeler F, et al. Acute cardiopulmonary, metabolic, and neuromuscular responses to severe-intensity intermittent exercises. J Strength Cond Res. 2019;33(2):408–16. 10.1519/JSC.0000000000002130. 10.1519/JSC.0000000000002130 28704307

[52] Almquist NW, Nygaard H, Vegge G, Hammarström D, Ellefsen S, Rønnestad BR. Systemic and muscular responses to effort-matched short intervals and long intervals in elite cyclists. Scand J Med Sci Sports. 2020;30:1140–50. 10.1111/sms.13672. 32267032 10.1111/sms.13672

[53] Vaccari F, Giovanelli N, Lazzer S. High-intensity decreasing interval training (HIDIT) increases time above 90% V˙ O2peak. Eur J Appl Physiol. 2020;120:2397–405. 10.1007/s00421-020-04463-w. 32780251 10.1007/s00421-020-04463-w PMC7560936

[54] Agnol CD, Turnes T, Lucas RD. Time spent near V?O2max during different cycling self-paced interval training protocols. Int J Sports Physiol Perform. 2021;16:1347–53. 10.1123/IJSPP.2020-0314. 33771941 10.1123/ijspp.2020-0314

[55] Beltrami FG, Roos E, von Ow M, Spengler CM. Cardiorespiratory responses to constant and varied-load interval training sessions. Int J Sports Physiol Perform. 2021;16:1021–8. 10.1123/ijspp.2020-0104. NLM (Medline). 33607627 10.1123/ijspp.2020-0104

[56] Fennell CRJ, Hopker JG. The acute physiological and perceptual effects of recovery interval intensity during cycling-based high-intensity interval training. Eur J Appl Physiol. 2021;121:425–34. 10.1007/s00421-020-04535-x. Springer Science and Business Media Deutschland GmbH;. 33098020 10.1007/s00421-020-04535-x PMC7862540

[57] Ksoll KSH, Mühlberger A, Stöcker F. Central and peripheral oxygen distribution in two different modes of interval training. Metabolites. MDPI; 2021;11(11):790. 10.3390/metabo11110790. 10.3390/metabo11110790 PMC8623252 34822448

[58] Mckee JR, Wall BA, Peiffer JJ. Temporal location of high-intensity interval training in cycling does not impact the time spent near maximal oxygen consumption. Int J Sports Physiol Perform. 2021;16(7):1029–34. 10.1123/ijspp.2020-0354. 10.1123/ijspp.2020-0354 33691284

[59] Sousa A, Viana JL, Milheiro J, Reis VM, Millet GP. Effect of hypoxia and nitrate supplementation on different high-intensity interval-training sessions. Eur J Appl Physiol. 2021;121:2585–94. 10.1007/s00421-021-04726-0. Springer Science and Business Media Deutschland GmbH;. 34097130 10.1007/s00421-021-04726-0

[60] Balci GA, As H, Ozkaya O, Colakoglu M. Development potentials of commonly used high-intensity training strategies on central and peripheral components of maximal oxygen consumption. Respir Physiol Neurobiol. 2022;302 10.1016/j.resp.2022.103910. 10.1016/j.resp.2022.103910 35405332

[61] Bogdanis GC, Stavrinou PS, Tsirigkakis S, Mougios V, Astorino TA, Mastorakos G. Attenuated metabolic and cardiorespiratory responses to isoenergetic high-intensity interval exercise of short versus long bouts. Med Sci Sports Exerc. 2022;54(7):1199–209. 10.1249/MSS.0000000000002905. 10.1249/MSS.0000000000002905 35234217

[62] Duc S, Urianstad T, Rønnestad BR. Adding vibration during varied-intensity work intervals increases time spent near maximal oxygen uptake in well-trained cyclists. Int J Sports Physiol Perform. 2022;17(11):1565–73. 10.1123/ijspp.2021-0572. 10.1123/ijspp.2021-0572 35926845

[63] Järvinen L, Lundin Petersdotter S, Chaillou T. High-intensity resistance exercise is not as effective as traditional high-intensity interval exercise for increasing the cardiorespiratory response and energy expenditure in recreationally active subjects. Eur J Appl Physiol. 2022;122:459–74. 10.1007/s00421-021-04849-4. Springer Science and Business Media Deutschland GmbH;. 34799752 10.1007/s00421-021-04849-4 PMC8783843

[64] Wommer D, Turnes T, Souza K, Guglielmo LGA. Similar time near VO 2max regardless of work rate manipulation in cycling interval training. Int J Sports Med. 2022;43:350–6. 10.1055/a-1550-9977. Georg Thieme Verlag. 34261134 10.1055/a-1550-9977

[65] Bossi AH, Cole D, Passfield L, Hopker J. Conventional methods to prescribe exercise intensity are ineffective for exhaustive interval training. Eur J Appl Physiol. 2023;123:1655–70. 10.1007/s00421-023-05176-6. Springer Science and Business Media Deutschland GmbH;. 36988672 10.1007/s00421-023-05176-6 PMC10363074

[66] Bossi AH, Mesquida C, Hopker J, Ronnestad BR. Adding intermittent vibration to varied-intensity work intervals: no extra benefit. Int J Sports Med. 2023;44:126–32. 10.1055/a-1812-7600. 35354204 10.1055/a-1812-7600

[67] Miller P, Perez N, Farrell JW. Acute oxygen consumption response to fast start high-intensity intermittent exercise. Sports. Multidisciplinary Digital Publishing Institute (MDPI); 2023;11(12):238. 10.3390/sports11120238. 10.3390/sports11120238 PMC10747366 38133105

[68] Norouzi M, Cabuk R, Balci GA, As H, Ozkaya O. Assessing acute responses to exercises performed within and at the upper boundary of severe exercise domain. Res Q Exerc Sport. 2023;94:1094–100. 10.1080/02701367.2022.2117268. Routledge. 36149826 10.1080/02701367.2022.2117268

[69] Twist C, Bott R, Highton J. The physiological, perceptual and neuromuscular responses of team sport athletes to a running and cycling high intensity interval training session. Eur J Appl Physiol. 2023;123:113–20. 10.1007/s00421-022-05053-8. 36203053 10.1007/s00421-022-05053-8 PMC9813096

[70] Bossi AH, Timmerman W, Cole D, Passfield L, Hopker J. The delta concept does not effectively normalise exercise responses to exhaustive interval training. J Sci Med Sport. Elsevier; 2024;(12):875–82. 10.1016/J.JSAMS.2024.07.019. 10.1016/j.jsams.2024.07.019 39138044

[71] Urianstad T, Hamarsland H, Odden I, Lorentzen HC, Hammarström D, Mølmen KS, et al. The higher oxygen consumption during multiple short intervals is sex-independent and not influenced by skeletal muscle characteristics in well-trained cyclists. Eur J Sport Sci. Eur J Sport Sci; 2024;24(11):1614–26. 10.1002/EJSC.12214. 10.1002/ejsc.12214 PMC11534666 39435498

[72] Appelhans D, Rønnestad B, Skovereng K. Multiple short intervals induce longer time above 90% of maximal oxygen consumption than long intervals when matched by similar fixed intensity, but not during self-paced cycling ergometry. Int J Sports Physiol Perform. 2025;20:848–55. 10.1123/ijspp.2024-0115. 40328438 10.1123/ijspp.2024-0115

[73] Boynton JR, Peiffer JJ, Abbiss CR. Effects of HIIT in cool and hot on temperate performance and physiological response in trained cyclists. J Strength Condition Res 2025;39(3):e485–95. 10.1519/JSC.0000000000005013. 10.1519/JSC.0000000000005013 39977025

[74] Çabuk R, Kayacan Y, Murias JM, Karsten B. Physiologic and mechanical responses to clustered vs. traditional sprint interval exercise approaches. Eur J Appl Physiol. Springer Science and Business Media Deutschland GmbH; 2025;(12):3565–78. 10.1007/s00421-025-05857-4. 10.1007/s00421-025-05857-4 PMC12678567 40581910

[75] Raimundo J, Tramontin A, Martins E, Borszcz F, Turnes T, de Aguiar R, et al. Reliability of time spent near V̇o2max during 2 cycling interval training sessions: the effect of V̇o2max criteria. J Strength Cond Res. 2025;39(9):e1067–e1074. 10.1519/JSC.0000000000005154 40493484

[76] Twist C, Conboy E, Davidson M, Price S, Highton J. Physiological, perceptual and neuromuscular responses of team sport athletes to short duration high intensity interval training using cycling. Eur J Appl Physiol. Springer Science and Business Media Deutschland GmbH; 2025;(10):3009–15. 10.1007/s00421-025-05803-4. 10.1007/s00421-025-05803-4 PMC12479654 40355642

[77] Ducrocq GP, Hureau TJ, Meste O, Blain GM. Similar cardioventilatory but greater neuromuscular stimuli with interval drop jump than with interval running. Int J Sports Physiol Perform. 2020;15(3):330–9. 10.1123/ijspp.2019-0031. 10.1123/ijspp.2019-0031 31188680

[78] Paquette M, Bieuzen F, Billaut F. Sustained muscle deoxygenation vs. Sustained high VO2 During high-intensity interval training in sprint Canoe-Kayak. Front Sports Act Living. Frontiers Media S.A.; 2019;1 10.3389/fspor.2019.00006. 10.3389/fspor.2019.00006 PMC7739754 33344930

[79] Patterson CRM, Neary JP, Wenger HA. Effect of different exercise intervals and work: recovery ratios on oxygen uptake. Sports Med Train Rehabil. 1997;7:185–92. 10.1080/15438629709512081.

[80] Faelli E, Panascì M, Ferrando V, Codella R, Bisio A, Ruggeri P. High-intensity interval training for rowing: acute responses in national-level adolescent males. Int J Environ Res Public Health. MDPI; 2022;19(13):8132. 10.3390/ijerph19138132. 10.3390/ijerph19138132 PMC9265424 35805789

[81] Billat VL, Slawinksi J, Bocquet V, Chassaing P, Demarle A, Koralsztein JP. Very short (15s - 15s) interval-training around the critical velocity allows middle-aged runners to maintain V̇O2 max for 14 minutes. Int J Sports Med. 2001;22:201–8. 10.1055/s-2001-16389. 11354523 10.1055/s-2001-16389

[82] Dupont G, Blondel N, Lensel G, Berthoin S. Critical velocity and time spent at a high level of VO2 for short intermittent runs at supramaximal velocities. Can J Appl Physiol. 2002;27:103–15. http://www.ncbi.nlm.nih.gov/pubmed/12179954. 10.1139/h02-008 12179954

[83] Millet GP, Candau R, Fattori P, Bignet F, Varray A. V̇O2 responses to different intermittent runs at velocity associated with V̇O2max. Can J Appl Physiol. 2003;28:410–23. 10.1139/h03-030. 12955868 10.1139/h03-030

[84] Millet GP, Libicz S, Borrani F, Fattori P, Bignet F, Candau R. Effects of increased intensity of intermittent training in runners with differing V̇O2 kinetics. Eur J Appl Physiol. 2003;90:50–7. 10.1007/s00421-003-0844-0. Springer Verlag. 12811566 10.1007/s00421-003-0844-0

[85] Dupont G, Berthoin S. Time spent at a high percentage of VO2max for short intermittent runs: active versus passive recovery. Can J Appl Physiol. 2004;29 Suppl:S3–16. http://www.ncbi.nlm.nih.gov/pubmed/15602083. 10.1139/h2004-054 15602083

[86] Tardieu-Berger M, Thevenet D, Zouhal H, Prioux J. Effects of active recovery between series on performance during an intermittent exercise model in young endurance athletes. Eur J Appl Physiol. 2004;93:145–52. 10.1007/s00421-004-1189-z. 15549368 10.1007/s00421-004-1189-z

[87] Midgley AW, McNaughton LR, Carroll S. Reproducibility of time at or near V̇O2max during intermittent treadmill running. Int J Sports Med. 2007;28:40–7. 10.1055/s-2006-923856. 16586340 10.1055/s-2006-923856

[88] Midgley AW, McNaughton LR, Carroll S. Time at V̇O2max during intermittent treadmill running: test protocol dependent or methodological artefact? Int J Sports Med. 2007;28:934–9. 10.1055/s-2007-964972. 17497578 10.1055/s-2007-964972

[89] Rozenek R, Funato K, Kubo J, Hoshikawa M, Matsuo A. Physiological responses to interval training sessions at velocities associated with V̇O2max. J Strength Cond Res. 2007;21:188–92. 10.1519/R-19325.1. 17313282 10.1519/R-19325.1

[90] Thevenet D, Leclair E, Tardieu-Berger M, Berthoin S, Regueme S, Prioux J. Influence of recovery intensity on time spent at maximal oxygen uptake during an intermittent session in young, endurance-trained athletes. J Sports Sci. 2008;26:1313–21. 10.1080/02640410802072697. 18821267 10.1080/02640410802072697

[91] Thevenet D, Tardieu M, Zouhal H, Jacob C, Abderrahman BA, Prioux J. Influence of exercise intensity on time spent at high percentage of maximal oxygen uptake during an intermittent session in young endurance-trained athletes. Eur J Appl Physiol. 2007;102:19–26. 10.1007/s00421-007-0540-6. 17851682 10.1007/s00421-007-0540-6

[92] O’Brien BJ, Wibskov J, Knez WL, Paton CD, Harvey JT. The effects of interval-exercise duration and intensity on oxygen consumption during treadmill running. J Sci Med Sport. 2008;11:287–90. 10.1016/j.jsams.2007.05.004. 17584526 10.1016/j.jsams.2007.05.004

[93] Wakefield BR, Glaister M. Influence of work-interval intensity and duration on time spent at a high percentage of VO2max during intermittent supramaximal exercise. J Strength Cond Res. 2009;23:2548–54. 10.1519/JSC.0b013e3181bc19b1. 19910820 10.1519/JSC.0b013e3181bc19b1

[94] Zafeiridis A, Sarivasiliou H, Dipla K, Vrabas IS. The effects of heavy continuous versus long and short intermittent aerobic exercise protocols on oxygen consumption, heart rate, and lactate responses in adolescents. Eur J Appl Physiol. 2010;110:17–26. 10.1007/s00421-010-1467-x. 20383773 10.1007/s00421-010-1467-x

[95] Zafeiridis A, Rizos S, Sarivasiliou H, Kazias A, Dipla K, Vrabas IS. The extent of aerobic system activation during continuous and interval exercise protocols in young adolescents and men. Appl Physiol Nutr Metab. 2011;36(1):128–36. 10.1139/H10-096. 10.1139/H10-096 21326387

[96] Buchheit M, Kuitunen S, Voss SC, Williams BK, Mendez-Villanueva A, Bourdon PC. Physiological strain associated with high-intensity hypoxic intervals in highly trained young runners. J Strength Cond Res. 2012;26(1):94–105. 10.1519/JSC.0b013e3182184fcb. 10.1519/JSC.0b013e3182184fcb 22158261

[97] Ben Abderrahman A, Zouhal H, Chamari K, Thevenet D, De Mullenheim PY, Gastinger S, et al. Effects of recovery mode (active vs. passive) on performance during a short high-intensity interval training program: a longitudinal study. Eur J Appl Physiol. 2013;113:1373–83. 10.1007/s00421-012-2556-9. 23229881 10.1007/s00421-012-2556-9

[98] De Aguiar RA, Turnes T, De Oliveira Cruz RS, Caputo F. Fast-start strategy increases the time spent above 95 %VO2max during severe-intensity intermittent running exercise. Eur J Appl Physiol. 2013;113:941–9. 10.1007/s00421-012-2508-4. 23053127 10.1007/s00421-012-2508-4

[99] Panissa VLG, Julio UF, Pinto-E-Silva CM, Andreato L V., Schwartz J, Franchini E. Influence of the aerobic fitness on time spent at high percentage of maximal oxygen uptake during a high-intensity intermittent running. Journal of Sports Medicine and Physical Fitness. 2014;54(6)708–14. 25350028

[100] Panascì M, Lepers R, La Torre A, Bonato M, Assadi H. Physiological responses during intermittent running exercise differ between outdoor and treadmill running. Appl Physiol Nutr Metab. 2017;42:973–7. 10.1139/apnm-2017-0132. 28549220 10.1139/apnm-2017-0132

[101] Smilios I, Myrkos A, Zafeiridis A, Toubekis A, Spassis A, Tokmakidis SP. The effects of recovery duration during high-intensity interval exercise on time spent at high rates of oxygen consumption, oxygen kinetics and blood lactate. J Strength Cond Res. 2018;(8)2183–9. 10.1519/JSC.0000000000001904. 10.1519/JSC.0000000000001904 28301436

[102] Baquet G, Dupont G, Gamelin FX, Aucouturier J, Berthoin S. Active versus passive recovery in high-intensity intermittent exercises in children: an exploratory study. Pediatr Exerc Sci. 2019;31(2):248–53. 10.1123/pes.2018-0218. 10.1123/pes.2018-0218 30907283

[103] Schoenmakers PPJM, Reed KE. The effects of recovery duration on physiological and perceptual responses of trained runners during four self-paced HIIT sessions. J Sci Med Sport. 2019;22:462–6. 10.1016/j.jsams.2018.09.230. Elsevier Ltd. 30297216 10.1016/j.jsams.2018.09.230

[104] Myrkos A, Smilios I, Zafeiridis A, Iliopoulos S, Kokkinou EM, Douda H, et al. Effects of work and recovery duration and their ratio on cardiorespiratory and metabolic responses during aerobic interval exercise. J Strength Cond Res. 2022;36(8):2169–75. 10.1519/JSC.0000000000003578. 10.1519/JSC.0000000000003578 32379235

[105] José Arantes F, Freitas Vieira P, Licnerski Borges D, Balbino Lizardo F, Elias Dias Nunes J, Alves Pereira A. Effect of different work and recovery settings during high-intensity intermittent training on maximal oxygen uptake and session volume responses. Sci Sports. 2021;36:415.e1-415.e7. 10.1016/j.scispo.2020.08.002.

[106] Germano MD, Sindorf MAG, Crisp AH, Braz T V., Brigatto FA, Nunes AG, et al. Effect of different recoveries during HIIT sessions on metabolic and cardiorespiratory responses and sprint performance in healthy men. J Strength Cond Res. 2022;36(1):121–9. 10.1519/JSC.0000000000003423. 10.1519/JSC.0000000000003423 31895286

[107] Sánchez-Otero T, Tuimil JL, Boullosa D, Varela-Sanz A, Iglesias-Soler E. Active vs. passive recovery during an aerobic interval training session in well-trained runners. Eur J Appl Physiol. 2022;122:1281–91. 10.1007/s00421-022-04926-2. 35262762 10.1007/s00421-022-04926-2 PMC9012711

[108] Bok D, Gulin J, Škegro D, Šalaj S, Foster C. Comparison of anaerobic speed reserve and maximal aerobic speed methods to prescribe short format high-intensity interval training. Scand J Med Sci Sports. 2023;33:1638–47. 10.1111/sms.14411. 37246423 10.1111/sms.14411

[109] Held S, Rappelt L, Giesen R, Wiedenmann T, Deutsch JP, Wicker P, et al. Increased oxygen uptake in well-trained runners during uphill high intensity running intervals: A randomized crossover testing. Front Physiol. Frontiers Media S.A.; 2023;14 10.3389/fphys.2023.1117314. 10.3389/fphys.2023.1117314 PMC9977817 36875023

[110] Varela-Sanz A, Sánchez-Otero T, Tuimil JL, Boullosa D, Iglesias-Soler E. Influence of recovery mode on the maximum number of intervals until exhaustion during an aerobic interval training session. J Strength Cond Res. 2023;37(9):e510–20. 10.1519/JSC.0000000000004463. 10.1519/JSC.0000000000004463 36723090

[111] Vaccari F, Stafuzza J, Giovanelli N, Lazzer S. High-intensity interval training: optimizing oxygen consumption and time to exhaustion taking advantage of the exponential reconstitution behaviour of D’. Eur J Appl Physiol. 2023;123:201–9. 10.1007/s00421-022-05059-2. Springer Science and Business Media Deutschland GmbH. 36242642 10.1007/s00421-022-05059-2 PMC9813124

[112] Fleckenstein D, Braunstein H, Walter N. Faster intervals, faster recoveries - intensified short VO2max running intervals are inferior to traditional long intervals in terms of time spent above 90% VO2max. Front Sports Act Living. Front Sports Act Living; 2025;6. 10.3389/FSPOR.2024.1507957. 10.3389/fspor.2024.1507957 PMC11743937 39835194

[113] Bentley DJ, Roels B, Hellard P, Fauquet C, Libicz S, Millet GP. Physiological responses during submaximal interval swimming training: effects of interval duration. J Sci Med Sport. 2005;8:392–402. 10.1016/S1440-2440(05)80054-4. 16602167 10.1016/s1440-2440(05)80054-4

[114] Libicz S, Roels B, Millet GP. Responses to intermittent swimming sets at velocity associated with max V O 2 responses to intermittent . swimming sets at velocity associated with V O 2 max. Can J Appl Physiol 2014;30(5):543–53. 10.1139/h05-140 16293903

[115] Almeida TAF, Pessôa Filho DM, Espada MC, Reis JF, Sancassani A, Massini DA, et al. Physiological responses during high-intensity interval training in young swimmers. Front Physiol. Frontiers Media S.A.; 2021;12. 10.3389/fphys.2021.662029. 10.3389/fphys.2021.662029 PMC8281220 34276394

[116] Almeida TAF, Massini DA, Silva Júnior OT, Venditti Júnior R, Espada MAC, Macedo AG, et al. Time limit and V̇O2 kinetics at maximal aerobic velocity: Continuous vs. intermittent swimming trials. Front Physiol. Frontiers Media S.A.; 2022;13. 10.3389/fphys.2022.982874. 10.3389/fphys.2022.982874 PMC9562734 36246138

[117] Rønnestad BR, Rømer T, Hansen J. Increasing oxygen uptake in well-trained cross-country skiers during work intervals with a fast start. Int J Sports Physiol Perform. 2020;15:383–9. 10.1123/ijspp.2018-0360. Human Kinetics Publishers Inc. 31621643 10.1123/ijspp.2018-0360

[118] Rønnestad BR, Bakken TA, Thyli V, Hansen J, Ellefsen S, Hammarstrøm D. Increasing oxygen uptake in cross-country skiers by speed variation in work intervals. Int J Sports Physiol Perform. 2022;17:384–90. 10.1123/ijspp.2021-0226. Human Kinetics Publishers Inc.;. 34814113 10.1123/ijspp.2021-0226

[119] Rønnestad BR, Bjerkrheim KA, Hansen J, Mølmen KS. A 6-day high-intensity interval microcycle improves indicators of endurance performance in elite cross-country skiers. Front Sports Act Living. 2022;4. 10.3389/fspor.2022.948127. 10.3389/fspor.2022.948127 PMC9682127 36439620

[120] Higgins JPT, Thomas J, Chandler J, Cumpston M, Li T, Page MJ, et al. Cochrane handbook for systematic reviews of interventions. Cochrane Handbook for Syst Rev Intervent 2019. 10.1002/9781119536604. 10.1002/14651858.ED000142 PMC10284251 31643080

[121] Maher CG, Sherrington C, Herbert RD, Moseley AM, Elkins M. Reliability of the PEDro scale for rating quality of randomized controlled trials. Phys Ther. 2003;83(8):713–21. 10.1093/ptj/83.8.713. 12882612

[122] Sterne JAC, Savović J, Page MJ, Elbers RG, Blencowe NS, Boutron I, et al. RoB 2: A revised tool for assessing risk of bias in randomised trials. The BMJ. 2019;366. 10.1136/bmj.l4898. 10.1136/bmj.l4898 31462531

[123] McGuinness LA, Higgins JPT. Risk-of-bias VISualization (robvis): An R package and Shiny web app for visualizing risk-of-bias assessments. Res Synth Methods. John Wiley and Sons Ltd; 2021: 55–61. 10.1002/jrsm.1411. 10.1002/jrsm.1411 32336025

[124] Abderrahman A Ben, Prioux J, Chamari K, Ounis O Ben, Tabka Z, Zouhal H. Running interval training and estimated plasma-volume variation. Int J Sports Physiol Perform. 2013;8:358–65. 23113934 10.1123/ijspp.8.4.358

[125] Higgins JPT, Thompson SG, Deeks JJ, Altman DG. Measuring inconsistency in meta-analyses. Br. Med. J. 2003 10.1136/bmj.327.7414.557. 10.1136/bmj.327.7414.557 PMC192859 12958120

[126] Deeks JJ, Higgins JP, Altman DG. Chapter 10: analysing data and undertaking meta‐analyses. In: Cochrane handbook for systematic reviews of interventions, 2019; 2.

[127] Hopkins WG, Marshall SW, Batterham AM, Hanin J. Progressive statistics for studies in sports medicine and exercise science. Med. Sci. Sports Exerc. 2009. pp. 3–12. 10.1249/MSS.0b013e31818cb278. 10.1249/MSS.0b013e31818cb278 19092709

[128] Van Hooren B, Souren T, Bongers BC. Accuracy of respiratory gas variables, substrate, and energy use from 15 CPET systems during simulated and human exercise. Scand J Med Sci Sports. 2024;34(1):e14490. 10.1111/sms.14490. 10.1111/sms.14490 37697640

[129] Coates AM, Joyner MJ, Little JP, Jones AM, Gibala MJ. A Perspective on high-intensity interval training for performance and health. Sports Med 2023;53(1):85–96. 10.1007/s40279-023-01938-6. 10.1007/s40279-023-01938-6 PMC10721680 37804419

[130] Goulding RP, Roche DM, Marwood S. Elevated baseline work rate slows pulmonary oxygen uptake kinetics and decreases critical power during upright cycle exercise. Physiol Rep. 2018;6(14):e13802. 10.14814/phy2.13802. 10.14814/phy2.13802 PMC6056736 30039557

[131] Goulding RP, Burnley M, Wüst RCI. How priming exercise affects oxygen uptake kinetics: from underpinning mechanisms to endurance performance. Sports Med 2023;(5):959–76. 10.1007/s40279-023-01832-1. 10.1007/s40279-023-01832-1 PMC10115720 37010782

[132] Berger NJA, Jones AM. Pulmonary O2 uptake on-kinetics in sprint- and endurance-trained athletes. Appl Physiol Nutr Metab 2007;32(3):383–93. 10.1139/H06-109. 10.1139/H06-109 17510672

[133] Bailey SJ, Vanhatalo A, Dimenna FJ, Wilkerson DP, Jones AM. Fast-start strategy improves V̇O2 kinetics and high-intensity exercise performance. Med Sci Sports Exerc. 2011;43:457–67. 10.1249/MSS.0B013E3181EF3DCE. 20689463 10.1249/MSS.0b013e3181ef3dce

[134] Stöggl TL, Strepp T, Wiesinger HP, Haller N. A training goal-oriented categorization model of high-intensity interval training. Front. Physiol. Frontiers Media SA; 2024 10.3389/fphys.2024.1414307. 10.3389/fphys.2024.1414307 PMC11218030 38957216

[135] Weir JP. Quantifying test-retest reliability using the intraclass correlation coefficient and the SEM. J. Strength Cond. Res. 2005;(1):231–40. 10.1519/15184.1. 10.1519/15184.1 15705040

[136] Midgley SW, Mc Naughton LR, Wilkinson M. Criteria and other methodological considerations in the evaluation of time at V̇O2max. J Sports Med Phys Fitness. 2006;46(2):183–8. 16823345

[137] McCrudden MC, Keir DA, Belfry GR. The effects of short work vs. longer work periods within intermittent exercise on VO2p kinetics, muscle deoxygenation, and energy system contribution. J Appl Physiol. 2017;122(6):1435–44. 10.1152/japplphysiol.00514.2016. 10.1152/japplphysiol.00514.2016 PMC5494429 28336535

[138] Benson AP, Bowen TS, Ferguson C, Murgatroyd SR, Rossiter HB. Data collection, handling, and fitting strategies to optimize accuracy and precision of oxygen uptake kinetics estimation from breath-by-breath measurements. J Appl Physiol. 2017;123(1):227–42. 10.1152/japplphysiol.00988.2016. 10.1152/japplphysiol.00988.2016 28450551

[139] Rønnestad BR, Hansen J, Vegge G, Tønnessen E, Slettaløkken G. Short intervals induce superior training adaptations compared with long intervals in cyclists - an effort-matched approach. Scand J Med Sci Sports. 2015;25(2):143–51. 10.1111/sms.12165. 24382021 10.1111/sms.12165

[140] Trinity JD, Lee JF, Pahnke MD, Beck KC, Coyle EF. Attenuated relationship between cardiac output and oxygen uptake during high-intensity exercise. Acta Physiologica. 2012;204(3):362–70. 10.1111/j.1748-1716.2011.02341.x. 10.1111/j.1748-1716.2011.02341.x 21791015

