# Supplementary material for: Time spent at or near V̇O₂max during high-intensity interval training - a systematic review and meta-analysis
Source: BMC Sports Sci Med Rehabil. 2026 Jun 3;18:358. doi: 10.1186/s13102-026-01766-x (PMC13480085; doi:10.1186/s13102-026-01766-x)
Supplement: Supplementary file 1 [file 13102_2026_1766_MOESM1_ESM.docx]

| **Study** | **Protocol Information** | **Calculation explained:** |
| --- | --- | --- |
| Zuniga et al. [41] | Calculated | Total training duration was reported. Work interval duration was calculated from the reported work and recovery durations.  In the 30:30‑s protocol, mean total time to exhaustion was reported as 25.5 ± 5.3 min (= 1530 ± 318 s). Given a 1:1 work‑to‑recovery ratio, work interval duration was calculated as 765 ± 159 s |
| Nicolò et al. [42] | Calculated | Total training duration was reported. Work interval duration was calculated from the reported work and recovery durations.  In the 30:30‑s protocol, mean time to exhaustion was reported as 38 ± 13 min (= 2280 ± 780 s). Given a 1:1 work‑to‑recovery ratio, work interval duration was calculated as 1140 ± 390 s |
| Lisbôa et al. [43] | Calculated | Total work interval duration was reported. Using the reported interval structure, total training duration was calculated.  In the constant power output protocol, participants completed 2.9 ± 0.6 repetitions (= 225 ± 33 s). Given a 1:1 work‑to‑recovery ratio, total training duration was calculated as 450 ± 66 s |
| Zadow et al. [44] | Reported | No additional calculations were required |
| Zafeiridis et al. [45] | Calculated | Total work interval duration was reported. Total training duration was calculated from the reported work and recovery durations, and completed number of intervals.  In the 30:30‑s protocol, participants completed 29 ± 5 repetitions (= 870 ± 150 s). Given a 1:1 work‑to‑recovery ratio, total training duration was calculated as 1740 ± 150 s |
| Rønnestad & Hansen [46] | Calculated | Total training duration was reported. Work interval duration was calculated from the reported work and recovery durations.  In the 30:15‑s protocol, mean time to exhaustion was reported as 1385 ± 547 s. Given a 2:1 work‑to‑recovery ratio, work interval duration was calculated as 923 ± 365 s |
| Turnes et al. [39] | Reported | No additional calculations were required. |
| Combes et al. [47] | Reported | No additional calculations were required. |
| Rønnestad et al. [48] | Reported | No additional calculations were required. |
| Shi et al. [49] | Reported | No additional calculations were required. |
| Boynton et al. [50] | Reported | No additional calculations were required. |
| Lisbôa et al. [51] | Calculated | Total work interval duration was reported. Using the reported interval structure, total training duration was calculated.  In the constant power output protocol, participants completed 3.7 ± 0.8 repetitions (= 274 ± 65 s). Given a 1:1 work‑to‑recovery ratio, total training duration was calculated as 1265 ± 446 |
| Almquist et al. [52] | Reported | No additional calculations were required |
| Bossi et al. [31] | Reported | No additional calculations were required |
| Vaccari et al. [53] | Calculated | Total training duration was reported. Work interval duration was calculated from the reported work and recovery durations.  In the 30:20-s protocol, mean time to exhaustion was reported as 714 ± 265 s. Given a 3:2 work‑to‑recovery ratio, work interval duration was calculated as 478 ± 178 s |
| Dall’Agnol et al. [54] | Reported | No additional calculations were required |
| Beltrami et al. [55] | Reported | No additional calculations were required |
| Fennell & Hopker [56] | Reported | No additional calculations were required |
| Fennell & Hopker [29] | Reported | No additional calculations were required |
| Ksoll et al. [57] | Reported | No additional calculations were required |
| McKee et al. [58] | Reported | No additional calculations were required |
| Sousa et al. [59] | Reported | No additional calculations were required |
| Balci et al. [60] | Reported | No additional calculations were required |
| Bogdanis et al. [61] | Reported | No additional calculations were required |
| Duc et al. [62] | Reported | No additional calculations were required |
| Järvinen et al. [63] | Reported | No additional calculations were required |
| Wommer et al. [64] | Reported | No additional calculations were required |
| Bossi et al. [65] | Reported | No additional calculations were required |
| Bossi et al. [66] | Clarified in personal communication | No additional calculations were required |
| Miller et al. [67] | Reported | No additional calculations were required |
| Norouzi et al. [68] | Reported | No additional calculations were required |
| Twist et al. [69] | Reported | No additional calculations were required |
| Bossi et al. [70] | Clarified in personal communication | No additional calculations were required |
| Odden et al. [38] | Reported | No additional calculations were required |
| Urianstad et al. [71] | Reported | No additional calculations were required |
| Appelhans et al. [72] | Reported | No additional calculations were required |
| Boynton et al. [73] | Reported | No additional calculations were required |
| Çabuk et al. [74] | Reported | No additional calculations were required |
| Raimundo et al. [75] | Reported | No additional calculations were required |
| Twist et al. [76] | Reported | No additional calculations were required |
| **Jumping (and Running)** |  |  |
| Ducrocq et al. [77] | Reported | No additional calculations were required |
| **Kayaking** |  |  |
| Paquette et al. [78] | Reported | No additional calculations were required |
| **Rowing** |  |  |
| Patterson et al. [79] | Reported | No additional calculations were required |
| Faelli et al. [80] | Reported | No additional calculations were required |
| **Running** |  |  |
| Billat et al. [34] | Calculated | The number of completed intervals was reported. Using the reported interval structure, total training duration and work interval duration were calculated.  The mean number of intervals completed was reported as 19 ± 6 (= 570 ± 180 s). Given a 1:1 work‑to‑recovery ratio, total training duration was calculated as 1140 ± 360 s |
| Demarie et al. [33] | Calculated | Total work interval duration was reported. Using the reported interval structure, total training duration was calculated.  Mean time to exhaustion was reported as 19:38 ± 5:10 min:s (= 1178 ± 310 s). Given a 2:1 work‑to‑recovery ratio, total training duration was calculated as 1766 ± 465 s |
| Billat et al. [81] | Calculated | The number of completed intervals was reported. Using the reported interval structure, total training- and work interval duration were calculated.  In the 90% vV̇O₂max condition, 42 ± 12 intervals were completed (630 ± 180 s). Given a 1:1 work‑to‑recovery ratio, total training duration was calculated as 1260 ± 360 s |
| Dupont et al. [82] | Calculated | Total work interval duration was reported. Using the reported interval structure, total training duration was calculated.  In the 110% MAV protocol, mean time to exhaustion was reported as 698 ± 355 s. Given a 1:1 work‑to‑recovery ratio, total training duration was calculated as 1396 ± 710 s |
| Millet et al. [83] | Calculated | Total work interval durations were calculated from reported time to exhaustion of 236 ± 49 s. Using the reported interval structure, total training duration was calculated.  In the 30:30-s protocol, work interval duration was specified as 707 ± 147 s. time Given a 1:1 work‑to‑recovery ratio, work interval duration was calculated as 1414 ± 294 s |
| Millet et al. [84] | Calculated | Total work interval durations were calculated from reported time to exhaustion of 243.7 ± 39.5 s. Using the reported interval structure, total training duration and work interval duration were calculated.  In the vV̇O₂max protocol, total time work interval duration was reported as 731 ± 121 s. Given a 1:1 work‑to‑recovery ratio, total training duration was calculated as 1451 ± 237 s |
| Dupont & Berthoin [85] | Calculated | Total training duration was reported. Work interval duration was calculated from the reported work and recovery durations.    In the in protocol with active recovery, mean time to exhaustion was reported as 445 ± 79 s. Given a 1:1 work‑to‑recovery ratio, work interval duration was calculated as 228 ± 40 s |
| Tardieu-Berger et al. [86] | Calculated | Total training duration was reported. Work interval duration was calculated from the reported work and recovery durations.  In the 30:30-s protocol, mean time to exhaustion was reported as 622 ± 186 s. Given the reported interval structure and a 1:1 work‑to‑recovery ratio, work interval duration was calculated as 311 ± 93 |
| Midgley et al. [87] | Calculated | Total training duration was reported. Work interval duration was calculated from the reported work and recovery durations.  In the initial visit, mean time to exhaustion was reported as 831 ± 238 s. Using the reported interval durations and a 1:1 work‑to‑recovery ratio, work interval duration was calculated as 416 ± 119 s |
| Midgley et al. [88] | Calculated | Total training duration was reported. Work interval duration was calculated from the reported work and recovery durations.  Mean time to exhaustion was reported as 18.5 ± 7.8 min (= 1110 ± 468 s). Given the reported interval structure and a 1:1 work‑to‑recovery ratio, work interval duration was calculated as 555 ± 234 s |
| Rozenek et al. [89] | Calculated | The number of completed intervals was reported. Using the reported interval structure, total training- and work interval duration were calculated.  In the 15:15-s protocol, 35.3 ± 2.3 intervals were completed (= 1059 ± 35 s). Given the reported interval structure and a 1:1 work‑to‑recovery ratio, work interval duration was calculated as 530 ± 17 s |
| Thevenet et al. [37], and Thevenet et al. [90] | Calculated | Total training duration was reported. Work interval duration was calculated from the reported work and recovery durations.  In the 50% MAV active recovery protocol, total training time was reported as 1072 ± 388 s. Using the reported 1:1 work‑to‑recovery ratio, work‑interval time was calculated as 536 ± 194 s |
| Thevenet et al. [91] | Calculated | Total training duration was reported. Work interval duration was calculated from the reported work and recovery durations.  In 100% MAV protocol, total training time was reported as 1440 ± 169 s. Using the reported 1:1 work‑to‑recovery ratio, work‑interval time was calculated as 720 ± 84 s |
| O’Brien et al. [92] | Reported | No additional calculations were required |
| Wakefield & Glaister [93] | Calculated | Total training duration was reported Work interval duration was calculated from the reported work and recovery durations.  In the 20:20-s protocol, mean time to exhaustion was reported as 54.9 ± 7.1 min (= 3294 ± 426 s). Given the reported interval structure and a 1:1 work‑to‑recovery ratio, work interval duration was calculated as 1640 ± 220 s |
| Zafeiridis et al. [94] | Calculated | Total training duration was reported. Work interval duration was calculated from the reported work and recovery durations.  In the 30:30-s protocol, mean time to exhaustion was reported as 930 ± 462 s. Given the reported interval structure and a 1:1 work‑to‑recovery ratio, work interval duration was calculated as 480 ± 231 s |
| Zafeiridis et al. [95] | Calculated | Total work interval duration was reported. Using the reported interval structure, total training duration was calculated.  In the 30 :30-s protocol of the young adults, mean time to exhaustion was reported as 381 ± 65 s. Using the reported interval duration and a 1:1 work‑to‑recovery ratio, total training duration was calculated as 708 ± 154 s |
| Buchheit et al. [96] | Reported | No additional calculations were required |
| Abderrahman et al. [97] | Calculated | Total training duration was reported. Work interval duration was calculated from the reported work and recovery durations.  In the initial 30:30-s protocol, mean time to exhaustion was reported as 1883 ± 788 s. Using the reported interval duration and a 1:1 work‑to‑recovery ratio, work interval duration was calculated as 942 ± 394 s. |
| De Aguiar et al. [98] | Calculated | Total work interval duration was reported. Using the reported interval structure, total training duration was calculated.  In the 30:15-s intermittent protocol at 105% critical velocity, total time run at vV̇O₂max was reported as 1439 ± 518 s. Using the reported interval duration and a 2:1 work‑to‑recovery ratio, total training duration was calculated as 2159 ± 689 s |
| Panissa et al. [99] | Calculated | The number of intervals was calculated from the reported running speed and the fixed total distance (4 km). Using the reported interval structure, total training duration was subsequently calculated.  In the moderate group, total time run at Vmas was calculated as 809 ± 87 s. Using the reported interval duration and a 1:1 work‑to‑recovery ratio, total training duration was calculated as 1618 ± 174 s |
| Panasci et al. [100] | Reported | No additional calculations were required |
| Smilios et al. [101] | Reported | No additional calculations were required |
| Baquet et al. [102] | Calculated | Total work interval duration and number of completed intervals was reported. Using the reported interval durations, total training duration was calculated.  In the active recovery protocol, work interval time was reported as 201 ± 92 s (= 13.3 ± 6.1 completed intervals). Using the reported interval duration and a 1:1 work‑to‑recovery ratio, total training duration was calculated as 402 ± 184 s |
| Schoenmakers & Reed [103] | Clarified in personal communication | No additional calculations were required |
| Myrkos et al. [104] | Calculated | Total training duration was reported. Work interval duration was calculated from the reported work and recovery durations.  In the 60:30-s protocol, total training duration was reported as 17.75 ± 5.53 min (= 1065 ± 332 s). Using the reported interval duration and a 2:1 work‑to‑recovery ratio, work interval time was calculated as 713 ± 222 s |
| Beltrami et al. [55] | Reported | No additional calculations were required |
| José Arantes et al. [105] | Reported | No additional calculations were required |
| Germano et al. [106] | Calculated | Total work interval duration was reported in a figure. Using the reported interval structure, total training duration was calculated.  In the 2-min active recovery protocol, total training duration was reported as 10 ± 1.25 min (=600 ± 87 s). Using the reported interval duration and a 1:1 work‑to‑recovery ratio, total training duration was calculated as 1200 ± 174 s. |
| Sánchez-Otero et al. [107] | Reported | No additional calculations were required |
| Bok et al. [108] | Reported | No additional calculations were required |
| Held et al. [109] | Reported | No additional calculations were required |
| Twist et al. [69] | Reported | No additional calculations were required |
| Varela-Sanz et al. [110] | Calculated | Total work interval duration was reported. Using the reported interval structure, total training duration was calculated.  In the active recovery protocol, work interval duration was reported as 1395 ± 458 s. Using the reported interval structure and a 1:1 work‑to‑recovery ratio, total training duration was calculated as 2670 ± 916 s |
| Vaccari et al. [111] | Calculated | Total training duration was reported. Work interval duration was calculated from the reported work and recovery durations.  In the 30:20-s intermittent protocol, total training time was reported as 678 ± 116 s. Using the reported interval duration and a 3:2 work‑to‑recovery ratio, work interval time was calculated as 407 ± 70 s |
| Fleckenstein et al. [112] | Reported | No additional calculations were required |
| **Swimming** |  |  |
| Bentley et al. [113] | Calculated | Interval duration was calculated from the reported swimming speed and distance. Using the reported interval structure, total training duration was subsequently calculated.  In the 16×100 m protocol, work interval time was calculated as 1194 ± 54 s. Using the reported interval structure and a 5:1 work‑to‑recovery ratio, total training time was determined as 1434 ± 64 s |
| Libicz et al. [114] | Calculated | Interval duration was calculated from the reported swimming speed and distance. Using the reported interval structure, total training duration was subsequently calculated.  In the 8×100 m protocol, work interval time was calculated as 640 ± 36 s. Using the reported interval structure and the associated recovery periods, total training time was calculated as 880 ± 47 s |
| Almeida et al. [115] | Calculated | Total training duration was reported. Work interval duration was calculated from the reported work and recovery durations.  In the 8×100 m protocol, total training time was reported as 750 ± 60 s. Using the reported interval structure, work interval time was calculated as 630 ± 45 s |
| Almeida et al. [116] | Calculated | Total training duration was reported. Work interval duration was calculated from the reported work and recovery durations.  The total training time was reported as 1206 ± 591 s. Using the reported interval structure, work interval time was calculated as 1014 ± 497 s |
| **Cross Country Skiing** |  |  |
| Rønnestad et al. [117] | Reported | No additional calculations were required |
| Rønnestad et al. [118] | Reported | No additional calculations were required |
| Rønnestad et al. ­­[119] | Reported | No additional calculations were required |
