# Supplementary material for: Time spent at or near V̇O₂max during high-intensity interval training - a systematic review and meta-analysis
Source: BMC Sports Sci Med Rehabil. 2026 Jun 3;18:358. doi: 10.1186/s13102-026-01766-x (PMC13480085; doi:10.1186/s13102-026-01766-x)
Supplement: Supplementary file 2 [file 13102_2026_1766_MOESM2_ESM.docx]

|  |  | Time (in sec) above | | | | |
| --- | --- | --- | --- | --- | --- | --- |
|  | **HIIT Protocol – Work Interval** | **80% V̇O_2_max** | **85% V̇O_2_max** | **90% V̇O_2_max** | **95% V̇O_2_max** | **V̇O_2_max** |
| **Cycling** |  |  |  |  |  |  |
| Zuniga et al. [41] ^A^ | X * 30 s at 90% MAP |  |  |  | 384 ± 468 |  |
|  | X * 3 min at 90% MAP |  |  |  | 270 ± 192 |  |
|  | X * 30 s at MAP |  |  |  | 348 ± 348 |  |
|  | X * 3 min at MAP |  |  |  | 276 ± 144 |  |
| Nicolò et al. [42] | X * 30 s |  |  | 259 ± 226 | 91 ± 138 |  |
|  | X * 40 s |  |  | 401 ± 191 | 301 ± 169 |  |
| Lisbôa et al. [43] | X * 60% Tlow |  |  | 123 ± 48 | 76 ± 49 |  |
|  | X * Tlow |  |  | 259 ± 75 | 186 ± 72 |  |
| Zadow et al. [44] ^B^ | 3 * 3 min ‘*All Out’* |  | 454 ± 30 |  |  |  |
|  | 3 * 3 min *CPO* |  | 374 ± 60 |  |  |  |
|  | 3 * 3 min *A-CPO, Isoeffort Max* |  | 410 ± 49 |  |  |  |
| Zafeiridis et al. [45] | X * 30 s | 162 ± 174 |  |  |  |  |
|  | X * 2 min | 445 ± 235 |  |  |  |  |
| Rønnestad & Hansen [46] | X * 30 s |  |  | 678 ± 270 |  |  |
|  | X * 50% Tmax |  |  | 397 ± 156 |  |  |
|  | X * 80% Tmax, |  |  | 366 ± 127 |  |  |
| Turnes et al. [39] | 4 * 5 min |  |  | 19 ± 19 |  |  |
|  | 7 * 5 min |  |  | 26 ± 39 |  |  |
|  | 2 * (4 * 80 s) |  |  | 115 ± 122 |  |  |
|  | 2 * (7* 80 s) |  |  | 127 ± 113 |  |  |
| Combes et al. [47] | 30 * 1 min |  |  | 356 ± 301 |  |  |
| Rønnestad et al. [48] | 6 * 5 min |  |  | 417 ± 317 |  |  |
|  | 6 * 5 min (+ vibration) |  |  | 659 ± 420 |  |  |
| Shi et al. [49] | 40 * 6 s (15 s recovery) | 557 ± 249 | 470 ± 256 | 368 ± 227 | 271 ± 206 | 179 ± 177 |
|  | 40 * 6 s (30 s recovery) | 317 ± 238 | 188 ± 144 | 118 ± 108 | 79 ± 87 | 40 ± 58 |
|  | 40 * 6 s (1 min recovery) | 47 ± 79 | 30 ± 69 | 20 ± 50 | 12 ± 36 | 8 ± 25 |
|  | 8 * 30 s | 151 ± 90 | 109 ± 72 | 74 ± 58 | 43 ± 36 | 18 ± 25 |
| Boynton et al. [50] | 5 * 4 min in 5° |  |  | 184 ± 210 |  |  |
|  | 5 * 4 min in 13° |  |  | 179 ± 150 |  |  |
|  | 5 * 4 min in 22° |  |  | 260 ± 194 |  |  |
|  | 5 * 4 min in 35° |  |  | 418 ± 270 |  |  |
| Lisbôa et al. [51] | X * 60% T_max_ |  |  |  | 98 ± 65 |  |
|  | X * T_max_, |  |  |  | 323 ± 227 |  |
| Almquist et al. [52] ^B^ | 3 * (13 * 30 s) |  |  | 848 ± 351 |  |  |
|  | 4 * 5 min |  |  | 595 ± 200 |  |  |
| Bossi et al. [31] | 6 * 5 min *CPO* |  |  | 286 ± 162 |  |  |
|  | 6 * 5 min *VPO* |  |  | 410 ± 207 |  |  |
| Vaccari et al. [53] | X * 30 s |  |  | 183 ± 225 |  |  |
|  | X * 3 min |  |  | 179 ± 145 |  |  |
|  | X * *HIDIT* |  |  | 312 ± 207 |  |  |
| Dall’Agnol et al. [54] | 4 * 4 min (1 min recovery) |  |  | 285 ± 242 | 76 ± 143 |  |
|  | 4 * 4 min (2 min recovery) |  |  | 458 ± 258 | 225 ± 218 |  |
|  | 4 * 8 min (2 min recovery) |  |  | 365 ± 427 | 60 ± 122 |  |
|  | 4 * 8 min (4 min recovery) |  |  | 687 ± 598 | 317 ± 500 |  |
| Beltrami et al. [55] | 4 * 4 min *CPO* |  |  | 450 ± 246 |  |  |
|  | 4 * 4 min *DPO* |  |  | 576 ± 210 |  |  |
| Fennell & Hopker [56] | 6 * 4 min (PAS recovery) | 1168 ± 141 |  | 806 ± 266 | 516 ± 263 |  |
|  | 6 * 4 min (80% W_Lactate threshold_) | 1034 ± 358 |  | 669 ± 392 | 444 ± 328 |  |
|  | 6 * 4 min (110% W_Lactate threshold_) | 1161 ± 372 |  | 749 ± 417 | 523 ± 384 |  |
|  | 3 * 8 min (PAS recovery) | 1217 ± 131 |  | 841 ± 321 | 499 ± 301 |  |
|  | 3 * 8 min (80% W_Lactate threshold_) | 1116 ± 334 |  | 686 ± 320 | 383 ± 274 |  |
|  | 3 * 8 min (110% W_Lactate threshold_) | 1101 ± 323 |  | 640 ± 373 | 377 ± 332 |  |
| Fennell & Hopker [29] | 6 * 4 min (2 min recovery) | 1178 ± 139 |  | 821 ± 311 | 502 ± 332 |  |
|  | 6 * 4 min (*IND* recovery) | 1156 ± 153 |  | 749 ± 364 | 451 ± 390 |  |
|  | 3 * 8 min (4 min recovery) | 1202 ± 126 |  | 753 ± 396 | 398 ± 330 |  |
|  | 3 * 8 min (*IND* recovery) | 1176 ± 176 |  | 649 ± 345 | 278 ± 258 |  |
| Ksoll et al. [57] | 30 * 30 s | 122 ± 371 |  |  |  |  |
|  | 5 * 3 min | 377 ± 254 |  |  |  |  |
| McKee et al. [58] ^B^ | 3 * 3 min (*Start of the Session)* |  |  | 241 ± 35 |  |  |
|  | 3 * 3 min (*Middle of the Session)* |  |  | 250 ± 34 |  |  |
|  | 3 * 3 min (*End of the Session)* |  |  | 247 ± 30 |  |  |
| Sousa et al. [59] | 2 * (6 * 10 s) |  |  | 14.7 ± 7.1% |  |  |
|  | 6 * 1 min |  |  | 11.3 ± 4.9% |  |  |
| Balci et al. [60] ^B^ | 16 * 45 s |  |  | 394 ± 83 | 93 ± 44 |  |
|  | 4 * 3 min |  |  | 553 ± 36 | 329 ± 49 |  |
|  | 6 * 30 s |  |  | 53 ± 10 | 3 ± 1 |  |
| Bogdanis et al. [61] ^B^ | 48 * 10 s | 88 ± 98 | 15 ± 31 | 0 ± 0 | 0 ± 0 |  |
|  | 16 * 30 s | 76 ± 108 | 13 ± 33 | 0 ± 0 | 0 ± 0 |  |
|  | 8 * 1 min | 238 ± 118 | 151 ± 100 | 73 ± 77 | 35 ± 46 |  |
| Duc et al. [62] | 6 * 5 min *VPO* |  |  | 528 ± 414 |  |  |
|  | 6 * 5 min *VPO* (+ vibration) |  |  | 666 ± 456 |  |  |
| Järvinen et al. [63] | 2 * (10 * 30 s) |  |  | 785 ± 192 |  |  |
| Wommer et al. [64] | 8 * 1 min *CPO* |  |  | 274 ± 132 |  |  |
|  | 8 * 1 min *DPO* |  |  | 313 ± 102 |  |  |
|  | 8 * 1 min *IPO* |  |  | 310 ± 113 |  |  |
| Bossi et al. [65] | X * 4 min at 70% Δ |  |  | 533 ± 354 | 278 ± 333 |  |
|  | X * 4 min at 85% MAP |  |  | 486 ± 336 | 260 ± 244 |  |
|  | X * 4 min at 120% W_20km TT_ |  |  | 317 ± 296 | 140 ± 112 |  |
|  | X * 4 min at 80% W' |  |  | 392 ± 393 | 209 ± 171 |  |
| Bossi et al. [66] ^B^ | 6 * 5 min *VPO* (+ vibration) |  |  | 399 ± 209 |  |  |
| Miller et al. [67] | 3 * 4 min *CPO* |  |  | 26.1 ± 11.8% |  |  |
|  | 3 * 4 min *DPO* |  |  | 25.2 ± 8.8% |  |  |
| Norouzi et al. [68] | 16 – 18 * 1 min |  |  |  | 26 ± 37 |  |
|  | 4 – 7 * 50% T_max_ |  |  |  | 208 ± 111 |  |
|  | 4 – 6 * 3 min |  |  |  | 272 ± 127 |  |
| Twist et al. [69] | 3 * (12 * 15 s) |  |  | 128 ± 133 |  |  |
| Bossi et al. [70] | V1 - X * 4 min |  |  | 447 ± 297 | 268 ± 243 |  |
|  | V2 – X * 4 min |  |  | 511 ± 322 | 314 ± 285 |  |
|  | V3 – X * 4 min |  |  | 496 ± 436 | 305 ± 361 |  |
|  | V4 – X * 4 min |  |  | 552 ± 392 | 394 ± 334 |  |
| Odden et al. [38] | 5 * 8 min *CPO* |  |  | 623 ± 595 |  |  |
|  | 5 * 8 min *VPO* (30 s intervals) |  |  | 530 ± 603 |  |  |
|  | 5 * 8 min *VPO* (1 min intervals) |  |  | 543 ± 608 |  |  |
| Urianstad et al. [71] | **♀ -** 6 * 8 min *CPO* |  |  | 863 ± 833 |  |  |
|  | **♀ -** 6 * 8 min *VPO* (30 s intervals) |  |  | 995 ± 814 |  |  |
|  | **♀ -** 6 * 8 min *VPO* (1 min intervals) |  |  | 925 ± 806 |  |  |
|  | **♂ -** 6 * 8 min *CPO* |  |  | 902 ± 699 |  |  |
|  | **♂ -** 6 * 8 min *VPO* (30 s intervals) |  |  | 1300 ± 713 |  |  |
|  | **♂ -** 6 * 8 min *VPO* (1 min intervals) |  |  | 906 ± 651 |  |  |
| Appelhans et al. [72] | 6 * 5 min, fixed |  |  | 178 ± 159 |  |  |
|  | 6 * 5 min, *A-CPO Isoeffort Max* |  |  | 1016 ± 312 |  |  |
|  | 3 * 9 min 45 s *VPO fixed* |  |  | 420 ± 259 |  |  |
|  | 3 * 9 min 45 s, *A-CPO VPO* |  |  | 1130 ± 204 |  |  |
| Boynton et al. [39] | 5 * 4 min in 13° |  |  | 875 ± 295 |  |  |
|  | 5 * 4 min in 35° |  |  | 420 ± 365 |  |  |
| Çabuk et al. [74] | 4 * (3 * 10 s) | 62.9 ± 46.1 |  |  |  |  |
|  | 4 * (2 * 15 s) | 76.6 ± 36.8 |  |  |  |  |
|  | 4 * 30 s | 27.9 ± 28.4 |  |  |  |  |
| Raimundo et al. [75] | V1 – 10 * 1 min |  |  | 91 ± 92 | 36 ± 61 |  |
|  | V2 – 10 * 1 min |  |  | 126 ± 137 | 67 ± 99 |  |
|  | V1 – 5 * 2 min |  |  | 205 ± 116 | 116 ± 115 |  |
|  | V2 – 5 * 2 min |  |  | 205 ± 126 | 123 ± 121 |  |
| Twist et al. [76] | 2 * 15 / 15 s Series |  |  | 176 ± 135 |  |  |
|  | 2 * 30 / 30 s Series |  |  | 102 ± 106 |  |  |
|  |  |  |  |  |  |  |
| **Jumping** |  |  |  |  |  |  |
| Ducrocq et al. [77] | 154 Jumps – (15 s) |  |  | 23 ± 26 |  |  |
|  | 198 Jumps (15 s) |  |  | 141 ± 151 |  |  |
|  |  |  |  |  |  |  |
| **Kayaking** |  |  |  |  |  |  |
| Paquette et al. [78] | 2 * (20 * 15 s) |  |  | 486 ± 372 | 294 ± 318 |  |
|  | 2 * (10 * 30 s) |  |  | 408 ± 276 | 210 ± 258 |  |
|  | 6 * 30 s |  |  | 102 ± 78 | 60 ± 60 |  |
|  | 6 * 1 min |  |  | 246 ± 102 | 174 ± 108 |  |
|  |  |  |  |  |  |  |
| **Rowing** |  |  |  |  |  |  |
| *Patterson et al. [79]* | X * 30 s |  |  |  | 12 ± 36 |  |
|  | X * 1 min (30 s recovery) |  |  |  | 0 ± 0 |  |
|  | X * 1 min (1 min recovery) |  |  |  | 18 ± 48 |  |
|  | X * 2 min (1 min recovery) |  |  |  | 78 ± 138 |  |
|  | X * 2 min (2 min recovery) |  |  |  | 90 ± 288 |  |
|  | X * 3 min (1.5 min recovery) |  |  |  | 186 ± 168 |  |
|  | X * 3 min (3 min recovery) |  |  |  | 324 ± 222 |  |
| Faelli et al. [80] ^C^ | 25 * 30 s |  |  | 493 ± 109 |  |  |
|  | 4 * 4 min |  |  | 790 ± 60 |  |  |
|  |  |  |  |  |  |  |
| **Running** |  |  |  |  |  |  |
| Billat et al. [34] | X * 30 s |  |  |  | 471 ± 398 |  |
| Demarie et al. [33] ^A^ | X * 50% T_max_ |  |  |  |  | 623 ± 310 |
| Billat et al. [81] | X * 15 s at 90% vVO_2_max |  |  |  |  | 861 ± 240 |
|  | X * 15 s at vVO_2_max |  |  |  |  | 871 ± 330 |
|  | X * 15 s at 110% vVO_2_max |  |  |  |  | 444 ± 120 |
| Dupont et al. [82] | X * 15 s at 110% MAV |  |  | 383 ± 180 | 120 ± 42 |  |
|  | X * 15 s at 120% MAV |  |  | 323 ± 272 | 202 ± 66 |  |
|  | X * 15 s at 130% MAV |  |  | 135 ± 133 | 50 ± 47 |  |
|  | X * 15 s at 140% MAV |  |  | 77 ± 96 | 48 ± 59 |  |
| Millet et al. [83] | 3 * (8 * 30 s) |  |  | 149 ± 133 | 54 ± 50 |  |
|  | 3 * (4 * 1 min) |  |  | 531 ± 187 | 196 ± 142 |  |
|  | 3 * (2 * 50% Tmax) |  |  | 486 ± 176 | 282 ± 199 |  |
| Millet et al. [84] | 3 * (8 * 30 s at vVO_2_max) |  |  | 168 ± 131 |  |  |
|  | 3 * (8 * 30 s at 105% vVO_2_max) |  |  | 338 ± 149 |  |  |
| Dupont & Berthoin [85] | X * 15 s (*ACT* recovery) |  |  | 282 ± 117 |  |  |
|  | X * 15 s (*PAS* recovery) |  |  | 317 ± 132 |  |  |
| Tardieu-Berger et al. [86] ^C^ | X * 30 s |  |  | 345 ± 204 | 178 ± 187 |  |
|  | X * (6 * 30 s) |  |  | 290 ± 280 | 158 ± 198 |  |
| Midgley et al. [87] | V1 – X * 30 s |  |  | 480 ± 220 | 232 ± 174 |  |
|  | V2 – X * 30 s |  |  | 488 ± 252 | 244 ± 195 |  |
| Midgley et al. [88] | X * 30 s |  |  |  | 418 ± 439 | 163 ± 227 |
| Rozenek et al. [89] | X * 15 s |  |  | 0 ± 0 |  |  |
|  | X * 30 s |  |  | 248 ± 173 |  |  |
|  | X * 1 min |  |  | 324 ± 118 |  |  |
| Thevenet et al. [37], and Thevenet et al. [90] | X * 30 s (50% MAV) |  |  | 746 ± 417 | 459 ± 332 |  |
|  | X * 30 s (67% MAV) |  |  | 587 ± 259 |  |  |
|  | X * 30 s (84% MAV) |  |  | 306 ± 85 |  |  |
|  | X * 15 s (*PAS* recovery) |  |  | 548 ± 499 | 316 ± 360 |  |
| Thevenet et al. [91] | X * 30 s at MAV |  |  | 300 ± 150 | 118 ± 66 |  |
|  | X * 30 s at 110% MAV |  |  | 198 ± 148 | 62 ± 75 |  |
| O’Brien et al. [92] | 10 * 1 min |  |  | 79 ± 236 |  |  |
|  | 5 * 2 min |  |  | 268 ± 213 |  |  |
| Wakefield & Glaister [93] ^B^ | X * 20 s at 105% vVO_2_max |  |  |  | 43 ± 40 |  |
|  | X * 25 s at 105% vVO_2_max |  |  |  | 60 ± 51 |  |
|  | X * 30 s at 105% vVO_2_max |  |  |  | 156 ± 93 |  |
|  | X * 20 s at 115% vVO_2_max |  |  |  | 26 ± 36 |  |
|  | X * 25 s at 115% vVO_2_max |  |  |  | 38 ± 36 |  |
|  | X * 30 s at 115% vVO_2_max |  |  |  | 86 ± 58 |  |
| Zafeiridis et al. [94] | X * 30 s | 467 ± 174 | 277 ± 210 | 149 ± 147 | 54 ± 66 |  |
|  | X * 3 min | 738 ± 201 | 562 ± 242 | 392 ± 276 | 220 ± 240 |  |
| Zafeiridis et al. [95] ^B^ | YA – X * 30 s | 608 ± 222 |  | 309 ± 204 | 81 ± 82 |  |
|  | YA – X * 3 min | 838 ± 266 |  | 625 ± 234 | 374 ± 199 |  |
|  | Men – X * 30 s | 852 ± 267 |  | 333 ± 199 | 95 ± 115 |  |
|  | Men – X * 3 min | 784 ± 257 |  | 502 ± 280 | 286 ± 261 |  |
| Buchheit et al. [96] | 5 * 3 min |  |  | 544 ± 164 |  |  |
| Abderrahman et al. [97] | G1 PRE – X * 30 s |  |  | 338 ± 317 | 161 ± 158 |  |
|  | G1 POST – X * 30 s |  |  | 477 ± 442 | 231 ± 243 |  |
|  | G2 PRE – X * 30 s |  |  | 543 ± 293 | 335 ± 313 |  |
|  | G2 POST – X * 30 s |  |  | 635 ± 428 | 497 ± 365 |  |
| De Aguiar et al. [98] | X * 30 s at 105% ICV |  |  |  | 106 ± 71 |  |
|  | X * 30 s at 125% ICV |  |  |  | 113 ± 40 |  |
|  | X * 30 s at 125 - 105% ICV, *DV* |  |  |  | 286 ± 150 |  |
| Panissa et al. [99] | Moderate VO_2_max – X * 1 min |  |  | 30.2 ± 9.1% |  |  |
|  | High VO_2_max – X * 1 min |  |  | 7.3 ± 6% |  |  |
| Panasci et al. [100] | 15 * 30 s at MAV (track) |  |  | 38.7 ± 32.5% |  |  |
|  | 15 * 30 s at MAV (treadmill) |  |  | 8.6 ± 11.5% |  |  |
|  | 15 * 30 s at 115% MAV (treadmill) |  |  | 39.9 ± 23% |  |  |
| Smilios et al. [101] | 4 * 4 min (2 min recovery) | 775 ± 44 |  | 546 ± 135 | 265 ± 229 |  |
|  | 4 * 4 min (3 min recovery) | 751 ± 58 |  | 521 ± 174 | 276 ± 209 |  |
|  | 4 * 4 min (4 min recovery) | 749 ± 59 |  | 463 ± 198 | 234 ± 231 |  |
| Baquet et al. [102] | X * 15 s (*ACT* recovery) | 113 ± 111 |  | 72 ± 116 |  |  |
|  | X * 15 s (*PAS* recovery) | 151 ± 173 |  | 41 ± 56 |  |  |
| Schoenmakers & Reed [103] | 6 * 4 min (1 min recovery) |  |  | 849 ± 341 | 574 ± 373 |  |
|  | 6 * 4 min (2 min recovery) |  |  | 727 ± 388 | 422 ± 347 |  |
|  | 6 * 4 min (3 min recovery) |  |  | 918 ± 232 | 629 ± 330 |  |
|  | 6 * 4 min (self-selected recovery) |  |  | 776 ± 335 | 476 ± 408 |  |
| Ducrocq et al. [77] | 22 * 15 s |  |  | 145 ± 76 |  |  |
| Myrkos et al. [104] | X * 1 min (30 s recovery) | 803 ± 365 |  | 392 ± 319 | 211 ± 282 |  |
|  | X * 1 min (1 min recovery) | 850 ± 295 |  | 324 ± 228 | 101 ± 91 |  |
|  | X * 2 min (1 min recovery) | 603 ± 88 |  | 405 ± 142 | 226 ± 160 |  |
|  | X * 2 min (2 min recovery) | 764 ± 212 |  | 461 ± 316 | 301 ± 272 |  |
| Beltrami et al. [55] | 4 * 4 min, *CV* |  |  | 624 ± 186 |  |  |
|  | 4 * 4 min, *DV* |  |  | 650 ± 240 |  |  |
| José Arantes et al. [105] ^B, C^ | X * 30 s |  |  | 274 ± 48 |  |  |
|  | X * 40 s (20 s recovery) |  |  | 209 ± 25 |  |  |
|  | X * 40 s (40 s recovery) |  |  | 284 ± 73 |  |  |
| Germano et al. [106] ^B^ | SAR - (short *ACT* recovery) | 1323 ± NR | 878 ± NR | 526 ± NR | 232 ± 102 |  |
|  | SPR - (short *PAS* recovery) | 1435 ± NR | 918 ± NR | 566 ± NR | 236 ± 153 |  |
|  | LAR (long *ACT* recovery) | 1471 ± NR | 1043 ± NR | 693 ± NR | 310 ± 176 |  |
|  | LPR (long *PAS* recovery) | 2073 ± NR | 1506 ± NR | 919 ± NR | 410 ± 176 |  |
| Sánchez-Otero et al. [107] | 4 * 2 min (*ACT* recovery) |  |  | 222 ± 73 |  |  |
|  | 4 * 2 min (*PAS* recovery) |  |  | 230 ± 104 |  |  |
| Bok et al. [108] ^B^ | 20 * 15 s at 110% vVO_2_max |  |  | 158 ± 74 |  |  |
|  | 20 * 15 s at Δ15% ASR |  |  | 180 ± 82 |  |  |
|  | 20 * 15 s at Δ25% ASR + |  |  | 172 ± 79 |  |  |
| Held et al. [109] ^A^ | 4 * 5 min |  |  | 384 ± 240 |  |  |
|  | 4 * 5 min (uphill) |  |  | 546 ± 276 |  |  |
| Twist et al. [69] | 3 * (12 * 15 s) |  |  | 288 ± 132 |  |  |
| Varela-Sanz et al. [110] | X * 2 min (*ACT* recovery) |  |  | 705 ± 395 |  |  |
|  | X * 2 min (*PAS* recovery) |  |  | 666 ± 415 |  |  |
| Vaccari et al. [111] | X * 30 s |  |  | 167 ± 188 |  |  |
|  | X * 3 min |  |  | 349 ± 111 |  |  |
|  | X * *HIDIT* |  |  | 579 ± 219 |  |  |
| Fleckenstein et al. [112] ^A^ | 24 * 30 s |  |  | 201 ± 268 | 58 ± 139 |  |
|  | 4 * 3 min |  |  | 328 ± 147 | 148 ± 127 |  |
| **Swimming** |  |  |  |  |  |  |
| Bentley et al. [113] | 16 * 100 m |  |  | 564 ± 494 |  |  |
|  | 4 * 400 m |  |  | 341 ± 328 |  |  |
| Libicz et al. [114] ^B^ | 16 * 50 m |  |  |  | 69 ± 63 |  |
|  | 8 * 100 m |  |  |  | 145 ± 166 |  |
| Almeida et al. [115] | 8 * 100 m |  |  | 208 ± 124 | 97 ± 100 |  |
|  | 4 * 200 m |  |  | 218 ± 122 | 86 ± 109 |  |
| Almeida et al. [116] | X * 100 m |  |  | 322 ± 209 |  |  |
|  |  |  |  |  |  |  |
| **Cross Country Skiing** |  |  |  |  |  |  |
| Rønnestad et al. [117] | 5 * 5 min, *CV* |  |  | 650 ± 343 |  |  |
|  | 5 * 5 min, *DV* |  |  | 717 ± 245 |  |  |
| Rønnestad et al. [118] | 5 * 5 min, *CV* |  |  | 792 ± 246 |  |  |
|  | 5 * 5 min, *DV* |  |  | 876 ± 252 |  |  |
|  | 5 * 5 min, *VV* |  |  | 900 ± 240 |  |  |
| Rønnestad et al. ­­[119] | V1 – 6 * 5 min, *VV* |  |  | 713 ± 477 |  |  |
|  | V2 – 6 * 5 min, *VV* |  |  | 838 ± 414 |  |  |
|  | V3 – 6 * 5 min, *VV* |  |  | 948 ± 397 |  |  |
