# Supplementary material for: Time spent at or near V̇O₂max during high-intensity interval training - a systematic review and meta-analysis
Source: BMC Sports Sci Med Rehabil. 2026 Jun 3;18:358. doi: 10.1186/s13102-026-01766-x (PMC13480085; doi:10.1186/s13102-026-01766-x)
Supplement: Supplementary file 3 [file 13102_2026_1766_MOESM3_ESM.docx]

**Supplementary table 3:** Study quality and risk of bias assessment

|  | **PEDro Scale** | | | | | | | | |  | **RoB-2 for Crossover Trials** | | | | | | |
| --- | --- | --- | --- | --- | --- | --- | --- | --- | --- | --- | --- | --- | --- | --- | --- | --- | --- |
|  | **1** | **2** | **3** | **4** | **5** | **6** | **7** | **8** | **Total** |  | **D1** | **DS** | **D2** | **D3** | **D4** | **D5** | **Overall** |
| **Cycling:** |  |  |  |  |  |  |  |  |  |  |  |  |  |  |  |  |  |
| Zuniga et al. [41] | Y | 1 | 0 | 1 | 1 | 1 | 1 | 1 | 6 |  | LR | SR | LR | LR | LR | SR | SR |
| Nicolò et al. [42] | Y | 1 | 0 | 1 | 1 | 1 | 1 | 1 | 6 |  | LR | LR | LR | LR | LR | LR | LR |
| Lisbôa et al. [43] | Y | 1 | 0 | 1 | 1 | 1 | 1 | 1 | 6 |  | LR | LR | LR | LR | LR | LR | LR |
| Zadow et al. [44] | Y | 1 | 0 | 1 | 1 | 1 | 1 | 1 | 6 |  | LR | LR | LR | LR | LR | SR | LR |
| Zafeiridis et al. [45] | Y | 1 | 0 | 1 | 1 | 1 | 1 | 1 | 6 |  | LR | LR | LR | LR | LR | SR | LR |
| Rønnestad & Hansen [46] | Y | 1 | 0 | 1 | 1 | 1 | 1 | 1 | 6 |  | LR | LR | LR | LR | LR | LR | LR |
| Turnes et al. [39] | Y | 0 | 0 | 1 | 1 | 1 | 0 | 1 | 4 |  | NA | NA | LR | LR | LR | LR | LR |
| Combes et al. [47] | Y | 0 | 0 | 1 | 1 | 1 | 0 | 1 | 4 |  | NA | NA | LR | LR | LR | LR | LR |
| Rønnestad et al. [48] | Y | 1 | 0 | 1 | 1 | 1 | 1 | 1 | 6 |  | LR | LR | LR | SR | LR | LR | LR |
| Shi et al. [49] | Y | 1 | 0 | 1 | 1 | 1 | 1 | 1 | 6 |  | LR | SR | LR | LR | LR | LR | LR |
| Boynton et al. [50] | Y | 1 | 0 | 1 | 1 | 1 | 1 | 1 | 6 |  | LR | SR | LR | LR | LR | LR | LR |
| Lisbôa et al. [51] | Y | 1 | 0 | 1 | 1 | 1 | 1 | 1 | 6 |  | LR | LR | LR | LR | LR | SR | LR |
| Almquist et al. [52] | Y | 1 | 0 | 1 | 1 | 1 | 1 | 1 | 6 |  | LR | SR | LR | LR | LR | LR | LR |
| Bossi et al. [31] | Y | 1 | 0 | 1 | 1 | 1 | 1 | 1 | 6 |  | LR | LR | LR | LR | LR | LR | LR |
| Vaccari et al. [53] | Y | 1 | 0 | 1 | 1 | 1 | 1 | 1 | 6 |  | LR | LR | LR | LR | LR | LR | LR |
| Dall’Agnol et al. [54] | Y | 1 | 0 | 1 | 1 | 1 | 1 | 1 | 6 |  | LR | SR | LR | LR | LR | LR | LR |
| Beltrami et al. [55] | Y | 1 | 0 | 1 | 1 | 1 | 1 | 1 | 6 |  | LR | LR | LR | LR | LR | LR | LR |
| Fennell & Hopker [56] | Y | 1 | 0 | 1 | 1 | 1 | 1 | 1 | 6 |  | LR | SR | LR | LR | LR | LR | LR |
| Fennell & Hopker [29] | Y | 1 | 0 | 1 | 1 | 1 | 1 | 1 | 6 |  | LR | SR | LR | LR | LR | LR | LR |
| Ksoll et al. [57] | Y | 1 | 0 | 1 | 1 | 1 | 1 | 1 | 6 |  | LR | LR | LR | LR | LR | SR | LR |
| McKee et al. [58] | Y | 1 | 0 | 1 | 1 | 1 | 1 | 1 | 6 |  | LR | SR | LR | LR | LR | LR | LR |
| Sousa et al. [59] | Y | 1 | 0 | 1 | 1 | 1 | 0 | 1 | 5 |  | LR | LR | LR | LR | LR | HR | HR |
| Balci et al. [60] | Y | 1 | 0 | 1 | 1 | 1 | 1 | 1 | 6 |  | LR | SR | LR | LR | LR | LR | LR |
| Bogdanis et al. [61] | Y | 1 | 0 | 1 | 1 | 1 | 1 | 1 | 6 |  | LR | SR | LR | LR | LR | LR | LR |
| Duc et al. [62] | Y | 1 | 0 | 1 | 1 | 1 | 1 | 1 | 6 |  | LR | LR | LR | LR | LR | LR | LR |
| Järvinen et al. [63] | Y | 0 | 0 | 1 | 1 | 1 | 0 | 1 | 4 |  | NA | NA | LR | SR | LR | LR | LR |
| Wommer et al. [64] | Y | 1 | 0 | 1 | 1 | 1 | 1 | 1 | 6 |  | LR | LR | LR | LR | LR | LR | LR |
| Bossi et al. [65] | Y | 1 | 0 | 1 | 1 | 1 | 1 | 1 | 6 |  | LR | SR | LR | LR | LR | LR | LR |
| Bossi et al. [66] | Y | 1 | 0 | 1 | 1 | 1 | 1 | 1 | 6 |  | LR | LR | LR | LR | LR | LR | LR |
| Miller et al. [67] | Y | 1 | 0 | 1 | 1 | 1 | 1 | 1 | 6 |  | LR | LR | LR | LR | LR | HR | HR |
| Norouzi et al. [68] | Y | 1 | 0 | 1 | 1 | 1 | 1 | 1 | 6 |  | LR | LR | LR | LR | LR | SR | LR |
| Twist et al. [69] | Y | 0 | 0 | 1 | 1 | 1 | 0 | 1 | 4 |  | NA | NA | LR | LR | LR | LR | LR |
| Bossi et al. [70] | Y | 1 | 0 | 1 | 1 | 1 | 1 | 1 | 6 |  | LR | SR | LR | LR | LR | LR | LR |
| Odden et al. [38] | Y | 1 | 0 | 1 | 0 | 1 | 1 | 1 | 5 |  | LR | SR | LR | SR | LR | LR | LR |
| Urianstad et al. [71] | Y | 1 | 0 | 1 | 1 | 1 | 1 | 1 | 6 |  | LR | HR | LR | LR | LR | LR | SR |
| Appelhans et al. [72] | Y | 1 | 0 | 1 | 1 | 1 | 1 | 1 | 6 |  | LR | SR | LR | LR | LR | LR | LR |
| Boynton et al. [39] | Y | 0 | 0 | 1 | 1 | 1 | 0 | 1 | 4 |  | NA | NA | LR | LR | SR | LR | LR |
| Çabuk et al. [74] | Y | 1 | 0 | 1 | 1 | 1 | 1 | 1 | 6 |  | LR | LR | LR | LR | LR | SR | LR |
| Raimundo et al. [75] | Y | 1 | 0 | 0 | 1 | 1 | 1 | 1 | 6 |  | LR | LR | LR | LR | LR | LR | LR |
| Twist et al. [76] | Y | 1 | 0 | 1 | 1 | 1 | 1 | 1 | 6 |  | LR | SR | LR | LR | LR | LR | LR |
| **Jumping (and Running)** |  |  |  |  |  |  |  |  |  |  |  |  |  |  |  |  |  |
| Ducrocq et al. [77] | Y | 1 | 0 | 1 | 1 | 1 | 1 | 1 | 6 |  | LR | SR | LR | LR | LR | LR | LR |
| **Kayaking** |  |  |  |  |  |  |  |  |  |  |  |  |  |  |  |  |  |
| Paquette et al. [78] | Y | 1 | 0 | 1 | 1 | 1 | 1 | 1 | 6 |  | LR | SR | LR | LR | LR | LR | LR |
| **Rowing** |  |  |  |  |  |  |  |  |  |  |  |  |  |  |  |  |  |
| Patterson et al. [79] | Y | 1 | 0 | 1 | 1 | 1 | 1 | 1 | 6 |  | LR | HR | LR | LR | LR | SR | HR |
| Faelli et al. [80] | Y | 1 | 0 | 1 | 1 | 1 | 1 | 1 | 6 |  | LR | SR | LR | LR | LR | LR | LR |
| **Running** |  |  |  |  |  |  |  |  |  |  |  |  |  |  |  |  |  |
| Billat et al. [34] | Y | 0 | 0 | 1 | 1 | 1 | 0 | 1 | 4 |  | NA | NA | LR | LR | LR | SR | LR |
| Demarie et al. [33] | Y | 0 | 0 | 1 | 1 | 1 | 0 | 1 | 4 |  | NA | NA | LR | LR | LR | SR | LR |
| Billat et al. [81] | Y | 1 | 0 | 1 | 1 | 1 | 1 | 1 | 6 |  | LR | LR | LR | LR | LR | SR | LR |
| Dupont et al. [82] | Y | 1 | 0 | 1 | 1 | 1 | 1 | 1 | 6 |  | LR | SR | LR | LR | LR | LR | LR |
| Millet et al. [83] | Y | 1 | 0 | 1 | 1 | 1 | 1 | 1 | 6 |  | LR | SR | LR | LR | LR | LR | LR |
| Millet et al. [84] | Y | 1 | 0 | 1 | 1 | 1 | 1 | 1 | 6 |  | LR | LR | LR | LR | LR | LR | LR |
| Dupont & Berthoin [85] | Y | 1 | 0 | 1 | 1 | 1 | 1 | 1 | 6 |  | LR | LR | LR | LR | LR | LR | LR |
| Tardieu-Berger et al. [86] | Y | 1 | 0 | 1 | 1 | 1 | 1 | 1 | 6 |  | LR | LR | LR | LR | LR | LR | LR |
| Midgley et al. [87] | Y | 0 | 0 | 1 | 1 | 1 | 1 | 1 | 5 |  | LR | LR | LR | LR | LR | LR | LR |
| Midgley et al. [88] | Y | 0 | 0 | 1 | 1 | 1 | 0 | 1 | 4 |  | NA | NA | LR | LR | LR | SR | LR |
| Rozenek et al. [89] | Y | 1 | 0 | 1 | 0 | 0 | 1 | 1 | 4 |  | LR | LR | LR | SR | LR | LR | LR |
| Thevenet et al. [37] | Y | 1 | 0 | 1 | 1 | 1 | 1 | 1 | 6 |  | LR | LR | LR | LR | LR | LR | LR |
| Thevenet et al. [90] | Y | 1 | 0 | 1 | 1 | 1 | 1 | 1 | 6 |  | LR | SR | LR | LR | LR | LR | LR |
| Thevenet et al. [91] | Y | 1 | 0 | 1 | 1 | 1 | 1 | 1 | 6 |  | LR | LR | LR | LR | LR | LR | LR |
| O’Brien et al. [92] | Y | 1 | 0 | 1 | 1 | 1 | 1 | 1 | 6 |  | LR | LR | LR | LR | LR | LR | LR |
| Wakefield & Glaister [93] | Y | 1 | 0 | 1 | 1 | 1 | 1 | 1 | 6 |  | LR | SR | LR | LR | LR | SR | SR |
| Zafeiridis et al. [94] | Y | 1 | 0 | 1 | 1 | 1 | 1 | 1 | 6 |  | LR | LR | LR | LR | LR | SR | LR |
| Zafeiridis et al. [95] | Y | 1 | 0 | 1 | 1 | 1 | 1 | 1 | 6 |  | LR | LR | LR | LR | LR | SR | LR |
| Buchheit et al. [96] | Y | 0 | 0 | 1 | 1 | 1 | 0 | 1 | 4 |  | NA | NA | LR | LR | LR | LR | LR |
| Abderrahman et al. [97] | Y | 1 | 0 | 1 | 0 | 1 | 1 | 1 | 4 |  | NA | NA | LR | SR | LR | LR | LR |
| De Aguiar et al. [98] | Y | 1 | 0 | 1 | 1 | 1 | 1 | 1 | 6 |  | LR | LR | LR | LR | LR | SR | LR |
| Panissa et al. [99] | Y | 0 | 0 | 0 | 1 | 1 | 1 | 1 | 4 |  | SR | NA | LR | LR | LR | HR | HR |
| Panasci et al. [100] | Y | 1 | 0 | 1 | 1 | 1 | 1 | 1 | 6 |  | LR | SR | LR | LR | LR | HR | HR |
| Smilios et al. [101] | Y | 1 | 0 | 1 | 1 | 1 | 1 | 1 | 6 |  | LR | SR | LR | LR | LR | LR | LR |
| Baquet et al. [102] | Y | 1 | 0 | 1 | 1 | 1 | 1 | 1 | 6 |  | LR | LR | LR | LR | LR | LR | LR |
| Schoenmakers & Reed [103] | Y | 1 | 0 | 1 | 1 | 1 | 1 | 1 | 6 |  | LR | SR | LR | LR | LR | LR | LR |
| Myrkos et al. [104] | Y | 1 | 0 | 1 | 1 | 1 | 1 | 1 | 6 |  | LR | SR | LR | LR | LR | SR | SR |
| Beltrami et al. [55] | Y | 1 | 0 | 1 | 1 | 1 | 1 | 1 | 6 |  | LR | LR | LR | LR | LR | LR | LR |
| José Arantes et al. [105] | Y | 1 | 0 | 1 | 1 | 1 | 1 | 1 | 6 |  | LR | SR | LR | LR | LR | LR | LR |
| Germano et al. [106] | Y | 1 | 0 | 1 | 1 | 1 | 1 | 1 | 6 |  | LR | SR | LR | LR | LR | SR | SR |
| Sánchez-Otero et al. [107] | Y | 1 | 0 | 1 | 1 | 1 | 1 | 1 | 6 |  | LR | LR | LR | LR | LR | LR | LR |
| Bok et al. [108] | Y | 1 | 0 | 1 | 0 | 1 | 1 | 1 | 5 |  | LR | LR | LR | SR | LR | LR | LR |
| Held et al. [109] | Y | 1 | 0 | 1 | 1 | 1 | 1 | 1 | 6 |  | LR | LR | LR | LR | LR | LR | LR |
| Twist et al. [69] | Y | 0 | 0 | 1 | 1 | 1 | 0 | 1 | 4 |  | NA | NA | LR | LR | LR | LR | LR |
| Varela-Sanz et al. [110] | Y | 1 | 0 | 1 | 1 | 1 | 1 | 1 | 6 |  | LR | LR | LR | LR | LR | LR | LR |
| Vaccari et al. [111] | Y | 1 | 0 | 1 | 0 | 1 | 1 | 1 | 5 |  | LR | LR | LR | SR | LR | LR | LR |
| Fleckenstein et al. [112] | Y | 1 | 0 | 1 | 1 | 1 | 1 | 1 | 6 |  | LR | LR | LR | LR | LR | LR | LR |
| **Swimming** |  |  |  |  |  |  |  |  |  |  |  |  |  |  |  |  |  |
| Bentley et al. [113] | Y | 1 | 0 | 1 | 1 | 1 | 1 | 1 | 6 |  | LR | LR | LR | LR | LR | LR | LR |
| Libicz et al. [114] | Y | 1 | 0 | 1 | 1 | 1 | 1 | 1 | 6 |  | LR | SR | LR | LR | LR | SR | SR |
| Almeida et al. [115] | Y | 1 | 0 | 1 | 1 | 1 | 1 | 1 | 6 |  | LR | LR | LR | LR | LR | LR | LR |
| Almeida et al. [116] | Y | 0 | 0 | 1 | 1 | 1 | 0 | 1 | 4 |  | NA | NA | LR | LR | LR | LR | LR |
| **Cross Country Skiing** |  |  |  |  |  |  |  |  |  |  |  |  |  |  |  |  |  |
| Rønnestad et al. [117] | Y | 1 | 0 | 1 | 1 | 1 | 1 | 1 | 6 |  | LR | LR | LR | LR | LR | LR | LR |
| Rønnestad et al. [118] | Y | 1 | 0 | 1 | 1 | 1 | 1 | 1 | 6 |  | LR | SR | LR | LR | LR | LR | LR |
| Rønnestad et al. ­­[119] | Y | 0 | 0 | 1 | 1 | 1 | 1 | 1 | 5 |  | LR | SR | LR | SR | LR | LR | SR |

**Items in the PEDro scale**: 1 = eligibility criteria were specified; 2 = subjects were randomly allocated to groups; 3 = allocation was concealed; 4 = the groups were similar at baseline regarding the most important prognostic indicators; 5 = measures of 1 key outcome were obtained from 95% of subjects initially allocated to groups; 6 = all subjects for whom outcome measures were available received the treatment or control condition as allocated or, where this was not the case, data for at least 1 key outcome were analyzed by "intention to treat"; 7 = the results of between‑group statistical comparison are reported for at least 1 key outcome; 8 = the study provides both point measures and measures of variability for at least 1 key outcome

**Domains in the RoB-2 Scale:** D1 = bias arising from the randomization process; DS = bias due to period and carryover effects; D2 = bias due to deviations from intended interventions; D3 = bias due to missing outcome data; D4 = bias in measurement of the outcome; D5 = bias in selection of the reported result.
