# Supplementary material for: Time spent at or near V̇O₂max during high-intensity interval training - a systematic review and meta-analysis
Source: BMC Sports Sci Med Rehabil. 2026 Jun 3;18:358. doi: 10.1186/s13102-026-01766-x (PMC13480085; doi:10.1186/s13102-026-01766-x)
Supplement: Supplementary file 4 [file 13102_2026_1766_MOESM4_ESM.docx]

Supplementary table 4:

|  | **Warm-up Protocol** | **VO₂max Method** | **VO₂ Data Averaging Protocol** |
| --- | --- | --- | --- |
| **Cycling** |  |  |  |
| Zuniga et al. [41] | A 10 min warm-up consisted of cycling at a self-selected pace was performed before each HIIT protocol | VO2 of final 30 s | ***Not reported*** |
| Nicolò et al. [42] | Participants completed a warm-up that began with 3 min at 100 W, followed by a gradual increase in work rate up to 250 W over 10 min. They then performed three 15 s bouts at 400 W, each separated by 90 s at 100 W | Highest VO2 in 30 s period | Breath-by-breath data were averaged over 10 s segments |
| Lisbôa et al. [43], and Lisbôa et al. [51] | The warm-up consisted of 5 min cycling at 50% MAP followed by 5 min of passive rest | Highest VO2 in 15 s period | Breath-by-breath data were averaged over 5 segments |
| Zadow et al. [44] | Participants completed a 15 min warm-up (5 min at 30%, 40% and 50% MAP) after which they cycled for 5 additional min at a power output equal to 50% of their aerobic threshold | Highest VO2 in 30 s period | ***Not reported*** |
| Zafeiridis et al. [45] | Participants performed a standardised warm-up, consisting of 4 min of cycling with light resistance and, 4 min of stretching exercises | Highest VO2 in two successive 15 s averages | Breath-by-breath data were averaged over 5 s segments |
| Rønnestad & Hansen [46] | Participants performed a 15 min individualised warm-up that was concluded by 2–3 submaximal sprints lasting 20–30 s | Highest VO2 in two successive 30 s averages | Breath-by-breath data were averaged over 15 s segments |
| Turnes et al. [39] | Training sessions began with a standardised 5 min warm-up at an intensity of 100% LT | Highest VO2 in 15 s period | Breath-by-breath data were averaged over 15 s segments |
| Combes et al. [47] | ***Not reported* –** ‘A standardized warm-up’ | Highest VO2 in 30 s period | Breath-by-breath data were averaged over 5 s segments |
| Rønnestad et al. [48] | Participants performed a 20 min individualised warm-up with gradually increasing intensity, including 3 min at 200 W for EMG normalisation. Thereafter, 1 min recovery was given before cycling 1 min at the planned power output of the first work interval followed by 2 min recovery | Highest VO2 in two successive 30 s averages | Breath-by-breath data were averaged over 15 s segments |
| Shi et al. [49] | All trials included a standardised warm-up of 5 min cycling at 60 W, five 6 s cycle sprints at progressively increasing speeds, and 5 min stretching | Highest VO2 in 10 s period | Breath-by-breath data were averaged over 5 s segments |
| Boynton et al. [50], and Boynton et al. [39] | The warm-up consisted of a 10 min at 50% MAP at room temperature | Highest VO2 in six successive 5 s averages | Breath-by-breath data were averaged over 5 s segments |
| Almquist et al. [52] | Participants cycled 20 min at 175 W, followed by 50 W increases every 3 min until blood lactate exceeded 4 mmol, and finished with two to three submaximal sprints of 20–30 s | Highest VO2 in two successive 30 s averages | ***Not reported*** |
| Bossi et al. [31] | The warm-up consisted of 5 min at an RPE of 11 (light), followed by three 1 min intervals at 16 (hard to very hard), interspersed with two 2 min blocks and a final 3 min at RPE 9 (very light) | Highest VO2 in 60 s period | Breath-by-breath data were averaged over 5 s segments |
| Vaccari et al. [53] | A 10 min warm-up consisted of cycling at 100 W | Highest VO2 in 30 s period (or VO2 of final 30 s) | Breath-by-breath data were averaged over 5 s segments |
| Dall’Agnol et al. [54] | Participants performed a standardised 5 min warm-up, consisting of 3 min cycling at 150 W and 2 min at 100 W | Highest VO2 in 15 s period | Breath-by-breath data were filtered to remove any outlying breaths (±3 SD from the adjacent 5 breaths) and  then averaged over 5 s segments |
| Beltrami et al. [55] | Participants warmed up for 10 min at a self-selected pace. The first work interval was then preceded by an additional 5 min warm-up, including 3 min at 100 W, followed by 2 min at 50% above the difference between 100 W and the first interval intensity | Highest VO2 in six successive 5 s averages | Breath-by-breath data were averaged over 5 s segments |
| Fennell & Hopker [56], and Fennell & Hopker [29] | The warm-up consisted of 10 min cycling at 100 W | Highest VO2 in 60 s period | Raw breath-by-breath data was used to calculate outcome measures |
| Ksoll et al. [57] | Participants completed a 5 min warm-up at the intensity equal to the recovery interval (= 85% GET) | Highest VO2 in 30 s period | Breath-by-breath data were averaged over 1 s segments |
| McKee et al. [58] | ***Not reported* –** HIIT protocols were part of 90 min longer training session. | Highest VO2 in 45 s period | Breath-by-breath data were averaged over 5 s segments |
| Sousa et al. [59] | Participants performed a warm-up of 10 min at 100 W, and 4 min at specific 50% MAP | ***Not reported*** | Breath-by-breath data were smoothed using a 3-breath moving average, and then averaged over 10 s segments |
| Balci et al. [60] | A standardised warm-up included 10 min cycling at 50–65% MAP with three acceleration sprints at the 6th, 8th, and 10th minutes, followed by 4–6 min of stretching | Highest VO2 in 30 s period | ***Not reported*** |
| Bogdanis et al. [61] | Before each trial, participants cycled 3 min at 15% MAP, followed by 3 min of passive rest on the cycle ergometer | Highest VO2 in two successive 10 s averages | Breath-by-breath data were averaged over 5 s segments |
| Duc et al. [62] | Participants completed a 13 min warm-up with progressive intensities (2.5 min at 30% MAP, 1 min at 40% MAP, 1 min at 50% MAP, 1 min at 60% MAP, 1 min at 70% MAP, 30 s at 80% MAP, 30 s at 90% MAP, 30 s at MAP) and 5 min at 30% MAP | Highest VO2 in 60 s period  (Highest VO2 in six successive 10 s averages?) | Breath-by-breath data were averaged over 10 s segments |
| Järvinen et al. [63] | Each session began with a 10 min warm-up performed on a cycle ergometer at 60–70% maximum HR | Highest VO2 in six successive 5 s averages | Breath-by-breath data were averaged over 5 s segments |
| Wommer et al. [64] | Participants warmed up for 5 min warm-up at 100 %LT | Highest VO2 in 15 s period | Breath-by-breath data were filtered to remove any outlying breaths (±3 SD from the adjacent 5 breaths) and  then averaged over 5 s segments |
| Bossi et al. [65] | Participants performed two sequential 5 min warm-ups at 40% and 50% of the prescribed mean work rate for the HIIT sessions | Highest VO2 in 30 s period | Breath-by-breath data were averaged over 5 s segments |
| Bossi et al. [66] | The warm-up consisted of 5 min at RPE 11, followed by three 1 min intervals at RPE 16, interspersed with two 2 min and a final 3 min block at RPE 9 | Highest VO2 in 30 s period | Breath-by-breath data were averaged over 5 s segments |
| Miller et al. [67] | The warm-up consisted of 5 min of cycling at 50W, followed by 5 min of cycling at each participant’s GET, and then 5 min at 50 W | Highest VO2 in 20 s period | Breath-by-breath data were averaged over 5 s segments |
| Norouzi et al. [68] | ***Not reported*** | Highest VO2 in 30 s period | Breath-by-breath data were averaged over 5 s segments |
| Twist et al. [69] | ***Not reported*** | Highest VO2 in 30 s period | ***Not reported*** |
| Bossi et al. [70] | The warm-up consisted of three 7 min bouts consecutively at 60%, 70%, and 80% GET | Highest VO2 in 30 s period | Breath-by-breath data were averaged over 5 s segments |
| Odden et al. [38] | The first two steps of the Lamberts and Lambert Submaximal Cycle Test was used as warm‐up (6 min at 60%, and 6 min at 80% HRmax respectively) | Highest VO2 in two successive 30 s averages | Breath-by-breath data were averaged over 10 s segments |
| Urianstad et al. [71] | The warm-up consisted of 5 min at RPE 11, 5 min at RPE 13, 3 min at RPE 15, 2 min at RPE 9–10, 30 s at RPE 16, 30 s at 120% of PO40min, and finally, 4 min at RPE 9–10 | Highest VO2 in two successive 30 s averages | Breath-by-breath data were averaged over 10 s segments |
| Appelhans et al. [72] | ‘A standardised 20 min warm-up’, with duration and exercise intensities visualised in figure | Highest VO2 in 60 s period | Breath-by-breath data were averaged over 15 s segments |
| Çabuk et al. [74] | Participants pedalled at a cadence of 70–80 RPM against a load of 1 kg for 5 min. During the last 5-s of the 3rd, 4th, and 5th minutes, participants had to pedal at their highest possible cadence against a load of 2 kg | Highest VO2 in 10 s period | Breath-by-breath data were averaged over 5 s segments |
| Raimundo et al. [75] | ***Not reported*** | Highest VO2 in three successive 5 s averages | Breath-by-breath data were averaged over 5 s segments |
| Twist et al. [76] | ***Not reported*** | Highest VO2 in 30 s period | Breath-by-breath data were averaged over 30 s segments |
|  |  |  |  |
| **Jumping** |  |  |  |
| Ducrocq et al. [77] | No warm-up preceded the HIIT sessions to accurately determine the time to reach V̇O_2_max from the start of exercise | ***Not reported*** | Breath-by-breath data were averaged over 5 s segments |
|  |  |  |  |
| **Kayaking** |  |  |  |
| Paquette et al. [78] | ***Not reported*** | Highest VO2 in 30 s period | ***Not reported*** |
|  |  |  |  |
| **Rowing** |  |  |  |
| Patterson et al. [79] | A 5 min warm-up began with each subject rowing at 33% PPO for 3 min, and immediately increased to PPO | Highest VO2 in 30 s period | Breath-by-breath data were averaged over 15 s segments |
| Faelli et al. [80] | Rowers performed 10 min at 40% of their HRmax, followed by 5 min at the athlete’s preferred pace, and finished with 5 min at 10% PPO | VO2 of final 30 s | Raw breath-by-breath data was used to calculate outcome measures |
|  |  |  |  |
| **Running** |  |  |  |
| Billat et al. [34], and Billat et al. [81] | Participants warmed up for 15 min at 50% vVO2max | Highest VO2 in two successive 15 s averages | Breath-by-breath data were averaged over 5 s segments |
| Demarie et al. [33] | ***Not reported*** | Highest VO2 in 15 s period | Breath-by-breath data were averaged over 15 s segments |
| Dupont et al. [82] | The warm-up consisted of 10 min of continuous running at 60% MAS | Highest VO2 in four successive 15 s averages | Breath-by-breath data were averaged over 5 s segments |
| Millet et al. [83] and Millet et al. [84] | A 6 min warm-up at 75% of vVO2max was performed | Highest VO2 in 30 s period | Breath-by-breath data were averaged over 5 s segments |
| Dupont & Berthoin [85] | HIIT sessions were preceded by a standardised warm-up, involving a 10 min run at 10 km·h^-1^ | Highest VO2 in two successive 15 s averages | Breath-by-breath data were averaged over 5 s segments |
| Tardieu-Berger et al. [86], and Abderrahman et al. [97] | A standardised warm-up consisted of 10 min of continuous running at 12 km·h^-1^, followed by 5 min of stretching exercises and three short bursts of accelerations | Highest VO2 in two successive 5 s averages | Breath-by-breath data were averaged over 5 s segments |
| Midgley et al. [87] | The standardised warm-up that preceded the test consisted of 10 min  running at 9 km·h^-1^ followed by 5 min of light static stretching | Highest VO2 in two successive 15 s averages | Breath-by-breath data were averaged over 5 s segments |
| Midgley et al. [88] | The standardised warm-up consisted of 5 min running at 60% vVO2max followed by 5 min of light static stretching and a further 10 min of running at 60% vVO2max | Highest VO2 in two successive 15 s averages | Breath-by-breath data were averaged over 5 s and 15 s segments |
| Rozenek et al. [89] | Each HIIT trial was preceded by a 3 min warm-up period of running at  50% of vVO2max | Highest VO2 in 15 s period | Breath-by-breath data were averaged over 15 s segments |
| Thevenet et al. [37], Thevenet et al. [90], and Thevenet et al. [91] | Participants warmed up with 10 min of continuous jogging, followed by 5 min of their usual stretching, and five short acceleration bursts | Highest VO2 in two successive 15 s averages | Breath-by-breath data were averaged over 5 s segments |
| O’Brien et al. [92] | Each participant completed a 5 min treadmill warm-up run at 60% MAV | Highest VO2 in 60 s period | Breath-by-breath data were averaged over 30 s segments |
| Wakefield & Glaister [93] | Subjects completed a 5 min warm-up at 50% vVO2max | ***Not reported*** | Raw breath-by-breath data was used to calculate outcome measures |
| Zafeiridis et al. [94], and Zafeiridis et al. [95] | Participants performed a standardised warm-up that consisted of an 8 min  jog at 55%–60% MAV, and 4 min of stretching exercises | Highest VO2 in two successive 15 s averages | Breath-by-breath data were averaged over 5 s segments |
| Buchheit et al. [96] | Participants completed a standardised 5 min warm-up run at 50% vVO2max | Highest VO2 in 30 s period | Breath-by-breath data were averaged over 5 s segments |
| De Aguiar et al. [98] | The warm-up consisted of 10 min of continuous running at 65% MAV | Highest VO2 in 15 s period | Breath-by-breath data were averaged over 5 s segments |
| Panissa et al. [99] | The athletes performed a 5 min warm-up at 8 km·h^-1^ or 10 km·h^-1^ according to their training background | Highest VO2 in two successive 15 s averages | Breath-by-breath data were averaged over 5 s segments |
| Panasci et al. [100] | ***Not reported*** | VO2max was estimated | ***Not reported*** |
| Smilios et al. [101] | Each experimental session started with a warm-up involving running for 7.5 min at 60% MAV and 0.5 min at MAV, and then stretching | Highest VO2 in 30 s period | Breath-by-breath data were averaged over 5 s segments |
| Baquet et al. [102] | The warm-up involved a 3 min run at 7.5 km·h^-1^ | Highest VO2 in 15 s period | Breath-by-breath data were averaged over 15 s segments |
| Schoenmakers & Reed [103] | Participants performed a 6 min warm-up at 70% ΔGET on the cNMT, followed by a 9 min break | Highest VO2 in 30 s period | Breath-by-breath data were averaged over 1 s segments |
| Ducrocq et al. [77] | No warm-up preceded the HIIT sessions to accurately determine the time to reach V̇O_2_max from the start of exercise | ***Not reported*** | Breath-by-breath data were averaged over 5 s segments |
| Myrkos et al. [104] | Each exercise session started with a standardised warm-up that included running for 7 min at 60% MAV, then for 30 s at 90% MAV and after that for 30 s at MAV, followed by static stretching | Highest VO2 in 30 s averages | Breath-by-breath data were averaged over 5 s segments |
| Beltrami et al. [55] | Participants warmed up for 10 min at self-selected pace. The first work interval was then preceded by an additional 5 min warm-up with 3 min at 10 km·h^-1^, followed by 2 min at +50% of the difference between 10 km·h^-1^ and the intensity of the first interval | Highest VO2 in six successive 5 s averages | Breath-by-breath data were averaged over 5 s segments |
| José Arantes et al. [105] | Participants completed a 5 min warm-up at 65% MAV during all protocols, followed by two 5 s sprints at MAV with 30 s breaks between them | Highest VO2 in 30 s averages | Breath-by-breath data were averaged over 5 s segments |
| Germano et al. [106] | A standard warm-up was performed for 3 min and consisted of running at 10% below of ventilatory threshold | ***Not reported*** | Breath-by-breath data were averaged over 10 s segments |
| Sánchez-Otero et al. [107] | ***Not reported* –** ‘Participants performed a standardised warm-up at the beginning of each session’ | Highest VO2 in two successive 15 s averages | Breath-by-breath data were averaged over 5 s segments |
| Bok et al. [108] | Participants performed a standardised 5 min warm-up, comprised of continuous runs for 2 and 3 min at ventilatory threshold and respiratory compensation point | Highest VO2 in 30 s period | Breath-by-breath data were averaged over 5 s segments |
| Held et al. [109] | Participants performed a standardised 15 min warm-up, consisting of easy running, including knee lift, heel lift, external rotation hip, internal rotation hip, 10 lunges alternating, 10 squats, individual dynamic stretching | Highest VO2 in 30 s period | Breath-by-breath data were averaged over 1 s segments |
| Twist et al. [69] | ***Not reported*** | Highest VO2 in 30 s period | ***Not reported*** |
| Varela-Sanz et al. [110] | Subjects performed a standardised warm-up consisting of 10 min of running at 60% HRmax, 5 min of calisthenics, 5 sets of 50 m at a progressive velocity, and 5 CMJs on the force plate | Highest VO2 in two successive 15 s averages | Breath-by-breath data were averaged over 5 s segments |
| Vaccari et al. [111] | The warm up consisted of 10 min running at 10 or 12 km·h^-1^ | Highest VO2 in 30 s period (or VO2 of final 30 s) | Breath-by-breath data were averaged over 5 s segments |
| Fleckenstein et al. [112] | *Not reported* – ‘Each participant performed the warm-up in the same individualized manner’ | Highest VO2 in 15 s period | Breath-by-breath data were averaged over 5 s segments |
|  |  |  |  |
| **Swimming** |  |  |  |
| Bentley et al. [113] | ***Not reported*** | Highest VO2 in 30 s period | Breath-by-breath data were averaged over 5 s segments |
| Libicz et al. [114] | ***Not reported*** | Highest VO2 in 30 s period | Breath-by-breath data were averaged over 5 s segments |
| Almeida et al. [115], and Almeida et al. [116] | The warm-up protocol consisted of dry land stretching exercises for upper- and lower-limbs, and 800 m swimming at a comfortable and effortless pace (including whole-body, and only arms and legs swimming practices) | Highest VO2 in 30 s period | Breath-by-breath data were averaged over 1 s segments |
|  |  |  |  |
| **Cross Country Skiing** |  |  |  |
| Rønnestad et al. [117] | Participants warmed up for 5 min at 7 km·h⁻¹, after which the speed then gradually increased to their individual 4.0 mmol·L⁻¹ pace (for 4 min). Speed then rose to the first work interval’s starting velocity for 1.5 min, concluding with a 5 min active recovery. | Highest VO2 in two successive 30 s averages | Breath-by-breath data were averaged over 15 s segments |
| Rønnestad et al. [118] | The first 5 min of the warm-up was performed at an incline of 2% and a speed of 3 km·h^-1^, followed by a 5 min increase to 7 km·h^-1^ and 4%. Speed was then further increased for two min to 15 km·h^-1^ and an incline of 8%. A 5-minute active recovery period concluded the warm-up | Highest VO2 in two successive 30 s averages | Breath-by-breath data were averaged over 10 s segments |
| Rønnestad et al. ­­[119] | The first 5 min of the warm-up was performed at an incline of 3% and a speed of 5 km·h^-1^, followed by a 5 min increase in speed and incline (10 km·h^-1^, and 7%). Speed was then further increased for five min to 13 km·h^-1^. A 3-minute active recovery period concluded the warm-up | Highest VO2 in two successive 30 s averages | Breath-by-breath data were averaged over 20 s segments |
